# Bayesian multiple logistic regression for case-control GWAS

**DOI:** 10.1371/journal.pgen.1007856

**Published:** 2018-12-31

**Authors:** Saikat Banerjee, Lingyao Zeng, Heribert Schunkert, Johannes Söding

**Affiliations:** 1 Max Planck Institute for Biophysical Chemistry, Göttingen, Germany; 2 German Heart Centre, Munich, Germany; Emory University, UNITED STATES

## Abstract

Genetic variants in genome-wide association studies (GWAS) are tested for disease association mostly using simple regression, one variant at a time. Standard approaches to improve power in detecting disease-associated SNPs use multiple regression with Bayesian variable selection in which a sparsity-enforcing prior on effect sizes is used to avoid overtraining and all effect sizes are integrated out for posterior inference. For binary traits, the *logistic* model has not yielded clear improvements over the linear model. For multi-SNP analysis, the logistic model required costly and technically challenging MCMC sampling to perform the integration. Here, we introduce the *quasi-Laplace* approximation to solve the integral and avoid MCMC sampling. We expect the logistic model to perform much better than multiple linear regression except when predicted disease risks are spread closely around 0.5, because only close to its inflection point can the logistic function be well approximated by a linear function. Indeed, in extensive benchmarks with simulated phenotypes and real genotypes, our Bayesian multiple LOgistic REgression method (B-LORE) showed considerable improvements (1) when regressing on many variants in multiple loci at heritabilities ≥ 0.4 and (2) for unbalanced case-control ratios. B-LORE also enables meta-analysis by approximating the likelihood functions of individual studies by multivariate normal distributions, using their means and covariance matrices as summary statistics. Our work should make sparse multiple logistic regression attractive also for other applications with binary target variables. B-LORE is freely available from: https://github.com/soedinglab/b-lore.

## Author summary

In recent years, genome wide association studies (GWAS) have become the primary approach for identifying genetic variants associated with the origination of complex diseases. In case-control GWAS, the genotypes of roughly equal number of diseased (“cases”) and healthy (“controls”) people are compared to determine which genetic variants are significantly more frequent among cases. From the disease-associated variants we hope to get insights into how the disease develops. To find the disease-associated variants, a linear relationship between the disease risk and the number of minor alleles at the variant sites has usually been assumed, because the more appropriate sigmoid relationship requires slow and cumbersome sampling techniques. We found an efficient analytical approximation that renders sampling unnecessary and makes our multiple *logistic* regression model easy to train. We show that it outperforms the usually employed multiple *linear* regression model whenever nonlinearities become strong, which is the case, for example, when the numbers of case and control patients differ significantly. Therefore, novel genetic disease-associated variants could be found by adding controls to existing case-control GWAS and reanalyzing them with B-LORE.

## Introduction

Common, noninfectious diseases are responsible for over ⅔ of the deaths worldwide. Genome wide association studies (GWAS) have opened up a fundamentally new approach to identify novel regions of the genome which are associated with these complex human diseases. In the past decade, GWAS identified thousands of genetic variants, particularly single nucleotide polymorphisms (SNPs), associated with many diseases and complex traits [1, 2].

In a typical GWAS, genotype data comprising millions of SNPs from thousands of individuals with some trait are analyzed to identify SNPs that have significant associations with the trait. Most studies apply simple regression (single-SNP analysis) *i.e*., they test for one SNP at a time, yielding a *p*-value for each SNP. GWAS for quantitative traits like lipid levels, BMI, height, etc. use a linear model for regression of the trait by the minor allele counts of the SNP. Case-control GWAS, for which the binary trait is either “diseased” (“cases”) or “healthy” (“controls”), use a logistic model for regression.

Complex diseases studied with GWAS are usually polygenic, with many SNPs each contributing only a small fraction of the disease risk. The low effect sizes limit the power to detect statistically significant associations. Meta-analyses attempt to increase the statistical power by combining the summary statistics from single-SNP analyses of many GWAS, thereby encompassing a large number of samples, often in the range of hundreds of thousands.

While the simple regression model is computationally fast, it can only detect association and not statistical coupling or even causality. Therefore, a non-causal SNP (tag SNP) that is in strong linkage disequilibrium (LD) with a causal SNP will also obtain similarly significant *p*-values, making it difficult to decide which of these SNPs is really causal. Multiple regression models (also called multiple-SNP analyses or polygenic models) overcome this problem by using many SNPs at a genetic region or locus as explanatory variables. They can distinguish between correlation and coupling because the causal SNP can explain away the effects of other SNPs, which are merely correlated with the given phenotype via the causal SNP. Multiple regression can also improve the power of GWAS to detect risk loci by aggregating evidence from many SNPs with low effect sizes.

In GWAS, only a few hundred out of millions of measured or imputed SNPs are expected to be causal, *i.e*., to have a direct influence on disease risk. Therefore, Bayesian variable selection has been employed to prevent overtraining (see [3] for an overview). Bayesian variable selection uses a sparsity-enforcing prior on the effect sizes to force all but a small fraction of the regression coefficients to zero. Bayesian variable selection regression (BVSR) [4, 5] uses a point-normal prior, a mixture of a delta function at zero and a normal distribution for causal SNPs.

Until recently, multiple regression was limited by the requirement of individual-level genotype data. It was practically infeasible to apply it to multiple GWAS due to logistical, technical, and ethical restrictions for sharing huge volumes of genetic data from patients. In recent years, a number of studies devised novel strategies to perform multiple regression with variable selection using only summary statistics: PAINTOR [6, 7], CAVIAR [8], CAVIARBF [9] and FINEMAP [10] employ a linear model yielding a multivariate normal likelihood. Its mean is approximated using the single-SNP effect size estimates and its covariance matrix is deduced from the LD correlation matrix of the SNPs. Like BVSR, they use a point-normal prior for variable selection. These methods are routinely used for fine-mapping *i.e*., for prioritizing the SNPs within the risk-associated loci (see [11] for a recent review).

For binary phenotypes, these fine-mapping methods approximate the logistic likelihood with a linear function of the genotype vector, permitting an analytical solution of the integral over effect sizes. Because the integration is analytically intractable without this linear approximation, multiple *logistic* regression required computationally cumbersome Markov Chain Monte Carlo (MCMC) sampling. Early in 2009, Newcombe *et al*. developed a Bayesian framework for multiple logistic regression using variable selection [12] using full MCMC sampling of all parameters and analyzing ∼ 35 SNPs. For analyzing binary traits with BVSR [5], Guan and Stephens used the probit model, which is very similar to the logistic model. However, they could not demonstrate a clear benefit of the probit model and ascribed this to technical difficulties in the MCMC sampling (insufficient mixing for binary traits), concluding that the approach needs “further methodological innovation”.

Besides the methodological challenge of performing the integration over effect sizes, single-SNP logistic regression did not yield clear advantages over linear regression [13], which might have also reduced the interest of exploring multiple logistic regression. Here we argue that the reason for the lack of improvement is simply due to the fact that the risk explained by a single SNP is usually so low that predicted risks stay very near to 0.5, where a linear approximation of the logistic function is still very accurate.

In this work, we present B-LORE, a scalable Bayesian method for multiple logistic regression. We introduce the quasi-Laplace approximation in which we approximate the *L*_2_-regularized likelihood of the logistic model by a normal distribution, whose mean vector and covariance matrix serve as our novel summary statistics. This trick allows us to analytically integrate the (unregularized) likelihood times the point-normal effect-size prior over the unknown effect sizes. The regularization ensures that the mode of the regularized likelihood is near the mode of the integrand and hence the normal approximation stays accurate. We estimate the parameters of our effect-size prior by maximizing the total marginal likelihood over all loci. B-LORE can also combine multiple case-control GWAS because the maximization requires only the summary statistics for each study.

Through extensive benchmarks, in which we simulate binary phenotypes for real genotype data, we show that the quasi-Laplace approximation is significantly better than existing linear approximations of the logistic model. Most other multiple regression methods using Bayesian variable selection have been developed for fine-mapping of SNPs in regions which show evidence for containing at least one causal SNP. We therefore limit our comparison here to this application. However, B-LORE can also be employed to rank risk loci by their probability to contain a causal SNP or to predict the genetic risk of patients from their genotype.

## Materials and methods

We are interested in analyzing case-control GWAS, using summary data instead of individual genotype data. In this section, we describe the model and the implementation of B-LORE. At each step we compare and contrast B-LORE with other multiple regression variable selection methods, namely BIMBAM [4], piMASS [5], GEMMA [3, 14], CAVIARBF [9], FINEMAP [10] and PAINTOR [6, 7]. For quick reference, we summarized the methods in Table 1. Finally, we describe the data and simulation details used for the validation of our method.

**Table 1:** Comparison of methods for multiple regression of case-control GWAS data. All methods use the point-normal prior (Eq (4)) for the effect sizes of the SNPs. The posterior probability is obtained by integrating out these effect sizes, either via MCMC sampling or analytically (column 2). Column 3 compares the approximations to the logistic or probit likelihood model. Column 4 shows which hyperparameters of the prior distribution are estimated from the data, and the method of hyperparameter estimation is shown in column 5. Column 6 shows whether the method analyzes multiple loci together (“Yes”) or independently (“No”). Column 7 lists which tools can perform meta-analysis. MCMC: Markov Chain Monte Carlo sampling, EM: expectation-maximization, CG: conjugate gradient method.

Method	Integration method	Approximation	Hyperparameters (HP) learnt from data	Method for HP estimation	Multiple loci	Meta-analysis
BIMBAM [4]	MCMC	Laplace	None	–	No	No
piMASS [5]	MCMC	Probit	*π*_*i*_, *σ*	MCMC	No	No
GEMMA [3, 14]	MCMC	Probit	*π*_*i*_, *σ*	MCMC	No	No
CAVIAR [8]	Analytic	Linear	None	–	No	Yes
CAVIARBF [9]	Analytic	Linear	None	–	No	Yes
FINEMAP [10]	Analytic	Linear	None	–	No	Yes
PAINTOR [6, 7]	Analytic	Linear	*π*_*i*_	EM	Yes	Yes
**B-LORE**	Analytic	quasi-Laplace	*π*_*i*_, *σ*	CG	Yes	Yes

### Likelihood function

For binary traits, GWAS data consists of phenotypes *ϕ*_*n*_ ∈ {0, 1} (healthy or diseased) and of genotypes *w*_*ni*_ ∈ {0, 1, 2}, where 0, 1, or 2 signify the number of minor alleles of patient *n* ∈ {1, …, *N*} at SNP *i* ∈ {1, …, *I*}. The genotype is centered and normalized as $x_{ni}=(w_{ni}-2f_{i})/\sqrt{2f_{i}(1-f_{i})}$, where *f*_*i*_ is the minor allele frequency of the *i*^th^ SNP. We denote the vector of normalized genotypes for the *n*^th^ sample as **x**_*n*_, and the *N* × *I* matrix of genotypes as **X**.

As BIMBAM, CAVIARBF, FINEMAP and PAINTOR, we use standard logistic regression to model the probability for a patient to have the disease,

$$\begin{array}{c} p_{n}≔p(\phi_{n}=1|x_{n},\beta )=\frac{1}{1+\exp(-\beta^{\;T}x_{n})}\;. \end{array}$$ (1)

which can be transformed to

$$\begin{array}{c} \log\frac{p(\phi_{n}=1|x_{n},\beta )}{p(\phi_{n}=0|x_{n},\beta )}=\beta^{\;T}x_{n}\;, \end{array}$$ (2)

The minor allele count *x*_*ni*_ of SNP *i* contributes linearly to the log-odds ratio with an effect size *β*_*i*_. The likelihood for *N* patients is

$$\begin{array}{cc} L(\beta ) & =p(\phi |X,\beta )=\prod_{n=1}^{N}p_{n}^{\phi_{n}}{\left(1-p_{n}\right)}^{1-\phi_{n}}=\prod_{n=1}^{N}\frac{\exp(\phi_{n}\beta^{\;T}x_{n})}{1+\exp(\beta^{\;T}x_{n})}\;. \end{array}$$ (3)

For notational convenience, we have absorbed the offset term (a vector of **1**s) as the 0^th^ column of **x**_*n*_.

### Prior distributions

#### Point-normal prior for effect sizes

In a GWAS, the number of parameters *p* = *I* is usually much larger than the number of samples *N* (*p* ≫ *N*). Hence, a standard approach of maximizing the likelihood with respect to the effect sizes will lead to gross overtraining. One common solution is to add a regularization term to the log likelihood that will push most of the components of ***β*** to zero or near zero. From a Bayesian viewpoint, this is equivalent to maximizing the posterior distribution *p*(***β***|**X**), which is proportional to *p*(**X**|***β***)*p*(***β***), where *p*(***β***) is the prior distribution of effect sizes [15].

To reflect the prior expectation that an overwhelming majority of SNPs have a negligible effect on disease risk, we use the point-normal prior,

$$\begin{array}{c} p(\beta_{i}|\pi_{i},\sigma )=(1-\pi_{i})\delta_{0}+\pi_{i}\;N(\beta_{i}|0,\sigma^{2})\;, \end{array}$$ (4)

which is used in many tools, *e.g*. BIMBAM, piMASS, CAVIARBF, FINEMAP and PAINTOR. The normal distribution models the effect sizes for the rare, causal SNPs and the delta function models the non-causal SNPs. The hyperparameters *π*_*i*_ control the sparsity of the model and *σ*^2^ describes the variance of the effect sizes.

#### Prior probability of *π* and *σ*

In the simplest case the prior probabilities to be causal is the same for all SNPs, *π*_*i*_ = *π* = const. CAVIARBF and FINEMAP implicitly assumes that *π*_*i*_ = 1/*I*. B-LORE also assumes *p*(*π*) = const. However, improvements can be expected by making *π*_*i*_ depend on informative local genomic features or annotation tracks [7].

BIMBAM, PAINTOR, CAVIARBF and FINEMAP use fixed values of *σ* that can be specified by the user. PiMASS uses an intuitively appealing prior on *σ* with wider tails (see [5] for details). B-LORE implicitly assumes a much simpler prior *p*(*σ*^2^) = const to avoid computational complexity.

#### Causality configurations

We define *c*_*i*_ ∈ {0, 1} as the hidden indicator variables defining the underlying causality of the SNPs. Here, *c*_*i*_ = 1 indicates that SNP *i* is causal and *c*_*i*_ = 0 otherwise. To simplify notations, we define the vector $\sigma_{c}^{2}$, whose *i*^th^ component is $\sigma_{c,i}^{2}=c_{i}\sigma^{2}$. This allows us to reformulate Eq (4) as:

$$\begin{array}{c} p(\beta |\pi ,\sigma )=\sum_{c}(\prod_{i=1}^{I}\pi^{c_{i}}{\left(1-\pi \right)}^{\left(1-c_{i}\right)})N(\beta |0,\mathrm{diag}(\sigma_{c}^{2})) \end{array}$$ (5)

with the sum running over all 2^*I*^ possible *causality configurations* **c** ∈ {0, 1}^*I*^. Using

$$\begin{array}{c} p(c|\pi ,\sigma )=\prod_{i=1}^{I}\pi^{c_{i}}{\left(1-\pi \right)}^{\left(1-c_{i}\right)} \end{array}$$ (6)

we can write the prior on the effect sizes as,

$$\begin{array}{c} p(\beta |\pi ,\sigma )=\sum_{c}p(c|\pi ,\sigma )N(\beta |0,\mathrm{diag}(\sigma_{c}^{2}))\;. \end{array}$$ (7)

In this formulation, *p*(*c*_*i*_ = 1∣*π*, *σ*) gives the prior probability of the *i*^th^ SNP to be causal before observing the phenotype and genotype data, and ‖**c**‖_1_ gives the total number of causal SNPs in the model.

BVSR and CAVIARBF also use the same Bernoulli prior on **c** (Eq (6)). FINEMAP uses a general discrete distribution for the number of causal SNPs. However, it requires that the region to be analyzed includes at least one causal SNP, *i.e*., *p*(**c** = **0**) = 0. For binary traits, piMASS also has the same restriction.

### Inference

#### Fine-mapping

The posterior probability for SNP *i* to be coupled to the disease is obtained by summing the posterior probability over all causality configurations **c** for which SNP *i* is causal (*i.e*., *c*_*i*_ = 1):

$$\begin{array}{c} p(c_{i}=1|\phi ,X,\pi ,\sigma )=\sum_{c\;:\;c_{i}=1}p(c|\phi ,X,\pi ,\sigma )\;, \end{array}$$ (8)

also called the posterior inclusion probability (PIP). CAVIARBF, FINEMAP and BVSR also outputs the PIP.

#### Prediction of causal loci

The probability for a locus to be coupled with the disease phenotype is equal to the probability of the locus harboring at least one causally associated SNP. This is equal to 1 minus the probability of not containing a single causal SNP:

$$\begin{array}{c} {\mathrm{Pr}}_{\text{causal}}=p(\text{locus}\;\text{is}\;\text{causal}|\phi ,X,\pi ,\sigma )=1-p(c=0|\phi ,X,\pi ,\sigma )\;. \end{array}$$ (9)

CAVIARBF and FINEMAP output Bayes factor for the posterior probability that there is at least one causal variant in the region against the null model.

### Quasi-Laplace approximation

Both the above posterior inferences require computing

$$\begin{array}{c} p(c|\phi ,X,\pi ,\sigma )=\frac{p(\phi |X,c,\pi ,\sigma )p(c|\pi ,\sigma )}{\sum_{c^{\prime }}p(\phi |X,c^{\prime },\pi ,\sigma )p(c^{\prime }|\pi ,\sigma )} \end{array}$$ (10)

for all causality configurations **c**, which in turn requires computing

$$\begin{array}{c} p(\phi |X,c,\pi ,\sigma )p(c|\pi ,\sigma )\\ \;=p(c|\pi ,\sigma )\int p(\phi |X,\beta )p(\beta |c,\pi ,\sigma )d\beta \\ \;=p(c|\pi ,\sigma )\int p(\phi |X,\beta )N(\beta |0,\mathrm{diag}(\sigma_{c}^{2}))d\beta \;. \end{array}$$ (11)

The above integration also appears in the marginal likelihood (see below) used for the optimization of the hyperparameters (*π*, *σ*). It does not have an exact solution when using the likelihood function of the logistic model, given by Eq (3). In contrast to logistic regression, the linear regression has normally distributed likelihood function, admitting an exact solution (for example, see Protocol S1 of [4]).

BIMBAM approximates the integrand with a multivariate Gaussian using Laplace’s method. In this method, the parameters of the Gaussian are determined by finding the integrand’s mode (*e.g*. using gradient-based optimization) and setting the precision matrix to the Hessian at the mode. Unfortunately, the mode depends on **c** and (*π*, *σ*). So, one needs to determine the mode and precision matrix every time (*π*, *σ*) is changed. Not only does this require individual genotype data, but this also makes it computationally infeasible to learn (*π*, *σ*).

CAVIARBF and FINEMAP approximate *p*_*n*_ with a linear function of ***β***, which reduces the likelihood function of Eq (3) to a multivariate normal distribution with scaled variance (see Pirinen *et al*. [16] and Chen *et al*. [9] for details). This approximation becomes inaccurate as we move away from the mode of the likelihood, and unfortunately the region in ***β*** space which contributes most to the integrand (around the mode of the integrand) can be quite far from the mode of the likelihood.

We propose the “quasi-Laplace approximation” to solve the integration. We start by splitting the integrand into two factors—an *L*_2_-*regularized likelihood* that approximates the integrand (but does not depend on (*π*, *σ*) or **c**) and a correction term:

$$\begin{array}{c} p(\phi |X,\beta )N(\beta |0,\mathrm{diag}(\sigma_{c}^{2}))\\ \;=p(\phi |X,\beta )N(\beta |0,{\tilde{\sigma }}^{2}\text{I})\times \frac{N(\beta |0,\mathrm{diag}(\sigma_{c}^{2}))}{N(\beta |0,{\tilde{\sigma }}^{2}\text{I})} \end{array}$$ (12)

The regularized likelihood is the product of the likelihood function (Eq (3)) and a Gaussian *regularizer*, $N\left(\beta |0,{\tilde{\sigma }}^{2}\text{I}\right)$ which acts as an approximate, simple prior distribution—pulling the maximum of the regularized likelihood near to the mode of the integral, making it more accurate than the Laplace approximation. The $\tilde{\sigma }$ can be optimized on the data (see S1 Text for details) or can be specified by the user. Next, we approximate the regularized likelihood by a multivariate Gaussian:

$$\begin{array}{c} p(\phi |X,\beta )N(\beta |0,{\tilde{\sigma }}^{2}I)\propto N(\beta |\tilde{\beta },{\tilde{\Lambda }}^{-1})\;. \end{array}$$ (13)

The quasi-Laplace approximation can be motivated from its validity in the limit of *N* ≫ 1 and equal number of cases and controls (details in S1 Text for interested readers). From our simulations, we found that the approximation can be extended to unequal number of cases and controls. The regularized likelihood does not depend on (*π*, *σ*) or **c**. Hence, it would suffice to calculate it only once and use $\tilde{\beta }$ and $\tilde{\Lambda }$ as our summary statistics. We determine $\tilde{\beta }$ and $\tilde{\Lambda }$ by maximizing the regularized likelihood with respect to ***β***, and setting $\tilde{\beta }$ to the mode and $\tilde{\Lambda }$ to the negative of the Hessian matrix at the mode. The covariance ${\tilde{\Lambda }}^{-1}$ resembles the scaled LD matrix used for the logistic model in CAVIARBF, FINEMAP and PAINTOR, but includes an additional term from the regularizer (see S1 Text).

### Optimization of hyperparameters

In the same spirit as BVSR, CAVIARBF and FINEMAP, we calculate the *marginal likelihood function* [15],

$$\begin{array}{cc} mL(\pi ,\sigma ) & ≔p(\phi |X,\pi ,\sigma )\\ & =\int p(\phi |X,\beta ,\pi ,\sigma )p\left(\beta |\pi ,\sigma \right)d\beta \\ & =\sum_{c}p(c|\pi ,\sigma )\int p(\phi |X,\beta )N(\beta |0,\mathrm{diag}(\sigma_{c}^{2}))d\beta \;, \end{array}$$ (14)

where we use Eq (7) in the last step. In contrast to the classical maximum likelihood approach, in which the parameters ***β*** are optimized, this method integrates out the parameters ***β***. This is a crucial difference in practice, because it eliminates the need to learn a large number of parameters and thereby very effectively guards against overtraining. Also, by integrating out the parameters we avoid the errors incurred when fixing them to noisy point estimates.

In B-LORE, the integration in Eq (14) is solved by the quasi-Laplace approximation whereas other methods use a linear approximation of logistic model to solve the integration. The solution (see S1 Text) depends on $\tilde{\sigma }$, $\tilde{\beta }$ and $\tilde{\Lambda }$, which serve as our novel summary statistics.

In B-LORE, we use the conjugate gradient method to maximize the marginal likelihood function. BIMBAM, CAVIARBF and FINEMAP use fixed values of the hyperparameters (*π*, *σ*). PAINTOR learns *π* from the data. PiMASS and GEMMA learn both *π* and *σ* from the data using MCMC.

### Meta-analysis

Meta-analysis would require combining $\tilde{\sigma }$, $\tilde{\beta }$ and $\tilde{\Lambda }$ from multiple studies. For a single study, the likelihood is given by Eq (3). We can combine multiple independent studies simply by computing the total likelihood as the product of the likelihoods of each contributing study *s*:

$$\begin{array}{c} p(\phi_{1},\ldots ,\phi_{S}|X_{1},\ldots X_{S},\beta )=\prod_{s=1}^{S}p(\phi_{s}|X_{s},\beta ) \end{array}$$ (15)

The integrand in Eq (14) will now have a product over multiple logistic functions. We have the summary statistics ${\tilde{\sigma }}_{s}$, ${\tilde{\beta }}_{s}$ and ${\tilde{\Lambda }}_{s}$ from each study. We apply the quasi-Laplace approximation for each study and combine the regularized likelihoods to a multivariate normal distribution:

$$\begin{array}{cc} \prod_{s=1}^{S}[p(\phi_{s}|X_{s},\beta )N(\beta |0,{\tilde{\sigma }}_{s}^{2}I)] & \propto \prod_{s=1}^{S}[N(\beta |{\tilde{\beta }}_{s},{\tilde{\Lambda }}_{s}^{-1})]\\ & =N(\beta |\tilde{\beta },{\tilde{\Lambda }}^{-1}) \end{array}$$ (16)

where we now have,

$$\begin{array}{cc} \tilde{\Lambda } & =\sum_{s=1}^{S}{\tilde{\Lambda }}_{s}\;\;\text{and}\;\;\tilde{\beta }={\tilde{\Lambda }}^{-1}\sum_{s=1}^{S}{\tilde{\Lambda }}_{s}{\tilde{\beta }}_{s}\;. \end{array}$$ (17)

We now use Eq (17) in place of Eq (13) to calculate the marginal likelihood for the optimization of hyperparameters. Unlike conventional meta-analysis methods, which pool aggregate allele count data of each individual SNP, the above method allows us to combine information from multiple regression.

### Overview of steps used in B-LORE software

In summary, B-LORE works in two steps:

1. *Novel summary statistics*. Calculation of summary statistics in B-LORE requires two optimization at each cohort or study:
  - Learn $\tilde{\sigma }$ from the data.
  - Learn $\tilde{\beta }$ and $\tilde{\Lambda }$ from the data.
2. *Meta-analysis*. Estimation of the hyperparameters (*π*, *σ*) by optimizing the marginal likelihood using $\tilde{\sigma }$, $\tilde{\beta }$ and $\tilde{\Lambda }$ of each cohort or study.

In our software, the first step can be run using the command --summary and the second step can be run using the command --meta.

### Factorization over loci

To speed up B-LORE analysis, we recommend to pre-select loci with a faster method such as SNPTEST [17–19] and to include SNPs from these pre-selected loci. Usually these candidate loci will be in linkage equilibrium since LD is highly local. Therefore the covariance matrix **X**^**⊤**^**X** is approximately block-diagonal, with each block corresponding to a locus. All multiple regression methods utilize this block-diagonal feature of the LD matrix. For example, BIMBAM uses a factorization over loci to perform multiple regression at each locus independently.

However, analyzing multiple loci together increases power and specificity for fine-mapping [20]. We expect the logistic model to benefit from analyzing multiple loci because they can together explain a higher fraction of heritability. Hence, we compute the summary statistics over all the pre-selected loci together. In S1 Text, we show that the marginal likelihood in Eq (14) can be factorized as a product over all the loci. Therefore, the hyperparameter optimization can be performed over all loci simply by summing the log marginal likelihoods of all loci. This will effectively ignore the off-diagonal terms in the covariance matrix among the loci but retain the important diagonal elements.

### Data

We used the genotype from five German population cohorts: German Myocardial Infarction Family Study (GerMIFS) I–V [21–26]. Details for quality control and pre-processing of these data were described by Nikpay *et al*. [26]. Briefly, there are a total of 6234 cases and 6848 controls of white European ancestry. Each cohort was imputed with phased haplotypes from the 1000 Genomes Project. SNPs were filtered for MAF > 0.05 and Hardy-Weinberg equilibrium (HWE) *p*-value > 0.0001.

### Ethics statement

The GerMIFS study subject recruitment was approved by the local ethics committees (University of Regensburg Ethics Committee, approval number 02/042 and University of Lübeck Ethics Committee, approval number 04-041) and all subjects gave their written informed consent prior to participation. The UK Biobank data were obtained from third party sources and no additional ethical approval was required.

### Phenotype simulation

The inherent complexity of the genotype data with strong linkage effects are very difficult to simulate realistically from haplotype data. We therefore used real patient genotypes for our semi-synthetic benchmark. We selected 100 genomic regions or loci from each of the five GerMIFS cohorts, such that each locus had 200 unique SNPs.

Since piMASS cannot do meta-analysis, we had to make a *unified* cohort by combining the individual genotypes and phenotypes of the five cohorts. Hence, we pruned each locus to contain only SNPs which are common to all the five cohorts, leaving 17218 SNPs distributed over the 100 loci.

In each locus, we sampled one or more SNPs to be causal. S1 Fig shows the distribution of the number of causal SNPs at each locus. Once we established a “ground truth” of *C* causal SNPs, we used the classical liability threshold model [27, 28] to simulate the binary phenotypes *ϕ*_*n*_ for every individual. Guan and Stephens [5] also used the same method for simulating binary phenotypes. The model assumes that the binary disease status results from an underlying continuous disease liability that is normally distributed in the population. If the combined effects of genetic and environmental influences push an individual’s liability across a certain threshold level, the individual is affected.

$$\begin{array}{cccc} \text{Liability} & \;y_{n} & & =\sum_{i=1}^{C}\beta_{i}x_{in}+\varepsilon_{j}\\ \text{Disease}\;\text{status} & \;\phi_{n} & & =\{\begin{array}{cc} 1 & \text{if}\;y_{n}>0\\ 0 & \text{if}\;y_{n}<0 \end{array} \end{array}$$ (18)

This is equivalent to a disease prevalence of 0.5 and gives roughly equal number of cases and controls. A lower prevalence of 0.01 does not affect the results (S9 Fig). The individual effect sizes *β*_*i*_ were assumed to be normally distributed $\beta_{i}∼N\left(0,h_{g}^{2}/\sqrt{C}\right)$ such that the causal SNPs aggregated to explain a fixed heritability ($h_{g}^{2}$, proportion of the phenotypic variance) on the liability scale.

$$\begin{array}{c} \mathrm{Var}(\sum_{i=1}^{C}\beta_{i}x_{in})=\sum_{i=1}^{C}\beta_{i}^{2}=h_{g}^{2}\;. \end{array}$$ (19)

The environmental contribution given by *ε*_*j*_ was assumed to be normally distributed $\varepsilon_{j}∼N\left(0,1-h_{g}^{2}\right)$. The observations on the risk scale follows a probit function of the liabilities on the unobserved continuous scale [28].

### Methods for comparison

Multiple regression methods are primarily used for fine-mapping, and are generally applied to regions (loci) which show evidence of containing at least one causal SNP. Hence, we compared the ranking of SNPs within each locus. Fine-mapping methods are generally assessed in terms of recall, *i.e*., the proportion of all causal SNPs in the locus included in the top ranked SNPs. In addition, we also looked at the precision, *i.e*., the proportion of causal SNPs among the selected ones.

#### B-LORE

We calculated the summary statistics at each cohort and performed meta-analysis. B-LORE is not designed to be applied on a single locus. It learns the hyperparameters by conjugate gradients from the data and would require enough number of SNPs for proper estimation. A single locus generally do not have enough causal SNPs in real data. Hence, all loci were used for the meta-analysis in each simulation, unless otherwise stated. The SNPs were ranked by the PIPs within each individual locus.

#### META

We used META v1.7 for single-SNP meta-analysis. At each individual cohort, we obtained summary statistics with SNPTEST v2.5.2 assuming an additive model and using a missing data likelihood score test (-method score). We then corrected each cohort for the genomic inflation factor, and performed meta-analysis using inverse-variance method based on a fixed-effects model. We used the −log_10_ (*p*) values for ranking.

#### FINEMAP

We used FINEMAP v1.1 for multiple regression with a linear approximation to the logistic model. It has similar accuracy as CAVIARBF and higher accuracy than PAINTOR without functional annotations. As input, FINEMAP requires the *z*-score for every SNP and the LD matrix for each locus. We calculated $z_{i}={\hat{\beta }}_{i}/\text{SE}({\hat{\beta }}_{i})$ using the results of META. The prior standard deviation was set as $s_{\lambda }=0.05\sqrt{\phi (1-\phi )}$, where *ϕ* is the proportion of cases among the *N* individuals. To ensure the best performance, we calculated the LD matrix using LDSTORE [29] from the single *unified* cohort. After analysis, we used the logarithm of the Bayes factors, log_10_ (BF(*c*_*i*_ = 1:*c*_*i*_ = 0)) for ranking the SNPs.

#### BVSR

GEMMA and piMASS both provide a BVSR probit model implementation using MCMC integration. The probit model is similar to the logistic model. We performed MCMC sampling at each locus of the *unified* cohort using piMASS with the -cc flag. We used 100000 burn-in steps and 1000000 production steps for the MCMC. We also repeated the same analysis without the -cc flag to compare the probit model with the linear model. We again used the PIPs for ranking the SNPs.

## Results

To evaluate the validity, accuracy and utility of the quasi-Laplace approximation, we performed a series of simulations to explore different conditions.

### Effect of heritability

**Fig 1:**
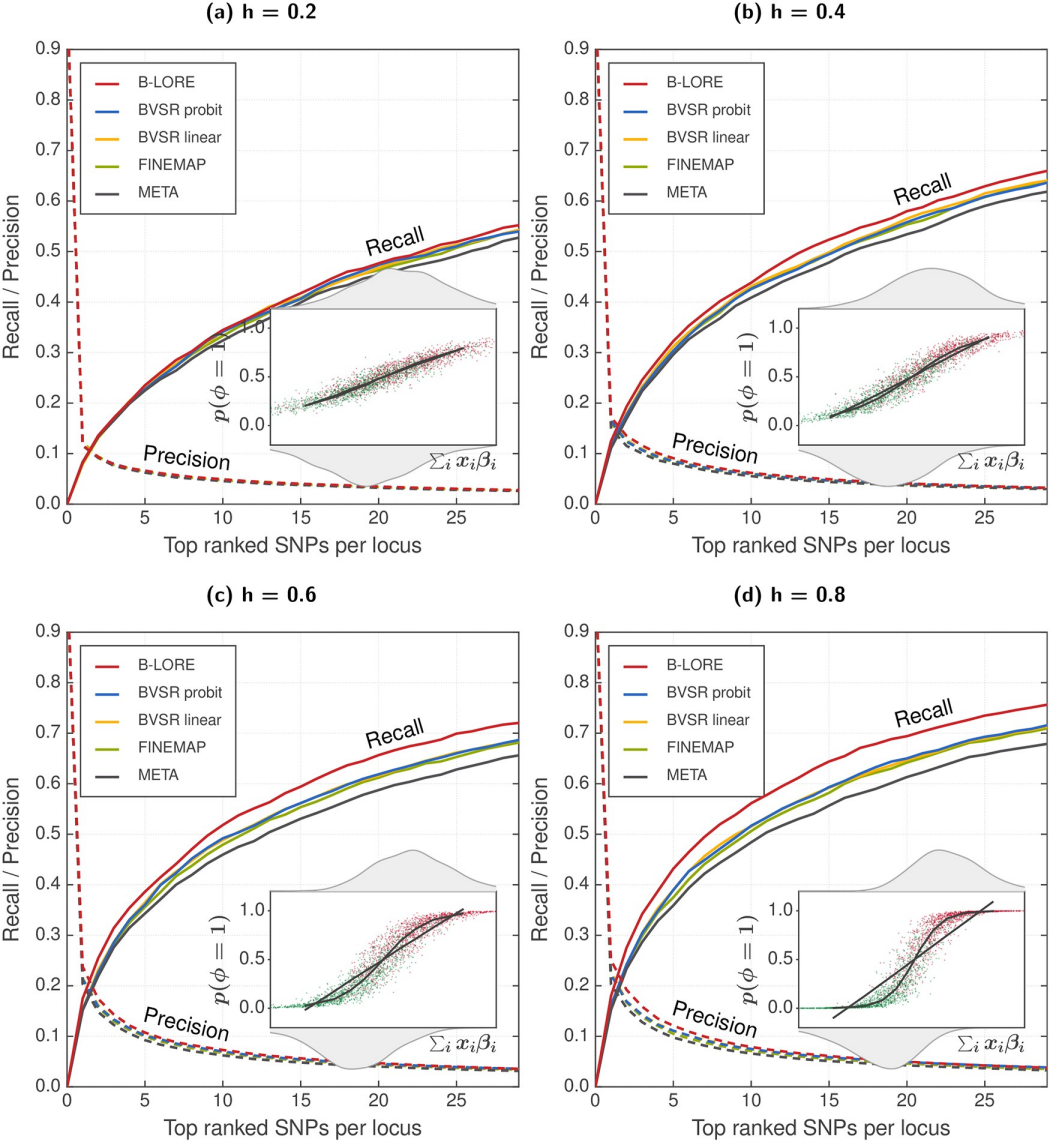
Multiple logistic regression improves fine-mapping in case-control GWAS. We simulated 13082 phenotypes using 100 loci of ∼200 SNPs, as described in the main text. We compared the ranking of SNPs at each locus using recall (solid lines, left *y*-axis) and precision (dotted lines, right *y*-axis), which were averaged over 100 loci and 20 simulation replicates. All methods were run with a maximum of two causal SNPs per locus. Panels (a)–(d) show the results at different heritabilities, $h_{g}^{2}=0.2,0.4,0.6\;\text{and}\;0.8$. Insets schematically compare the logistic model with the linear model. We plot the true ∑_*i*_ *x*_*i*_ *β*_*i*_ from the simulation for each individual along the *x*-axis, and show the distribution of cases and controls on the top and bottom axes respectively. On the *y*-axis, we show the predicted probability of being causal using a logistic model (*p*(*ϕ* = 1), red for cases and green for controls). The black lines are quantile averages of the linear predictor and the logistic predictor. With increasing heritability, the predicted disease probability spreads away from 0.5, where the logistic model becomes increasingly better than the linear model to explain the data and B-LORE shows increasingly more recall over other methods. The improvement by B-LORE over other multi-SNP analyses is more significant than the improvement by multi-SNP over single-SNP analyses.

First we show that B-LORE outperforms existing fine-mapping methods for binary phenotypes in case-control GWAS (Fig 1). In general, the recall of every method improves with heritability. At all heritabilities, B-LORE provides the best ranking of SNPs followed by the multiple regression methods (BVSR and FINEMAP), which are better than single-SNP meta-analysis (META). The difference in recall between B-LORE and BVSR increases with increasing heritability—the improvement being always equal to or better than the difference between BVSR and META.

We schematically compare the logistic model against the linear model for our simulated GWAS (insets of Fig 1). At low heritability, *e.g*. $h_{g}^{2}=0.2$, the cases and controls appear near the inflection point of the logistic curve, *i.e*., in the linear regime, and hence the linear model is a good approximation of the logistic model. With increasing heritability, the cases and controls have a wider spread and increasingly more samples appear in the non-linear regime. Hence, the linear model becomes increasingly inaccurate for explaining the data. Therefore, the quasi-Laplace approximation performs better than the linear approximation of the logistic model.

B-LORE also outperforms the BVSR probit model. We kept B-LORE as similar as possible to the BVSR probit model in order to validate the quasi-Laplace approximation—using the same priors for ***β*** and **c** and a simpler hyperprior for *σ*. There are two major differences in the implementation. First, piMASS (BVSR) uses MCMC for optimization while B-LORE uses conjugate gradient method including the quasi-Laplace approximation. Second, piMASS (BVSR) analyzes each locus independently while B-LORE uses multiple loci. Therefore, the poorer performance of BVSR compared to B-LORE could be due to: (1) inefficient MCMC sampling by BVSR for binary phenotypes (as postulated by the authors), or (2) less information in each individual locus, as compared to the total heritability accessible to B-LORE from multiple loci (as postulated by Newcombe *et al*. [20] in a different context).

In the following we try to explore the contribution of the logistic model and of multiple loci for the improved performance of B-LORE.

### Effect of case-control imbalance

We reasoned that if the improved performance of B-LORE is due to the more accurate modelling of risk in the non-linear regime, then B-LORE should also excel when the bias constant is far from zero, *i.e*., for case/control ratios less than 1.0. Having more controls than cases would lead to extreme imbalance in the data, making the linear approximation grossly suboptimal.

**Fig 2:**
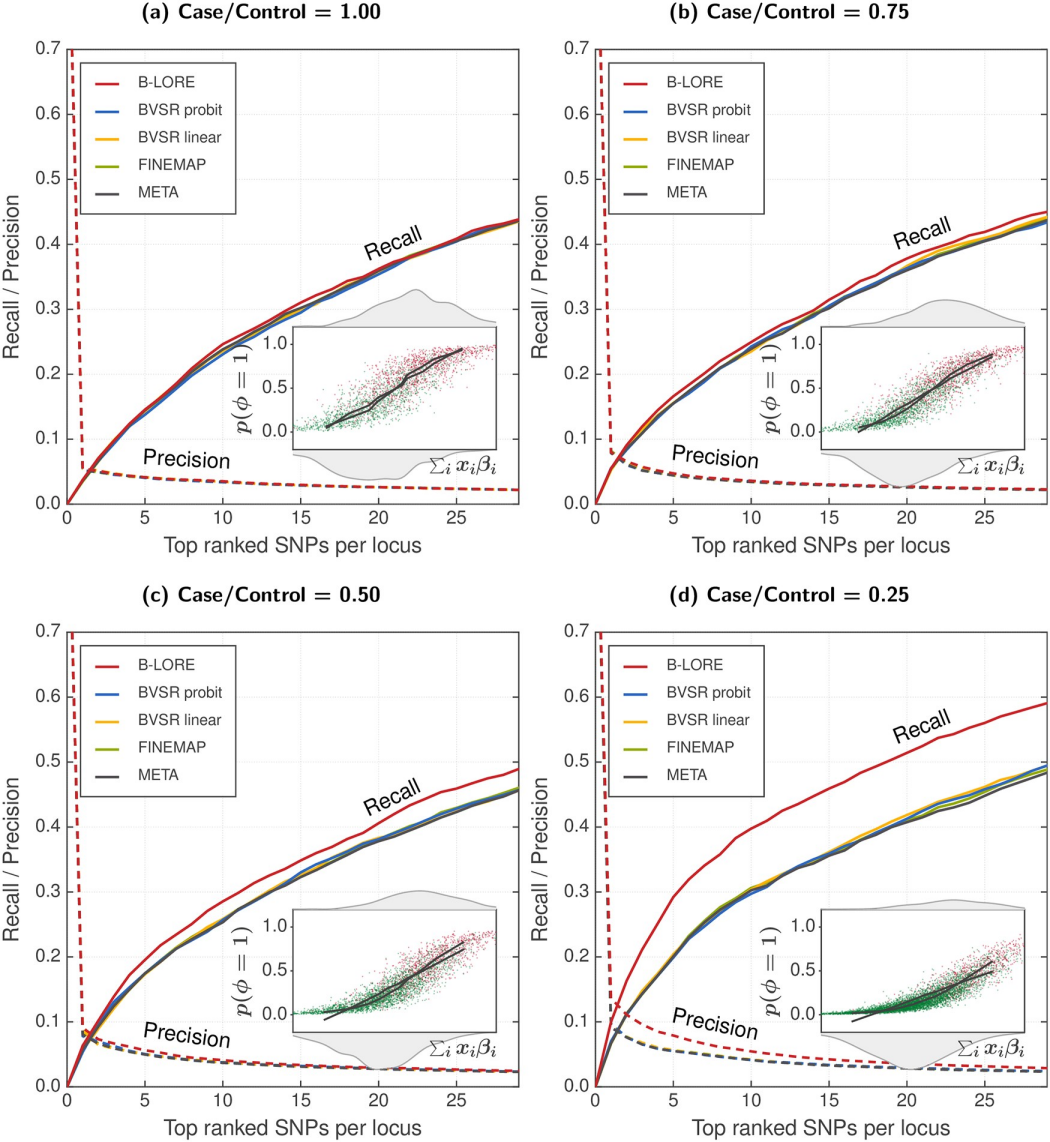
Multiple logistic regression improves power of GWAS with additional controls. We simulated phenotypes with varying case/control ratio—(a) 1625/1625, (b) 1625/3250, (c) 1625/4875 and (d) 1625/6500 respectively—using 100 loci of ∼200 SNPs, as described in the main text. All simulations used $h_{g}^{2}=0.4$. We compared the ranking of SNPs at each locus using recall (solid lines, left *y*-axis) and precision (dotted lines, right *y*-axis), which were averaged over 100 loci and 20 simulation replicates. All methods were run with a maximum of two causal SNPs per locus. Insets schematically compare logistic model with linear model (see Fig 1 for details). B-LORE shows increasingly more recall over other methods with addition of more controls, *i.e*., decreasing case/control ratio, because the logistic function becomes increasingly better than the linear function to model the data.

Our simulation yields roughly equal number of cases and controls—approximately ∼6500 cases and ∼6500 controls with a disease prevalence of 0.5. To obtain unequal cases and controls, we picked a subset of cases and/or controls by random choice. This can be done in two ways: (a) using the same number of cases (Fig 2), and (b) using the same number of controls (S3 Fig). The remaining cases and/or controls were ignored by assigning unknown (NA) disease status.

In both scenarios (a) and (b), B-LORE provides increasingly more recall over other methods as the case/control ratio decreases, confirming that the quasi-Laplace is more accurate than the existing linear approximations for the logistic model. Scenario (a) starts with 1625 cases and 1625 controls for 1:1 ratio. Due to less number of total samples, all methods have reduced power as compared to Fig 1b. All methods provide almost similar recall for 1:1 case:control ratio. Including additional controls improves the recall of all methods but B-LORE gets the maximum benefit. When the number of controls is four times as much as the number of cases (case/control = 0.25), the logistic model has 33% better recall than the linear model for choosing the top 10 SNPs (and 45% better recall for choosing the top 5 SNPs).

In scenario (b), all methods have maximum recall when case/control = 1.0. With reducing number of cases, BVSR, FINEMAP and META lose power, while B-LORE remains virtually unaffected.

In Fig 2a, all methods have similar accuracy. Keeping all other conditions constant, if we add more controls the performance of B-LORE improves considerably (Fig 2d). Adding more controls does not change the total heritability. Therefore, the improved performance of B-LORE in Fig 2d can be attributed to the logistic function which models the data much better than the linear function used by other methods.

However, following the same argument, the BVSR probit model should have been increasingly better than the BVSR linear model. We did not further investigate why their performances were similar. The authors of the BVSR implementation in the piMASS software noted that the sampling was inefficient for binary traits [5].

### Effect of multiple loci

We then wanted to check the effect of using multiple loci on the improvement in B-LORE. Multiple loci together explain a higher proportion of heritability than each single locus. Newcombe *et al*. earlier showed that multiple-locus analysis is better at fine-mapping than many independent single-locus analyses [20]. Unfortunately, their method is limited to quantitative phenotypes and is not yet designed for binary phenotypes. Hence we could not include their method in our benchmark. Instead, we varied the number of loci in our analysis to directly check the effect of estimating causality from multiple loci.

We simulated phenotypes for the five cohorts with 25, 50, 75 and 100 loci—with 80, 40, 33 and 20 simulation replicates respectively. Each locus had ∼200 SNPs. We used a fixed heritability of $h_{g}^{2}=0.6$ explained by these loci and 1:1 ratio of cases to controls.

**Fig 3:**
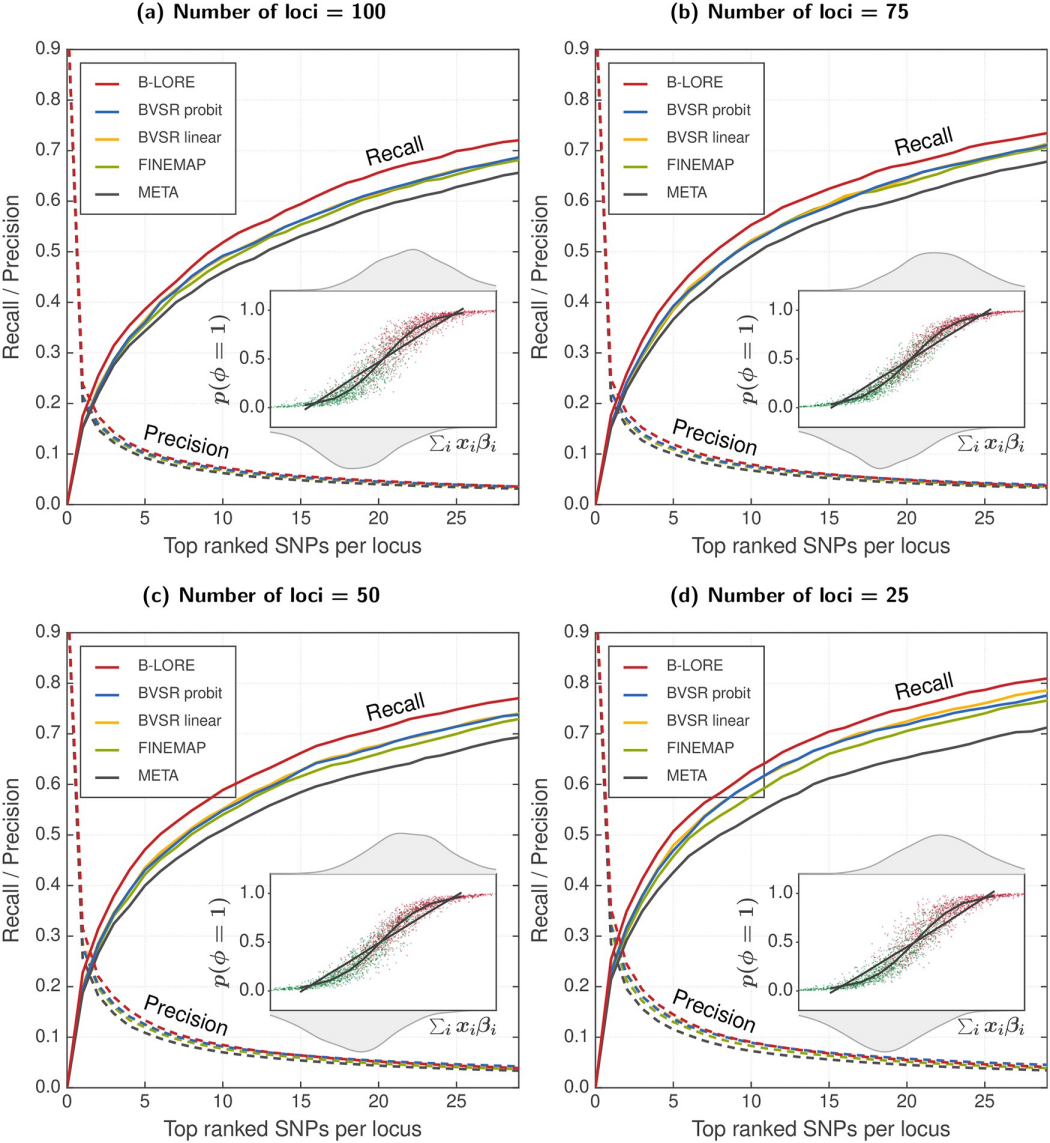
The advantage of B-LORE does not depend on the number of loci used for estimation. Panels (a)—(d) show results from simulations using 25, 50, 75 and 100 loci respectively. As described in the main text, we used 13082 samples and each locus had ∼200 SNPs. All simulations used total heritability of $h_{g}^{2}=0.6$ and hence the heritability per locus is different for the different panels. We compared the ranking of SNPs at each locus using recall (solid lines, left *y*-axis) and precision (dotted lines, right *y*-axis), which were averaged over the loci and the simulation replicates. All methods were run with a maximum of two causal SNPs per locus. Different panels show the results at different number of loci. Insets schematically compare logistic model with linear model in one simulation (see Fig 1 for details). The heritability per locus increases when the number of loci is reduced. Multiple regression becomes increasingly better than single SNP analysis, but the advantage of B-LORE over other multiple regression methods does not change with the number of loci. Note also that the comparison between logistic model and the linear model in the insets does not change with the number of loci.

In Fig 3 we show the fine-mapping performance of the different methods in these simulations. The heritability per locus increases with decreasing number of loci. Hence we observe that the performance of all methods improves when the number of loci is reduced. However, B-LORE always provides higher recall than other methods. Therefore, we conclude that the advantage of B-LORE does not depend on the number of loci, as long as all these loci explain the total heritability.

We further wanted to decouple the effect of multiple loci on calculating the summary statistics and meta-analysis of B-LORE. For this, we assumed that the phenotype is simulated from 100 loci as usual, and asked the following two questions. First, what happens if the B-LORE summary statistics are calculated from a subset of these 100 loci? This would mean that the heritability *visible* to B-LORE (*i.e*., the heritability explained by the chosen subset of loci) is reduced, *e.g*. calculating summary statistics with 25 loci would correspond to a heritability of 0.15 when the total $h_{g}^{2}=0.6$. Hence the results (S7 Fig) are similar to Fig 1. Second, what happens if the summary statistics are calculated from 100 loci for each cohort but the meta-analysis uses only a subset of them? In S8 Fig, we show that the ranking in each locus does not depend on the number of loci in the subset, indicating that the number of loci does not impact the estimation of hyperparameters, as long as we have enough number of causal SNPs (∼ 20) in the data for estimating the hyperparameters. Therefore, we can conclude that our method benefits from the greater proportion of heritability explained by the multiple loci.

### Number of causal SNPs allowed

Having established that B-LORE improves the power of case-control GWAS over existing methods, we next explored the effect of allowing different numbers of causal SNPs ‖**c**‖_1_ in the model of B-LORE. Different loci are expected to have different number of true causal SNPs in the data. Current implementation of B-LORE allows only one common input ‖**c**‖_1_ for all loci.

**Fig 4:**
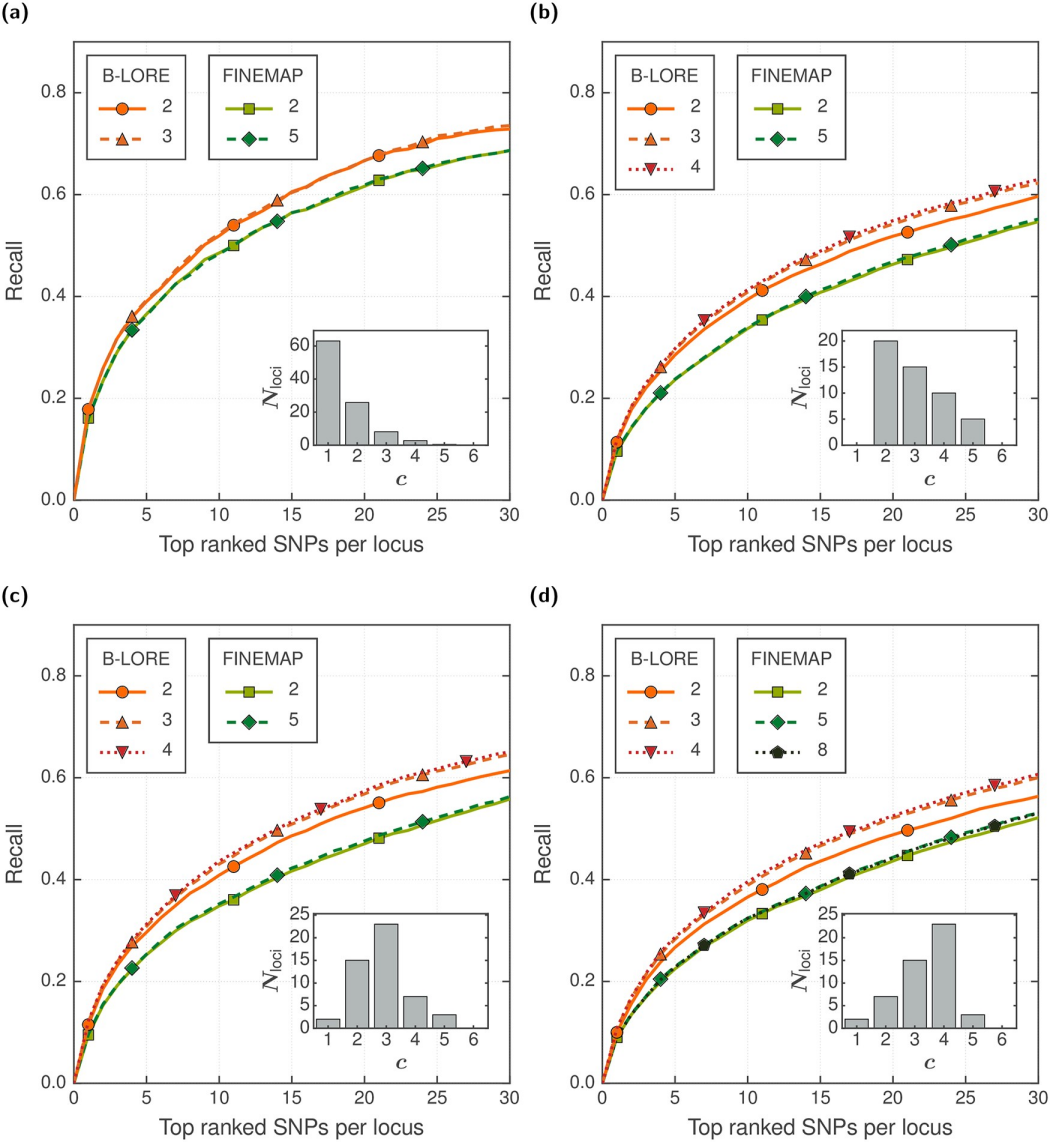
Effect of number of causal SNPs in B-LORE fine-mapping accuracy. We simulated 13082 phenotypes using 100 loci of ∼200 SNPs, as described in the main text. Panels (a)—(d) show the results using different hypothetical distributions of true causal SNPs in each simulation The distributions of the true causal SNPs were generated *ad hoc* and are shown in the inset of every panel. All simulations used $h_{g}^{2}=0.6$. We compared the ranking of SNPs at each locus by B-LORE and FINEMAP using recall (solid lines, left *y*-axis) averaged over the loci and the simulation replicates. Both methods were run with different number of causal SNPs allowed in the model (‖**c**‖_1_, see legends). FINEMAP was run on each locus separately and B-LORE was run on all loci together. For each method, we stopped increasing ‖**c**‖_1_ if the recall did not improve. The symbols are merely visual guides to distinguish between the different methods.

There is no way to know the “ground truth”, *i.e*., the distribution of true causal SNPs in the different loci of real GWAS. Here, we use different hypothetical distributions of true causal SNPs to simulate the phenotype and check the effect of ‖**c**‖_1_ on the ranking performance of B-LORE (Fig 4). We plot the hypothetical distribution of true causal SNPs used for each simulation in the insets of each panel respectively. In general, higher **c** improves the ranking power of B-LORE, but does not significantly impact FINEMAP. Of course, the impact depends on the “ground truth”. For most practical cases, when there are few or no regions with > 5 true causal SNPs, ‖**c**‖_1_ = 3 suffices for B-LORE.

Earlier studies showed stronger influence of ‖**c**‖_1_ on fine-mapping accuracy (*e.g*. see Fig. 5 of [10]). However, those simulations used replicates of a single locus with fixed number of true causal SNPs. In contrast, we used many loci with a distribution of true causal SNPs because we expect that future applications of B-LORE would involve analyses over all loci found in a GWAS.

### Computational efficiency

In S5 Fig, we show the average time and memory required for calculating the summary statistics of B-LORE in different realistic situations. For instance, computing the summary statistics of 40000 SNPs spread over 200 loci in a cohort of 10000 individuals requires around 2 hours and ∼22 GB of memory on an Intel Xeon E5-2670 v2 processor with 8 cores.

For B-LORE meta-analysis, we compare the average time and memory requirements of B-LORE with other multiple regression variable selection meta-analysis methods (CAVIARBF and FINEMAP) in S6 Fig. B-LORE has a speed comparable to CAVIARBF, although, unlike CAVIARBF, B-LORE optimizes the hyperparameters.

B-LORE is significantly slower than FINEMAP, and requires much more memory than FINEMAP. This is expected because B-LORE performs exhaustive search over the causality configurations, while FINEMAP uses a much faster shotgun stochastic search. The speed improvement by FINEMAP was a major technical breakthrough in multiple regression. We currently focus on better performance and only use a naive branch-and-bound algorithm (S1 Text) to reduce the search space. Note that unlike FINEMAP, B-LORE learns *σ* from the data.

Like other multiple regression methods, B-LORE is not designed for genome-wide analysis. It could be used to finemap each locus as well as re-rank the loci according to their probability of being causal (see Inference). Considering the speed and memory requirements (S5 and S6 Figs), we showed that with modest computing facilities, it is possible to apply B-LORE even on a total of 40000 SNPs, distributed evenly over 200 loci. The total number of SNPs and the total number of loci can be increased with more computing power, which is commonly available. There has to be a balance between the number of SNPs (*I*_*l*_) within a locus and the number of causal SNPs (‖**c**‖_1_) used by the model because the exhaustive search over the SNPs creates combinatorially increasing causal configurations depending on *I*_*l*_ and ‖**c**‖_1_. For example, *I*_*l*_ can be up to a few thousands with ‖**c**‖_1_ = 2, but *I*_*l*_ can only be up to a few hundreds with ‖**c**‖_1_ = 5. Different loci might have different number of causal SNPs, and we showed that ‖**c**‖_1_ = 3 is sufficient for a wide variety of distributions (Fig 4).

### Calibration of posterior inclusion probabilities (PIPs)

Finally, we note that the PIPs obtained from B-LORE are well-calibrated (S4 Fig). Following the method proposed by Guan *et al*. [5], we assessed that the PIPs obtained from B-LORE roughly correspond to the marginal precision. On null data, B-LORE does not show any spurious association (S2 Fig).

### Real data analysis: GWAS for coronary artery disease (CAD)

We applied B-LORE to analyze a GWAS study to identify genetic variants associated with coronary artery disease (CAD). The data comes from five small cohorts of German Myocardial Infarction Family Study (GerMIFS) I–V [21–26]. Details for quality control and pre-processing of these datasets were described by Nikpay *et al*. [26]. Briefly, there were a total of 6234 cases and 6848 controls with white European ancestry. Each cohort was imputed with phased haplotypes from the 1000 Genomes Project. SNPs were filtered for MAF > 0.05 and Hardy-Weinberg equilibrium (HWE) *p*-value > 0.0001.

In single-SNP analyses, results are usually summarized with the strongest marginal evidence for association. However, in a multi-SNP analysis, it can be misleading to focus on the SNPs with the largest posterior inclusion probabilities (PIPs). For example, if many SNPs are highly correlated there might be considerable uncertainty about which SNP is actually associated: None of the individual PIPs may be large, but the posterior probability of at least one SNP being included in the model would be high. Therefore, we summarize the results at the level of *regions* or loci, using Pr_causal_ (see Eq (9)). We then proceed to show fine-mapping results in these regions using the PIPs.

The GerMIFS cohorts were also used in a large meta study of CAD GWAS, organized by CARDIoGRAMplusC4D Consortium [26], which found 58 significant loci harboring SNPs in genome-wide significant association with CAD. The study leveraged the power from meta-analysis of ∼ 185,000 CAD cases and controls, while the GerMIFS cohorts have only ∼ 13,000 CAD cases and controls. Since then, several other larger studies have been performed and more than one hundred loci have been discovered to be associated with CAD [30–32].

We started with a traditional single-SNP meta-analysis using META on the SNPTEST summary statistics of the five cohorts, and found 3 loci to be genome-wide significant, namely the 9p21 locus on chr9, PHACTR1 and SLC22A3-LPAL2-LPA loci on chr6. From this meta-analysis, we ranked the SNPs according to their *p*-values and selected all the nominally significant SNPs with *p*-values < 5 × 10^−5^. We grouped these SNPs together based on their genomic positions. SNPs which were spatially close (within ± 200 kb) were included in the same locus. We then used the top 50 groups, and defined each locus by collecting the top 400 SNPs (ranked according to their *p*-values) within ± 200 kb regions of each lead group.

We used these 50 loci for meta-analysis with B-LORE. In practice however, one can use as many loci as desired and use more sophisticated definition of a locus, for example, by considering LD blocks. We generated summary statistics for each of the 5 cohorts and combined them using B-LORE meta-analysis. For the meta-analysis we allowed a maximum of five causal SNPs per locus.

Since the ground truth is unknown for real data, we did an extensive blinded literature search for all these 50 loci. For details about the genomic positions, exons in the region and prior evidence of association with CAD please see S1 Table. We classified these 50 loci into 6 categories based on this prior evidence:

- 8 loci harbor SNPs which were found to be statistically associated with CAD in the CARDIoGRAMplusC4D study [26], the largest GWAS of CAD till date.
- 3 loci have significantly associated CARDIoGRAMplusC4D SNPs within ± 400 kb [26]
- 3 loci harbor SNPs which were found to be statistically associated with CAD in other GWAS.
- 11 loci have evidence of statistical association with risk factors of cardiovascular diseases (CVD), such as myocardial infarction, blood pressure, sudden cardiac arrest, heart failure, high-density lipoprotein cholesterol levels, triglyceride levels, etc.
- 3 loci are associated with obesity and related traits.
- We could not find any statistical association with CAD or its risk pathway for the remaining 22 loci.

**Fig 5:**
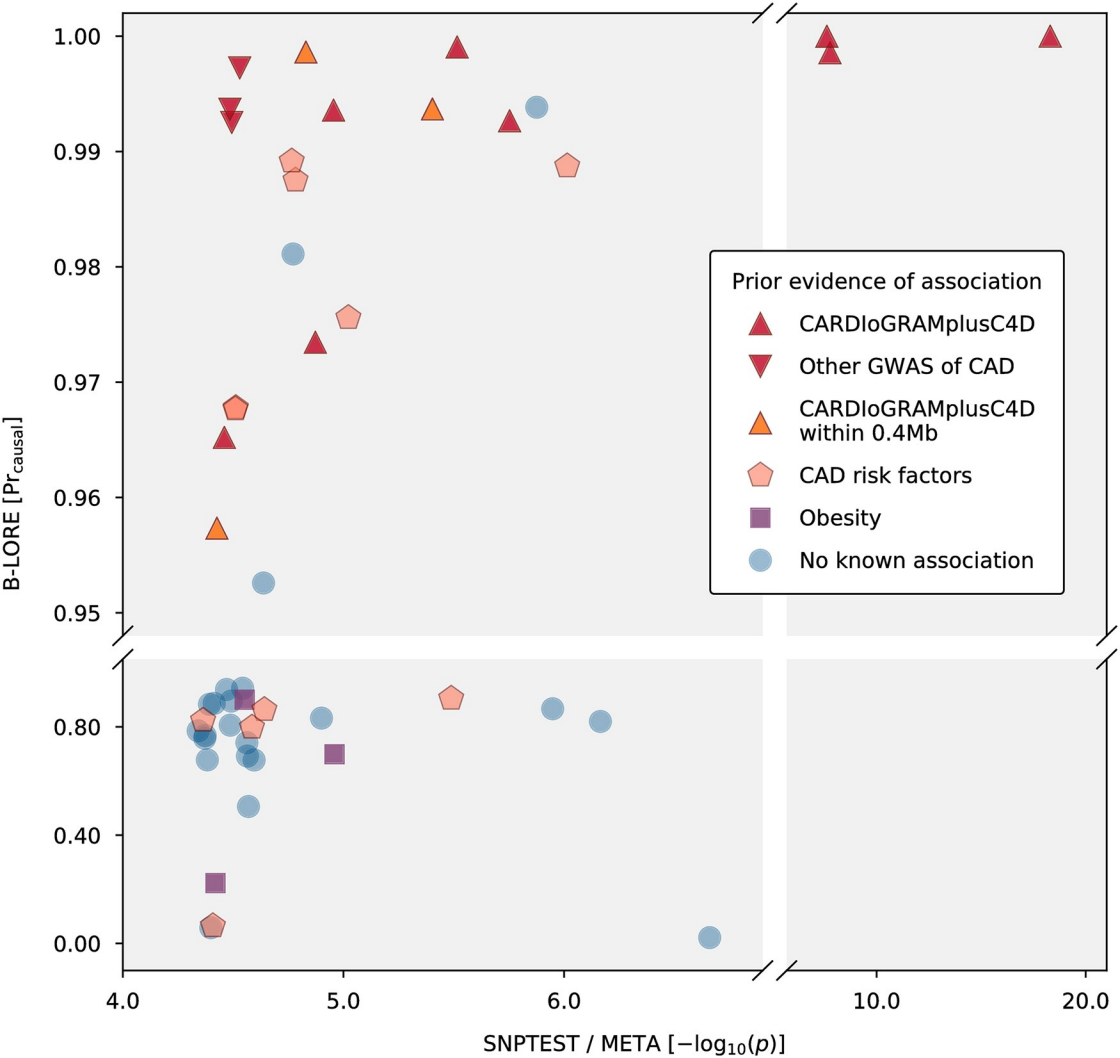
Association of genetic loci with CAD. Comparison of ranking of 50 genetic loci using meta-analysis across 5 cohorts (GerMIFS I-V [21–26]) with a total of 6234 cases and 6848 controls from white European ancestry. We first used meta-analysis of genome-wide SNPTEST summary statistics on these 5 small GWAS to select the top 50 loci and then applied B-LORE on these loci assuming a maximum of five causal SNPs per locus. On the *x*-axis of the scatter plot, we show the −log_10_(*p*) values obtained from META, and on the *y*-axis we show the probability of a locus being causal, obtained from B-LORE. The legend shows the classification of all the 50 CAD loci based on prior evidence of association in existing literature (see Real data analysis: GWAS for Coronary Artery Disease (CAD) and S1 Table). This literature-based classification gives a reasonable “ground truth” of causal and non-causal loci, despite our incomplete knowledge about true underlying association in reality.

We compared the ranking of these loci using the Pr_causal_ obtained from B-LORE and the −log_10_(*p*) obtained from META in Fig 5, We used the literature classification as qualitative indication for accuracy. Despite the modest sample size, B-LORE identified all the 11 CARDIoGRAMplusC4D loci (from the first 2 categories) present in the selected pool of loci with Pr_causal_ > 0.95. For comparison, SNPTEST / META could identify only 3 genome-wide significant loci. Additionally, B-LORE predicted 12 other novel loci with Pr_causal_ > 0.95. Three of them are significant hits in other CAD GWAS, and 6 of them are significantly associated with CVD risk-related phenotypes, such as blood pressure, high density lipoprotein cholesterol, triglyceride levels, etc. B-LORE also predicted low probabilities for many loci with no prior evidence of association despite being highly ranked by SNPTEST / META.

**Fig 6:**
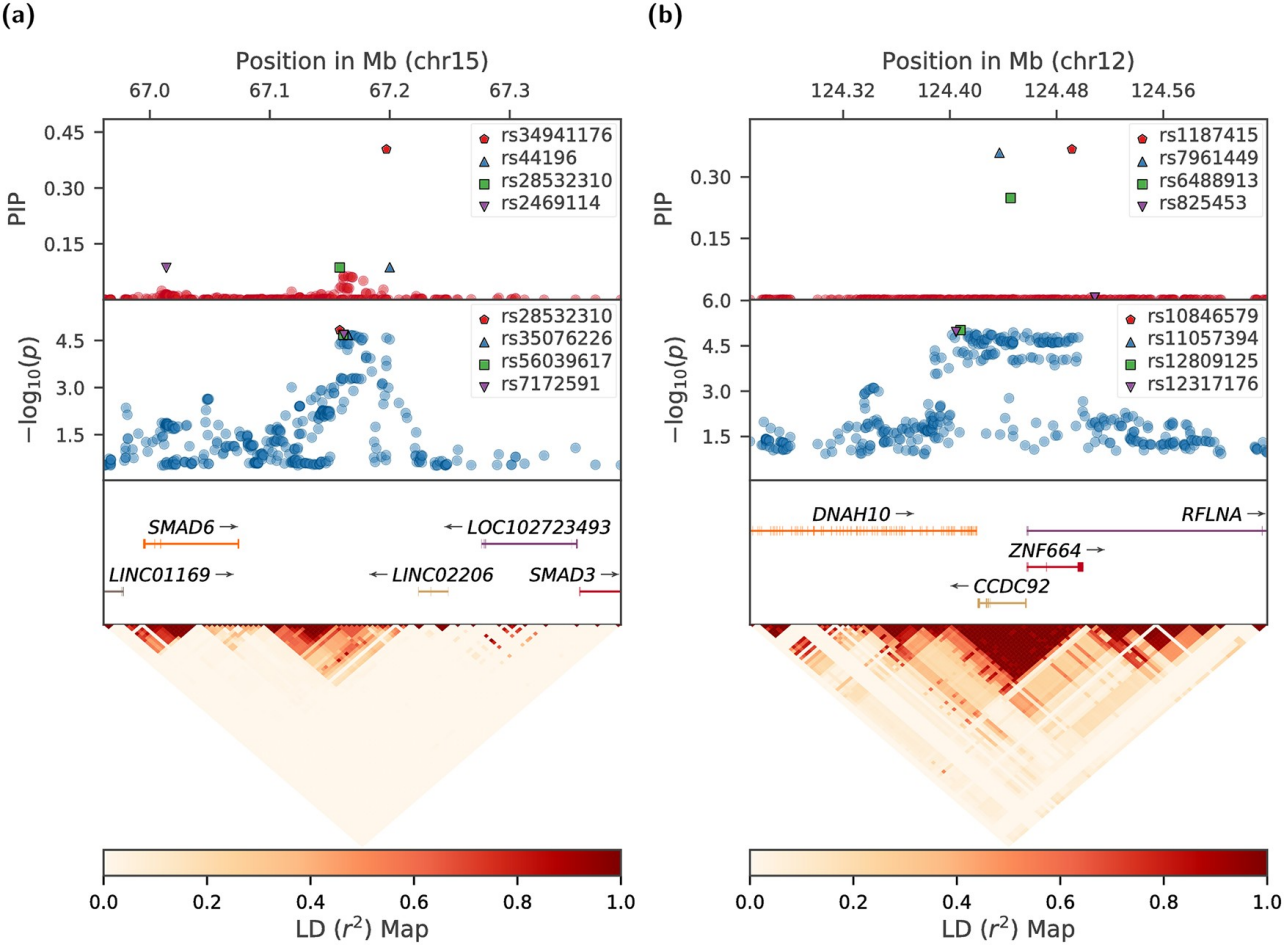
Representative examples of fine-mapping in CAD-associated loci. The top parts in A and B show the posterior inclusion probability (PIP) for each SNP to be causal as predicted by B-LORE. Below we plot the −log_10_(*p*) values for each SNP obtained from SNPTEST / META. The four best SNPs predicted by B-LORE and SNPTEST / META are marked by special symbols and annotated in the legends. At the bottom, we show the genes in the region and a heatmap describing the LD between the SNPs. (a): A known locus near SMAD3. (A SNP rs56062135 at 67.45Mb was found associated with CAD by the CARDIoGRAMplusC4D study [26]). The probability for finding at least one causal SNP in the locus is Pr_causal_ = 0.999. (b): A novel locus discovered by B-LORE, with Pr_causal_ = 0.976.

We show the fine-mapping performance of B-LORE at two example loci—a known risk locus near SMAD3 in chr15 (Fig 6A) and a novel risk locus at 12q24.31 (Fig 6B). At the SMAD3 locus, B-LORE picked up a single SNP (rs34941176) for explaining the association, while other SNPs from the region showed negligible probability of being associated. In the novel locus, there are three genes, DNAH10, ZNF664 and CCDC92, which are believed to be associated with multiple CAD risk factors such as high-density lipoprotein cholesterol level, triglycerides levels and waist-to-hip ratio [33–35] Here, B-LORE prioritized three SNPs (rs1187415, rs7961449 and rs6488913) in a region with strong LD. All SNPs in this region showed similarly significant *p*-values. In both the above cases, B-LORE prioritized SNPs which are different than the ones with lowest *p*-values in the region. In S10 Fig, we show the fine-mapping of all the 50 loci using B-LORE.

Indeed in most loci we find that B-LORE prioritizes a few SNPs, while SNPTEST / META predict similar *p*-values for many SNPs (S10 Fig). Due to lacking validation it is unclear so far whether the SNPs prioritized by B-LORE are actually causal.

## Discussion

Since its introduction in 2011, BVSR has remained the *de facto* method of choice for achieving the maximum power in GWAS multiple regression [5], but was limited by the requirement of individual genotype data. Several fine-mapping methods [9, 10, 36] introduced novel strategies to extend the scope of multiple regression analysis using only summary statistics from individual studies. However, none of these methods achieved more power [9, 36] than BVSR unless additional external information, such as ENCODE data or multiple traits, was used [6, 7, 37].

In the present work, we introduce B-LORE for performing multiple logistic regression on binary phenotypes, and show that it improves the power of case-control GWAS over both BVSR probit and linear models. In fact, the improvement achieved by B-LORE over BVSR is more significant than the improvement achieved by BVSR over single-SNP analyses (Fig 1). The key innovation is the quasi-Laplace approximation, which allows us to accurately compute posterior probabilities for SNP causality and to learn hyperparameters for the multiple logistic regression model.

Multiple logistic regression has received very little attention in GWAS. There were technical difficulties in the MCMC sampling of binary phenotypes for the BVSR probit model. The fine-mapping methods approximated the logistic likelihood with a Gaussian, which is essentially equivalent to using a scaled linear model. This linear approximation becomes inaccurate when moving out of the inflection point of the logistic function or when the spread of disease risk among patients is large.

We can get some intuition about this advantage of the logistic over the linear model from the partial derivatives of their log likelihoods with respect to the effect sizes, *β*,

$$\begin{array}{c} \frac{\partial }{\partial \beta_{i}}\log L(\beta )=\sum_{n=1}^{N}\left(\phi_{n}-p_{n}\right)\;x_{ni}\;. \end{array}$$ (20)

Here, *p*_*n*_ is given by Eq (1) for the logistic model and by *p*_*n*_ = ***β***^**⊤**^**x***_n_* for the linear one. The terms (*ϕ*_*n*_ − *p*_*n*_) can be interpreted as weights with which diseased patients (*ϕ* = 1) with minor alleles at SNP *i* (*x*_*ni*_ > 0) “vote” *β*_*i*_ up and healthy patients (*ϕ* = 0) with *x*_*ni*_ > 0 “vote” *β*_*i*_ down. The strongest contributions are made by healthy patients with high predicted disease risk *p*_*n*_ and by diseased patients with low predicted risk, while the smallest contributions are made by healthy patients with low predicted risk and diseased patients with high predicted risk. As long as *p*_*n*_ is near 0.5, the weights for both models are near 0.5 and they will give very similar results. But as predictability grows or the bias changes from 0.5 due to case/control ratios different from 1, the *p*_*n*_ for the logistic model will become quite different from those of the linear model and the linear model will become inaccurate. In extreme cases when ***β***^**⊤**^**x***_n_* gets smaller than 0 or larger than 1, patients will even “vote” with the wrong sign!

When applying logistic regression to a single SNP or when applying multiple logistic regression only on a single locus at a time, the predicted risk differences are small, the values of *p*_*n*_ lie very near to 0.5, and the linear approximation holds well. However, when predicted risks lie in the non-linear regime of the logistic curve, B-LORE outperforms—by a clear margin—methods based on a linear regression model and those based on a linear approximation of the logistic regression model. We gave two examples:

First, when applying multiple logistic regression to all risk loci, heritability adds up over the many loci, the predicted risks *p*_*n*_ scatter farther from 0.5 and the logistic model performs significantly better than the linear model (Fig 1, S7 Fig).

Second, when the case-control ratio *r* differs significantly from 1, the predicted risk of patients scatters around 1/(1+ *r*), which will differ significantly from 0.5. In this regime, B-LORE strongly outperformed the multiple linear regression methods (Fig 2). GWAS have been rarely analyzed with unbalanced case-control ratios. B-LORE could change that. It should be possible to discover many new loci and SNPs associated with complex diseases simply by using additional controls to existing GWAS, *e.g*. from one of the medical biobanks springing up across the world [38].

Third, predicting the disease risk from known covariates should significantly improve power of detecting causal SNPs, because the more accurate estimation of *p*_*n*_ would improve the weighting of patients. It is well known that age and sex alone can predict disease risk more accurately than genotype for most common diseases (*e.g*. cardiovascular diseases [39, 40]). Various other measurable covariates could further improve risk prediction. Using covariates to predict disease risk from external data sources [41] (and *not* from the case-control study itself, as this estimate would suffer from strong selection bias) could therefore greatly improve power in combination with our logistic multiple regression approach.

The flexibility of the Bayesian approach makes it easy to integrate this and other external information in the future, such as functional genomics data tracks on DNA accessibility, transcription factor binding etc. [6, 42–44] which can modulate the prior probability *π*_*i*_ for a SNP to be causal. We will explore more systematically how best to improve predictive performance of multiple logistic regression by adding these functional annotations.

The quasi-Laplace approximation also allows us to extend the analysis to multiple studies without using genotype data. Any method for GWAS meta-analysis that can improve the power has enormous leverage. It can be applied to pool millions of patients from multiple GWAS to help understand the origin of all common diseases in humans [2]. We hope that B-LORE will contribute to realizing this potential.

## Supporting information

S1 Text Additional methods. (PDF)

S1 Fig Distribution of the causal SNPs in our simulation. In each locus, we sampled the causal SNPs with a prior probability *π* and a constraint that at least one SNP must be picked. For our simulations, we choose *π* = 0.005. We show (a) the distribution of total number of causal SNPs used for each simulation, and (b) the distribution of causal SNPs in each locus averaged over all simulations.
(PDF)

S2 Fig Performance of B-LORE on null data. We performed a simulation with randomly generated binary phenotype on 13082 samples across five populations, using 17218 SNPs distributed over 100 loci. We show (a) the log posterior probability of each locus being causal (log_10_ (Pr_causal_)), and (b) the posterior inclusion probability (PIP) for each SNP.
(PDF)

S3 FigImbalance in case-control GWAS with fixed number of controls.We simulated phenotypes with varying case/control ratio—(a) 1625/6500, (b) 3250/6500, (c) 4875/6500 and (d) 6500/6500 respectively—using 100 loci of ∼200 SNPs, as described in the main text. All simulations used $h_{g}^{2}=0.4$. We compared the ranking of SNPs at each locus using recall (solid lines, left *y*-axis) and precision (dotted lines, right *y*-axis), which were averaged over 100 loci and 20 simulation replicates. All methods were run with a maximum of two causal SNPs per locus. Insets schematically compare logistic model with linear model (see Fig 1 for details). B-LORE shows increasingly more recall over other methods with increasing imbalance, because the logistic function becomes increasingly better than the linear function to model the data.(PDF)

S4 Fig Calibration of the posterior inclusion probabilities from B-LORE. The SNPs were put into 10 bins of width 0.1 according to their posterior inclusion probabilities (PIPs). Each point on the plot represents a single bin, with the center of the PIP within that bin on the *x*–axis and the proportion of SNPs which were true positives in that bin on the *y*–axis. Vertical bars show ±2 standard errors of the proportions, assuming a binomial distribution. Panel (a) is the result of B-LORE using maximum of 2 causal SNPs and panel (b) using maximum of 3 causal SNPs.
(PDF)

S5 Fig CPU time and memory requirement for calculating summary statistics with B-LORE. Panel (a) shows the processing time and panel (b) shows the maximum memory required for calculating B-LORE summary statistics with 50, 100, 150 and 200 loci. Each point corresponds to an average over 20 simulations, with the vertical bars representing ± standard errors. Different shapes and colors correspond to different sample sizes (*N* = 2000, 4000, 6000, 8000 and 10000) of GWA studies, as specified in the legend. The lines connect the results of the same sample size. Each locus contained 200 SNPs. All calculations were done on an Intel Xeon E5-2670 v2 processor with 8 cores.
(PDF)

S6 Fig CPU time and memory requirement for meta-analysis with B-LORE. We compared the computational requirements of B-LORE with other fine-mapping methods in terms of (a) processing time and (b) maximum memory required. Along the *x*–axis, we vary the number of maximum allowed causal SNPs. For each point on the plot, we used an average over 20 simulations. Each simulation was a meta-analysis of 5 GWA studies with 40000 SNPs (distributed over 200 loci). All calculations were done on an Intel Xeon E5-2670 v2 processor with 8 cores. FINEMAP and CAVIARBF were allowed to use all the cores in parallel, by analyzing 25 loci in each core.
(PDF)

S7 FigImpact of number of loci on calculation of B-LORE summary statistics.We simulated 13082 phenotypes using 100 loci of ∼200 SNPs, as described in the main text. All simulations used $h_{g}^{2}=0.6$. We then used only a subset (25, 50, 75 and 100) of these loci for further analysis, and the results are shown in panels (a), (b), (c) and (d) respectively. We compared the ranking of SNPs at each locus using recall (solid lines, left *y*-axis) and precision (dotted lines, right *y*-axis), which were averaged over the loci and the simulation replicates. All methods were run with a maximum of two causal SNPs per locus.(PDF)

S8 FigImpact of number of loci on optimization of hyperparameters for B-LORE meta-analysis.We simulated 13082 phenotypes using 100 loci of ∼200 SNPs, as described in the main text. All simulations used $h_{g}^{2}=0.6$. We calculated B-LORE summary statistics from all the 100 loci, but performed meta-analysis using only a subset (25, 50, 75 and 100—shown in panels (a), (b), (c) and (d) respectively) of these loci. Other methods were run on the same subset of loci. We compared the ranking of SNPs at each locus using recall (solid lines, left *y*-axis) and precision (dotted lines, right *y*-axis), which were averaged over the loci and the simulation replicates. All methods were run with a maximum of two causal SNPs per locus.(PDF)

S9 FigLow disease prevalence does not affect the performance of B-LORE.We show the performance of different methods using low disease prevalence and ascertained sampling with (a) case/control ratio of 1.0 and (b) case/control ratio of 0.25. We extracted the genotype (50 loci of ∼250 SNPs) of 100000 samples from the UK Biobank data. We simulated the phenotypes with a disease prevalence of 0.01. We selected all the cases (∼1000) and selected the required number of controls (given the case-control ratio) randomly from the remaining sample pool. Both simulations used $h_{g}^{2}=0.4$. We compared the ranking of SNPs at each locus using recall (solid lines, left *y*-axis) and precision (dotted lines, right *y*-axis), which were averaged over 50 loci and 20 simulation replicates. All methods were run with a maximum of two causal SNPs per locus. B-LORE shows more recall over other methods with addition of more controls because the logistic function becomes increasingly better at modeling the data.(PDF)

S10 Fig Fine-mapping of 50 genetic loci using B-LORE meta-analysis from 5 small GWAS (GerMIFS I-V). We first used meta-analysis of genome-wide SNPTEST summary statistics on these 5 small GWAS to select the top 50 loci and then applied B-LORE on these loci assuming a maximum of five causal SNPs per locus. Each plot has 4 panels from top to bottom: (1) The top panel shows the posterior inclusion probability (PIP) of each SNP obtained from B-LORE. (2) The second panel shows the −log_10_(*p*) values obtained from SNPTEST / META. (3) The third panel shows the genes present in the region. (4) The bottom panel shows the LD map between the SNPs of the region. The top 4 SNPs obtained from B-LORE and SNPTEST / META are highlighted and annotated in the legend of each plot. Below each plot, we mention the probability (Pr_causal_) of the locus containing at least one causal SNP.
(PDF)

S1 Table Association of genetic loci with CAD. Table of top 50 genetic loci used in the meta-analysis of CAD with the GerMIFS I–V GWA studies. Here, we summarize the location, literature classification and significance score for association of these loci with CAD. The columns are: ‘Chr’ is the chromosome number on which the locus is located, ‘Start’ is the starting base pair position of the locus on the chromosome, ‘End’ is the end base pair position of the locus on the chromosome, Pr_causal_ is the probability of the locus harboring a causal SNP as detected by B-LORE, −log_10_(*p*) is obtained from simple regression using SNPTEST/META, ‘Relevant genes’ are the list of genes which were found to be associated with CAD in prior studies (if no association is found then all genes are listed and if the locus is in a gene desert then no genes are listed), ‘Prior evidence’ gives a brief description of the prior study which showed CAD association.
(PDF)

## References

[1] Visscher PM, Wray NR, Zhang Q, Sklar P, McCarthy MI, Brown MA, et al 10 Years of GWAS Discovery: Biology, Function, and Translation. The American Journal of Human Genetics. 2017;101(1):5–22. 10.1016/j.ajhg.2017.06.005 28686856 PMC5501872

[2] MacArthur J, Bowler E, Cerezo M, Gil L, Hall P, Hastings E, et al The new NHGRI-EBI Catalog of published genome-wide association studies (GWAS Catalog). Nucleic Acids Research. 2017;45(D1):896–901. 10.1093/nar/gkw1133 PMC5210590 27899670

[3] Zhou X, Peter C, Matthew S. Polygenic modeling with Bayesian sparse linear mixed models. PLOS Genetics. 2013;9(2):1–14. 10.1371/journal.pgen.1003264 PMC3567190 23408905

[4] Servin B, Stephens M. Imputation-based analysis of association studies: Candidate regions and quantitative traits. PLOS Genetics. 2007;3(7):1–13. 10.1371/journal.pgen.0030114 PMC1934390 17676998

[5] Guan Y, Stephens M. Bayesian variable selection regression for genome-wide association studies and other large-scale problems. Annals of Applied Statistics. 2011;5(3):1780–1815. 10.1214/11-AOAS455

[6] Kichaev G, Wen-Yun Y, Sara L, Farhad H, Eleazar E, L PA, et al Integrating functional data to prioritize causal variants in statistical fine-mapping studies. PLOS Genetics. 2014;10(10):1–16. 10.1371/journal.pgen.1004722 PMC4214605 25357204

[7] Kichaev G, Roytman M, Johnson R, Eskin E, Lindström S, Kraft P, et al Improved methods for multi-trait fine mapping of pleiotropic risk loci. Bioinformatics. 2017;33(2):248–255. 10.1093/bioinformatics/btw615 27663501 PMC5254076

[8] Hormozdiari F, Kostem E, Kang EY, Pasaniuc B, Eskin E. Identifying causal variants at loci with multiple signals of association. Genetics. 2014;198(2):497–508. 10.1534/genetics.114.167908 25104515 PMC4196608

[9] Chen W, Larrabee BR, Ovsyannikova IG, Kennedy RB, Haralambieva IH, Poland GA, et al Fine mapping causal variants with an approximate Bayesian method using marginal test statistics. Genetics. 2015;200(3):719–736. 10.1534/genetics.115.176107 25948564 PMC4512539

[10] Benner C, Spencer CCA, Havulinna AS, Salomaa V, Ripatti S, Pirinen M. FINEMAP: Efficient variable selection using summary data from genome-wide association studies. Bioinformatics. 2016;32(10):1493–1501. 10.1093/bioinformatics/btw018 26773131 PMC4866522

[11] Schaid DJ, Chen W, Larson NB. From genome-wide associations to candidate causal variants by statistical fine-mapping. Nature Reviews Genetics. 2018;. 10.1038/s41576-018-0016-z 29844615 PMC6050137

[12] Newcombe PJ, Verzilli C, Casas JP, Hingorani AD, Smeeth L, Whittaker JC. Multilocus Bayesian Meta-Analysis of Gene-Disease Associations. The American Journal of Human Genetics. 2009;84(5):567–580. 10.1016/j.ajhg.2009.04.001 19409523 PMC2680997

[13] Cook JP, Mahajan A, Morris AP. Guidance for the utility of linear models in meta-analysis of genetic association studies of binary phenotypes. European Journal Of Human Genetics. 2016;25:240–245. 10.1038/ejhg.2016.150 27848946 PMC5237383

[14] Zhou X, Stephens M. Genome-wide efficient mixed-model analysis for association studies. Nature Genetics. 2012;44:821–824. 10.1038/ng.2310 22706312 PMC3386377

[15] Bishop CM. Pattern Recognition and Machine Learning. Secaucus, NJ, USA: Springer-Verlag New York, Inc.; 2006.

[16] Pirinen M, Donnelly P, Spencer CCA. Efficient computation with a linear mixed model on large-scale data sets with applications to genetic studies. Ann Appl Stat. 2013;7(1):369–390. 10.1214/12-AOAS586

[17] Marchini J, Howie B, Myers S, McVean G, Donnelly P. A new multipoint method for genome-wide association studies by imputation of genotypes. Nature Genetics. 2007;39(7):906–913. 10.1038/ng2088 17572673

[18] Consortium WTCC, et al Genome-wide association study of 14,000 cases of seven common diseases and 3,000 shared controls. Nature. 2007;447(7145):661–678. 10.1038/nature05911 17554300 PMC2719288

[19] Marchini J, Howie B. Genotype imputation for genome-wide association studies. Nature Review Genetics. 2010;11(7):499–511. 10.1038/nrg2796 20517342

[20] Newcombe PJ, Conti DV, Richardson S. JAM: A Scalable Bayesian Framework for Joint Analysis of Marginal SNP Effects. Genetic Epidemiology. 2016;40(3):188–201. 10.1002/gepi.21953 27027514 PMC4817278

[21] Samani NJ, Erdmann J, Hall AS, Hengstenberg C, Mangino M, Mayer B, et al Genomewide association analysis of coronary artery disease. New England Journal of Medicine. 2007;357(5):443–453. 10.1056/NEJMoa072366 17634449 PMC2719290

[22] Erdmann J, Groszhennig A, Braund PS, Konig IR, Hengstenberg C, Hall AS, et al New susceptibility locus for coronary artery disease on chromosome 3q22.3. Nature Genetics. 2009;41(3):280–282. 10.1038/ng.307 19198612 PMC2695543

[23] Schunkert H, Konig IR, Kathiresan S, Reilly MP, Assimes TL, Holm H, et al Large-scale association analysis identifies 13 new susceptibility loci for coronary artery disease. Nature Genetics. 2011;43(4):333–338. 10.1038/ng.784 21378990 PMC3119261

[24] Erdmann J, Willenborg C, Nahrstaedt J, Preuss M, König IR, Baumert J, et al Genome-wide association study identifies a new locus for coronary artery disease on chromosome 10p11.23. European Heart Journal. 2011;32(2):158–168. 10.1093/eurheartj/ehq405 21088011

[25] Deloukas P, Kanoni S, Willenborg C, Farrall M, Assimes TL, Thompson JR, et al Large-scale association analysis identifies new risk loci for coronary artery disease. Nature Genetics. 2013;45(1):25–33. 10.1038/ng.2480 23202125 PMC3679547

[26] CARDIoGRAMplusC4D. A comprehensive 1000 Genomes-based genome-wide association meta-analysis of coronary artery disease. Nature Genetics. 2015;47(10):1121–1130. 10.1038/ng.3396 26343387 PMC4589895

[27] Yang J, Lee SH, Goddard ME, Visscher PM. GCTA: A tool for genome-wide complex trait analysis. The American Journal of Human Genetics. 2011;88(1):76–82. 10.1016/j.ajhg.2010.11.011 21167468 PMC3014363

[28] Lee SH, Wray NR, Goddard ME, Visscher PM. Estimating missing heritability for disease from genome-wide association studies. The American Journal of Human Genetics. 2011;88(3):294–305. 10.1016/j.ajhg.2011.02.002 21376301 PMC3059431

[29] Benner C, Havulinna AS, Järvelin MR, Salomaa V, Ripatti S, Pirinen M. Prospects of Fine-Mapping Trait-Associated Genomic Regions by Using Summary Statistics from Genome-wide Association Studies. The American Journal of Human Genetics. 2017;101(4):539–551. 10.1016/j.ajhg.2017.08.012 28942963 PMC5630179

[30] Howson JMM, Zhao W, Barnes DR, Ho WK, Young R, Paul DS, et al Fifteen new risk loci for coronary artery disease highlight arterial-wall-specific mechanisms. Nature Genetics. 2017;49:1113–1119. 10.1038/ng.3874 28530674 PMC5555387

[31] Klarin D, Zhu QM, Emdin CA, Chaffin M, Horner S, McMillan BJ, et al Genetic analysis in UK Biobank links insulin resistance and transendothelial migration pathways to coronary artery disease. Nature Genetics. 2017;49:1392–1397. 10.1038/ng.3914 28714974 PMC5577383

[32] van der Harst P, Verweij N. Identification of 64 Novel Genetic Loci Provides an Expanded View on the Genetic Architecture of Coronary Artery Disease. Circulation Research. 2017;122:433–443. 10.1161/CIRCRESAHA.117.312086 29212778 PMC5805277

[33] Teslovich TM, Musunuru K, Smith AV, Edmondson AC, Stylianou IM, Koseki M, et al Biological, clinical and population relevance of 95 loci for blood lipids. Nature. 2010;466(7307):707–713. 10.1038/nature09270 20686565 PMC3039276

[34] Shungin D, Winkler TW, Croteau-Chonka DC, Ferreira T, Locke AE, Magi R, et al New genetic loci link adipose and insulin biology to body fat distribution. Nature. 2015;518(7538):187–196. 10.1038/nature14132 25673412 PMC4338562

[35] Dastani Z, Marie-France H, Nicholas T, B PJR, Xin Y, A SR, et al Novel loci for adiponectin levels and their influence on type 2 diabetes and metabolic traits: A multi-ethnic meta-analysis of 45,891 individuals. PLOS Genetics. 2012;8(3):1–23. 10.1371/journal.pgen.1002607 PMC3315470 22479202

[36] Zhu X, Stephens M. Bayesian large-scale multiple regression with summary statistics from genome-wide association studies. The Annals of Applied Statistics. 2017;11(3):1561–1592. 10.1214/17-AOAS1046 29399241 PMC5796536

[37] Li Y, Kellis M. Joint Bayesian inference of risk variants and tissue-specific epigenomic enrichments across multiple complex human diseases. Nucleic Acids Research. 2016;44(18):144 10.1093/nar/gkw627 PMC5062982 27407109

[38] Sudlow C, John G, Naomi A, Valerie B, Paul B, John D, et al UK Biobank: An Open Access Resource for Identifying the Causes of a Wide Range of Complex Diseases of Middle and Old Age. PLOS Medicine. 2015;12(3):1–10. 10.1371/journal.pmed.1001779 PMC4380465 25826379

[39] Yusuf S, Reddy S, Ôunpuu S, Anand S. Global Burden of Cardiovascular Diseases. Circulation. 2001;104(22):2746–2753. 10.1161/hc4601.099487 11723030

[40] Wilson PWF, D’Agostino RB, Levy D, Belanger AM, Silbershatz H, Kannel WB. Prediction of Coronary Heart Disease Using Risk Factor Categories. Circulation. 1998;97(18):1837–1847. 10.1161/01.CIR.97.18.1837 9603539

[41] Zaitlen N, Sara L, Bogdan P, Marilyn C, Giulio G, Samuela P, et al Informed Conditioning on Clinical Covariates Increases Power in Case-Control Association Studies. PLOS Genetics. 2012;8(11):1–13. 10.1371/journal.pgen.1003032 PMC3493452 23144628

[42] Chen W, McDonnell SK, Thibodeau SN, Tillmans LS, Schaid DJ. Incorporating Functional Annotations for Fine-Mapping Causal Variants in a Bayesian Framework Using Summary Statistics. Genetics. 2016;204(3):933–958. 10.1534/genetics.116.188953 27655946 PMC5105870

[43] Zhou J, Troyanskaya OG. Predicting effects of noncoding variants with deep learning-based sequence model. Nature Methods. 2015;12(10):931–934. 10.1038/nmeth.3547 26301843 PMC4768299

[44] Eraslan G, Arloth J, Martins J, Iurato S, Czamara D, Binder EB, et al. DeepWAS: Directly integrating regulatory information into GWAS using deep learning supports master regulator MEF2C as risk factor for major depressive disorder. bioRxiv. 2016;.

