# Supplementary material for: Bayesian multiple logistic regression for case-control GWAS
Source: PLoS Genet. 2018 Dec 31;14(12):e1007856. doi: 10.1371/journal.pgen.1007856 (PMC6329526; doi:10.1371/journal.pgen.1007856)
Supplement: S1 Text — (PDF) [file pgen.1007856.s001.pdf]

Bayesian multiple logistic regression for case-control GWAS – Supplemental Methods

Saikat Banerjee[1], Lingyao Zeng[2], Heribert Schunkert[2] and Johannes Söding[1,*]

[1]Max Planck Institute for Biophysical Chemistry, 37077 Göttingen, Germany

[2]German Heart Centre, 80636 Munich, Germany

[*]Corresponding author: soeding@mpibpc.mpg.de

1 Correction of covariates and population substructure

Covariates such as age, sex, lipid levels, etc. are treated as a separate hypothetical locus in B-LORE. For N patients with J covariates, we create a covariate matrix $\mathbf{X}' \in \mathbb{R}^{N\times J}$ and append it to the genotype matrix $\mathbf{X} \in \mathbb{R}^{N\times I}$ of I SNPs. The total data matrix after the concatenation has rows $\left(\mathbf{x}^{\mathsf{T}}, \mathbf{x}'^{\mathsf{T}}\right) \in \mathbb{R}^{I+J}$. Unlike the SNP loci, we do not use the point-normal prior for variable selection in the covariate locus, but use a normal prior. This is done by simply setting $\pi = 0$ and $\sigma = \sigma'$ for the covariate locus, and estimating σ' from the data (see below: section Factorization over loci). We calculate the posterior inclusion probability (PIP) for each covariate.

To correct for non-causal correlations of genotype with disease via population substructure, we perform a singular value decomposition (SVD) of the genotype matrix $\mathbf{X} = \sum_s \lambda_s \mathbf{u}_s^{\mathsf{T}} \boldsymbol{\beta}_s$, where λ_s are the singular values sorted in decreasing order, $\boldsymbol{\beta}_s \in \mathbb{R}^I$ the singular vectors or principal components and $\mathbf{u}_s \in \mathbb{R}^N$ are the *loadings* of the singular vectors. We store the loadings multiplied by the singular values of the first S (*e.g.* $S = 3 \ldots 10$) principal components as covariates, $\mathbf{x}'_n = (\lambda_1 u_{1,n}, \ldots, \lambda_S u_{S,n})^{\mathsf{T}}$ for each patient. The number of loadings used for the population substructure correction can be controlled by using the `--pca` option in B-LORE.

B-LORE uses pre-selected SNPs, and hence the PCA can only use these SNPs. A genome-wide PCA can also be performed by external tools and the principal components can be added as covariates.

2 Motivation of quasi-Laplace approximation

The quasi-Laplace approximation, as discussed in the main text, is a key concept in the methodology of B-LORE. We approximate the product of the likelihood of $\boldsymbol{\beta}$ and a *regularizer* with a Gaussian distribution.

$$p\left(\boldsymbol{\phi} \mid \mathbf{X}, \boldsymbol{\beta}\right) \mathcal{N}\left(\boldsymbol{\beta} \mid \mathbf{0}, \tilde{\sigma}^{\mathbf{2}}\mathbb{I}\right) \propto \mathcal{N}\left(\boldsymbol{\beta} \mid \tilde{\boldsymbol{\beta}}, \tilde{\boldsymbol{\Lambda}}^{-1}\right) . \tag{1}$$

The logarithm of the left-hand side, which we call regularized log likelihood,

$$\log \mathscr{L}_{\text{reg}}\left(\boldsymbol{\beta}\right) = \log p\left(\boldsymbol{\phi} \mid \mathbf{X}, \boldsymbol{\beta}\right) + \log \mathcal{N}\left(\boldsymbol{\beta} \mid \mathbf{0}, \tilde{\sigma}^{\mathbf{2}}\mathbb{I}\right) \tag{2}$$

is a quadratic function ($\log \mathcal{N}\left(\boldsymbol{\beta} \mid \mathbf{0}, \tilde{\sigma}^{\mathbf{2}}\mathbb{I}\right)$) with respect to any of the β_i, plus the sum of N concave functions. The approximation can be motivated from its validity in the limit of $N \gg 1$ and equal number of cases and controls. The Hessians of the concave functions must all have negative or zero diagonal elements and therefore their sum will grow roughly proportionally with the number of patients N. In contrast to the second derivatives, the third and higher partial derivatives will take both positive and negative signs. If the number of diseased and control patients is roughly equal, $p(\phi_n \mid \mathbf{x}_n, \boldsymbol{\beta})$

will mostly lie near $(1/N)\sum_n I(\phi_n = 1) \approx 0.5$, and therefore $\boldsymbol{\beta}^\mathsf{T}\mathbf{x}_n$ will be roughly as often positive as negative. Therefore the third partial derivatives will tend to be close to zero and have no preferred signs. The same is true of the higher derivatives. The magnitudes of the third and higher derivatives will grow only as $\sqrt{N}$ because their signs fluctuate around 0 for all patients. The second derivatives will increasingly dominate over the higher derivatives as N gets larger, and the log likelihood will be increasingly better approximated by a quadratic function, or in other words, by the logarithm of a multivariate Gaussian. As we see from the results, the above approximation could be extended to GWAS with strongly differing numbers of cases and controls.

3 Integration of the marginal likelihood

We optimize the hyperparameters by maximizing the marginal likelihood function, which is obtained by integrating over the effect sizes $\boldsymbol{\beta}$:

$$\begin{aligned}
\mathrm{m}\mathscr{L}(\pi,\sigma) &= p(\boldsymbol{\phi} \mid \mathbf{X}, \pi, \sigma) \\
&= \int p(\boldsymbol{\phi} \mid \boldsymbol{\beta}, \mathbf{X}, \pi, \sigma)\, p(\boldsymbol{\beta} \mid \pi, \sigma)\, d\boldsymbol{\beta} \\
&= \sum_{\mathbf{c}} p(\mathbf{c} \mid \pi, \sigma) \int p(\boldsymbol{\phi} \mid \mathbf{X}, \boldsymbol{\beta})\, \mathcal{N}\left(\boldsymbol{\beta} \mid \mathbf{0}, \mathrm{diag}\left(\boldsymbol{\sigma}_{\mathbf{c}}^{\mathbf{2}}\right)\right) d\boldsymbol{\beta} \\
&= \sum_{\mathbf{c}} p(\mathbf{c} \mid \pi, \sigma) \int p(\boldsymbol{\phi} \mid \mathbf{X}, \boldsymbol{\beta})\, \mathcal{N}\left(\boldsymbol{\beta} \mid \mathbf{0}, \tilde{\sigma}^{\mathbf{2}}\mathbb{I}\right) \frac{\mathcal{N}\left(\boldsymbol{\beta} \mid \mathbf{0}, \mathrm{diag}\left(\boldsymbol{\sigma}_{\mathbf{c}}^{\mathbf{2}}\right)\right)}{\mathcal{N}\left(\boldsymbol{\beta} \mid \mathbf{0}, \tilde{\sigma}^{\mathbf{2}}\mathbb{I}\right)} d\boldsymbol{\beta} \\
&\propto \sum_{\mathbf{c}} p(\mathbf{c} \mid \pi, \sigma) \int \mathcal{N}\left(\boldsymbol{\beta} \mid \tilde{\boldsymbol{\beta}}, \tilde{\boldsymbol{\Lambda}}^{-1}\right) \frac{\mathcal{N}\left(\boldsymbol{\beta} \mid \mathbf{0}, \mathrm{diag}\left(\boldsymbol{\sigma}_{\mathbf{c}}^{\mathbf{2}}\right)\right)}{\mathcal{N}\left(\boldsymbol{\beta} \mid \mathbf{0}, \tilde{\sigma}^{\mathbf{2}}\mathbb{I}\right)} d\boldsymbol{\beta} \qquad (3)
\end{aligned}$$

where we have used the quasi-Laplace approximation of Eq. (1) in the last step. We can analytically evaluate the integral in Eq. (3) because the logarithm of the integrand is now a quadratic function of $\boldsymbol{\beta}$. We make use of the following equality, which we prove in Appendix A:

$$\int \mathcal{N}\left(\boldsymbol{\beta} \mid \boldsymbol{\mu}_1, \boldsymbol{\Lambda}_1^{-1}\right) \frac{\mathcal{N}\left(\boldsymbol{\beta} \mid \boldsymbol{\mu}_2, \boldsymbol{\Lambda}_2^{-1}\right)}{\mathcal{N}\left(\boldsymbol{\beta} \mid \boldsymbol{\mu}_3, \boldsymbol{\Lambda}_3^{-1}\right)} d\boldsymbol{\beta} = \frac{\mathcal{N}\left(\mathbf{0} \mid \boldsymbol{\mu}_1, \boldsymbol{\Lambda}_1^{-1}\right) \mathcal{N}\left(\mathbf{0} \mid \boldsymbol{\mu}_2, \boldsymbol{\Lambda}_2^{-1}\right)}{\mathcal{N}\left(\mathbf{0} \mid \boldsymbol{\mu}_{123}, \boldsymbol{\Lambda}_{123}^{-1}\right) \mathcal{N}\left(\mathbf{0} \mid \boldsymbol{\mu}_3, \boldsymbol{\Lambda}_3^{-1}\right)} \qquad (4)$$

where

$$\boldsymbol{\Lambda}_{123} := \boldsymbol{\Lambda}_1 + \boldsymbol{\Lambda}_2 - \boldsymbol{\Lambda}_3 \qquad (5)$$

$$\boldsymbol{\mu}_{123} := \boldsymbol{\Lambda}_{123}^{-1}\left(\boldsymbol{\Lambda}_1\boldsymbol{\mu}_1 + \boldsymbol{\Lambda}_2\boldsymbol{\mu}_2 - \boldsymbol{\Lambda}_3\boldsymbol{\mu}_3\right) \qquad (6)$$

Identifying $\boldsymbol{\Lambda}_1 = \tilde{\boldsymbol{\Lambda}}$, $\boldsymbol{\Lambda}_2 = \mathrm{diag}\left(\boldsymbol{\sigma}_{\mathbf{c}}^2\right)^{-1}$, $\boldsymbol{\Lambda}_3 = \frac{1}{\tilde{\sigma}^2}\mathbb{I}$, $\boldsymbol{\mu}_1 = \tilde{\boldsymbol{\beta}}$, $\boldsymbol{\mu}_2 = \mathbf{0}$, $\boldsymbol{\mu}_3 = \mathbf{0}$ and defining

$$\boldsymbol{\Lambda}_{\mathbf{c}} := \tilde{\boldsymbol{\Lambda}} + \mathrm{diag}\left(\boldsymbol{\sigma}_{\mathbf{c}}^2\right)^{-1} - \frac{1}{\tilde{\sigma}^2}\mathbb{I} = \boldsymbol{\Lambda}_{123} \qquad (7)$$

$$\boldsymbol{\beta}_{\mathbf{c}} := \boldsymbol{\Lambda}_{\mathbf{c}}^{-1}\left(\tilde{\boldsymbol{\Lambda}}\tilde{\boldsymbol{\beta}}\right) = \boldsymbol{\mu}_{123} \qquad (8)$$

we obtain

$$\mathrm{m}\mathscr{L}(\pi,\sigma) \approx \sum_{\mathbf{c}} p(\mathbf{c} \mid \pi, \sigma) \frac{\mathcal{N}\left(\mathbf{0} \mid \tilde{\boldsymbol{\beta}}, \tilde{\boldsymbol{\Lambda}}^{\mathbf{-1}}\right) \mathcal{N}\left(\mathbf{0} \mid \mathbf{0}, \mathrm{diag}\left(\boldsymbol{\sigma}_{\mathbf{c}}^{\mathbf{2}}\right)\right)}{\mathcal{N}\left(\mathbf{0} \mid \boldsymbol{\beta}_{\mathbf{c}}, \boldsymbol{\Lambda}_{\mathbf{c}}^{-1}\right) \mathcal{N}\left(\mathbf{0} \mid \mathbf{0}, \tilde{\sigma}^{\mathbf{2}}\mathbb{I}\right)} \qquad (9)$$

The upper left and lower right Gaussians do not depend on π, σ or $\mathbf{c}$ and can be pulled into a constant factor D', yielding

$$\mathrm{m}\mathscr{L}(\pi,\sigma) = D' \sum_{\mathbf{c}} p(\mathbf{c} \mid \pi, \sigma) \frac{1}{|\boldsymbol{\Lambda}_{\mathbf{c}}|^{\frac{1}{2}} \left|\mathrm{diag}\left(\boldsymbol{\sigma}_{\mathbf{c}}^{2}\right)\right|^{\frac{1}{2}}} \exp\left(\frac{1}{2}\boldsymbol{\beta}_{\mathbf{c}}^{\mathsf{T}}\boldsymbol{\Lambda}_{\mathbf{c}}\boldsymbol{\beta}_{\mathbf{c}}\right) \qquad (10)$$

4 Estimation of $\tilde{\sigma}$

We use an iterative estimation procedure to find optimum regularization parameter $\tilde{\sigma}$. For the quasi-Laplace approximation to hold well, we need to find $\tilde{\sigma}$ that make the regularizer almost as good a prior as the full two-component mixtures prior. This can be achieved by optimizing (with respect to $\tilde{\sigma}$) a simplified marginal likelihood in which the regularizer replaces the full prior.

We start by setting $\tilde{\sigma} = 0.01$. We then find improved estimates of $\tilde{\sigma}$ by setting $\tilde{\sigma} := \sigma$ (note equivalence to $\tilde{\sigma}^2\mathbb{I} = \mathrm{diag}\left(\boldsymbol{\sigma}_{\mathbf{c}}^2\right)$), which corresponds to only one single causality configuration $\mathbf{c} = \{1, 1, \cdots, 1\}$ in which all SNPs are causal – and maximising the likelihood in Eq. (10) with respect to σ. We iterate a few times reesitmating improved values for σ every time using the new, updated estimates of $\tilde{\sigma}$.

5 Calculation of summary statistics

As discussed in the main text, we intend to learn the mode $\tilde{\boldsymbol{\beta}}$ and precision $\tilde{\boldsymbol{\Lambda}}$ of the regularized likelihood given by Eq. (1). Given $\tilde{\sigma}$, we maximize the regularized log likelihood of Eq. (2) with respect to $\boldsymbol{\beta}$ using the gradient-based optimisation method L-BFGS. The partial derivatives are:

$$\frac{\partial}{\partial v_i} \log \mathscr{L}_{\mathrm{reg}}\left(\boldsymbol{\beta}\right) = \sum_{n=1}^{N} \left(\phi_n - p_n\right) x_{ni} - \frac{\beta_i}{\tilde{\sigma}^2} \tag{11}$$

where $p_n = p(\phi_n = 1 \mid \mathbf{x}_n, \boldsymbol{\beta})$. The solution of this optimisation, $\tilde{\boldsymbol{\beta}}$, is the mean and mode of the Gaussian. The term $-\beta_i/\tilde{\sigma}^2$ pulls β_i towards zero. It prevents the maximum likelihood solutions β_i^* to assume large values in the absence of strong evidence for a SNP-disease coupling, *e.g.* when two SNPs are in near-perfect linkage desequilibrium and therefore very highly anti-correlated.

To find the precision matrix, we note that it should be equal to the negative Hessian matrix $-\mathbf{H}$ of the regularized log likelihood at $\tilde{\boldsymbol{\beta}}$. Using the derivative of the logistic function $\mathrm{lf}(x) = 1/(1 + e^{-x})$ given by,

$$\frac{d\,\mathrm{lf}(x)}{dx} = \mathrm{lf}(x)(1 - \mathrm{lf}(x)), \tag{12}$$

we obtain the matrix elements of the Hessian $\mathbf{H}$,

$$\begin{aligned} \frac{\partial^2}{\partial v_i \partial v_j} \log \mathscr{L}_{\mathrm{reg}}\left(\tilde{\boldsymbol{\beta}}\right) &= -\sum_{n=1}^{N} p\left(\phi_n = 1 \mid \mathbf{x}_n, \tilde{\boldsymbol{\beta}}\right) p\left(\phi_n = 0 \mid \mathbf{x}_n, \tilde{\boldsymbol{\beta}}\right) x_{ni} x_{nj} - \frac{\delta_{ij}}{\tilde{\sigma}^2} \\ &= -\sum_{n=1}^{N} \tilde{p}_n \left(1 - \tilde{p}_n\right) x_{ni} x_{nj} - \frac{\delta_{ij}}{\tilde{\sigma}^2} \end{aligned} \tag{13}$$

where $\tilde{p}_n = p\left(\phi_n = 1 \mid \mathbf{x}_n, \tilde{\boldsymbol{\beta}}\right)$. The equation shows that strongly correlated SNPs will have high coupling coefficients in the Hessian matrix. The precision matrix of the Gaussian distribution in our quasi-Laplace approximation is therefore,

$$\tilde{\boldsymbol{\Lambda}} = \sum_{n=1}^{N} \tilde{p}_n \left(1 - \tilde{p}_n\right) \mathbf{x}_n \mathbf{x}_n^{\mathsf{T}} + \mathrm{diag}\left(\frac{1}{\tilde{\sigma}^2}\right) \tag{14}$$

which is the sum of two matrices, one proportional to a weighted sample covariance matrix of the $x_1, \ldots, x_I$ and the precision matrix of the regularization prior. For comparison, the covariance matrix from LD is $\boldsymbol{\Lambda}_{\mathrm{LD}} = \sum_{n=1}^{N} \mathbf{x}_n \mathbf{x}_n^{\mathsf{T}}$

6 Factorization over loci

If the covariance matrix of the genotype $\mathbf{X}^\mathsf{T}\mathbf{X}$ is block-diagonal, then it is obvious from Eq. (14) that $\tilde{\boldsymbol{\Lambda}}$ is also block-diagonal. This allows us to factorise the marginal likelihood in equation Eq. (10), writing $\mathbf{c}_l$ for the binary configuration vector corresponding to the I_l SNPs of locus l and $\boldsymbol{\sigma}_{\mathbf{c},l}$, $\boldsymbol{\Lambda}_{\mathbf{c},l}$ and $\boldsymbol{\beta}_{\mathbf{c},l}$ for the subvectors and submatrices corresponding to only the SNPs of locus l:

$$\mathrm{m}\mathscr{L}\left(\pi,\sigma,\sigma'\right) = D' \sum_{\mathbf{c}} \prod_{l=1}^{L+1} p\left(\mathbf{c}_l \mid \pi,\sigma\right) \frac{\exp\left(\frac{1}{2}\boldsymbol{\beta}_{\mathbf{c},l}^\mathsf{T}\boldsymbol{\Lambda}_{\mathbf{c},l}\boldsymbol{\beta}_{\mathbf{c},l}\right)}{\left|\boldsymbol{\Lambda}_{\mathbf{c},l}\right|^{\frac{1}{2}} \left|\mathrm{diag}\left(\boldsymbol{\sigma}_{\mathbf{c},l}^2\right)\right|^{\frac{1}{2}}} \tag{15}$$

For notational brevity, we defined a virtual locus $l = L+1$ for the confounding variables, $\mathbf{x}_{L+1} = \mathbf{x}'$. We allow only one configuration $\mathbf{c}_{L+1} := \{1, 1, \cdots, 1\}$ so that $\|\mathbf{c}_{L+1}\| := J$ and define $p\left(\mathbf{c}_{L+1} \mid \pi,\sigma\right) := 1$ and $\boldsymbol{\sigma}_{\mathbf{c},L+1} := \sigma'\mathbb{I}$.

Finally, we denote all possible configurations in locus l as $\mathbf{c}_l$, which allows us to write

$$\mathrm{m}\mathscr{L}\left(\pi,\sigma,\sigma'\right) = p\left(\boldsymbol{\phi} \mid \mathbf{X},\pi,\sigma,\sigma'\right) = D' \prod_{l=1}^{L+1} \sum_{\mathbf{c}_l} p\left(\mathbf{c}_l \mid \pi,\sigma,\sigma'\right) F_l\left(\mathbf{c}_l,\pi,\sigma,\sigma'\right) \tag{16}$$

with

$$F_l\left(\mathbf{c}_l,\pi,\sigma,\sigma'\right) = \frac{\exp\left(\frac{1}{2}\boldsymbol{\beta}_{\mathbf{c},l}^\mathsf{T}\boldsymbol{\Lambda}_{\mathbf{c},l}\boldsymbol{\beta}_{\mathbf{c},l}\right)}{\left|\boldsymbol{\Lambda}_{\mathbf{c},l}\right|^{\frac{1}{2}} \left|\mathrm{diag}\left(\boldsymbol{\sigma}_{\mathbf{c},l}^2\right)\right|^{\frac{1}{2}}} \tag{17}$$

7 Branch and bound algorithm to restrict the sum over c to non-negligible terms

The sums over $\mathbf{c}_l$ in Eq. (16) run over 2^{I_l} terms. In the following we propose a method that omits terms in the sum over $\mathbf{c}_l$ that do not stand a chance of contributing significantly to it. We denote the total number of significant configurations chosen for locus l as $\mathcal{C}_l$. For any given configuration $\mathbf{c}_l$, the number of causal SNPs is simply the norm of the z-state, given by $\|\mathbf{c}_l\| = k$. We progressively increase the allowed number of causal SNPs k upto an allowed maximum of $k_{\max}$. At every step of $\|\mathbf{c}_l\| = k$, the total number of possible configurations is given by $\mathcal{C}_k$, out of which we select only significant configurations $\mathcal{C}'_k$ to be appended to $\mathcal{C}_l$.

We define the unnormalised posterior probabilities as

$$\tilde{p}\left(\mathbf{c}_l \mid \boldsymbol{\phi},\mathbf{X},\pi,\sigma\right) := p\left(\mathbf{c}_l \mid \pi,\sigma\right) F_l\left(\mathbf{c}_l,\pi,\sigma\right) \tag{18}$$

The algorithm is initialized with $k = 0$ and $k = 1$:

$$\begin{aligned} S_0 &= \tilde{p}\left(\mathbf{c}_l = \mathbf{0} \mid \boldsymbol{\phi},\mathbf{X},\pi,\sigma\right) \\ S_1 &= \sum_{\|\mathbf{c}_l\|=1} \tilde{p}\left(\mathbf{c}_l \mid \boldsymbol{\phi},\mathbf{X},\pi,\sigma\right) \\ \mathcal{C}_1 &= \mathcal{C}'_1 = \left\{\mathbf{c} \in \{0,1\}^I : \|\mathbf{c}_l\| = 1\right\} \end{aligned} \tag{19}$$

At every step with $k > 1$ we use all the possible configurations $\mathcal{C}_k$ to compute

$$S_k = \sum_{\mathbf{c}_l \in \mathcal{C}_k} \tilde{p}\left(\mathbf{c}_l \mid \boldsymbol{\phi},\mathbf{X},\pi,\sigma\right) \tag{20}$$

To determine the significant configurations $\mathcal{C}'_k$, we sort all the $\mathcal{C}_k$ configurations by

decreasing posterior probability and keep appending to $\mathcal{C}'_k$ until

$$\sum_{\mathbf{c}_l \in \mathcal{C}'_k} \tilde{p}\left(\mathbf{c}_l \mid \boldsymbol{\phi}, \mathbf{X}, \pi, \sigma\right) \geq 0.98 S_k \tag{21}$$

We combine these $\mathcal{C}'_k$ configurations with all the $I_l - k$ SNPs into new $\mathcal{C}_{k+1}$ configurations. We again select $\mathcal{C}'_{k+1}$ configurations out of all the available $\mathcal{C}_{k+1}$ configurations, and this continues iteratively. We stop the iteration when $S_{k+1} < 0.02 S_k$, because the terms with even higher $\|\mathbf{c}_l\|$ will not contribute significantly to the total sum over all configurations. The posterior probabilities are then approximated as

$$p\left(\mathbf{c}_l \mid \boldsymbol{\phi}, \mathbf{X}, \pi, \sigma\right) = \frac{\tilde{p}\left(\mathbf{c}_l \mid \boldsymbol{\phi}, \mathbf{X}, \pi, \sigma\right)}{\sum\limits_{k=0}^{k_{\max}} S_k} \tag{22}$$

Since risk loci will usually only contain a few causal SNPs, this procedure should normally stop without needing to compute millions of terms.

Appendix A

We show that the following result holds for any three D-dimensional Gaussian distributions $\mathcal{N}\left(\boldsymbol{\beta} \mid \boldsymbol{\mu}_1, \boldsymbol{\Lambda}_1^{-1}\right)$, $\mathcal{N}\left(\boldsymbol{\beta} \mid \boldsymbol{\mu}_2, \boldsymbol{\Lambda}_2^{-1}\right)$, and $\mathcal{N}\left(\boldsymbol{\beta} \mid \boldsymbol{\mu}_3, \boldsymbol{\Lambda}_3^{-1}\right)$,

$$\int \mathcal{N}\left(\boldsymbol{\beta} \mid \boldsymbol{\mu}_1, \boldsymbol{\Lambda}_1^{-1}\right) \frac{\mathcal{N}\left(\boldsymbol{\beta} \mid \boldsymbol{\mu}_2, \boldsymbol{\Lambda}_2^{-1}\right)}{\mathcal{N}\left(\boldsymbol{\beta} \mid \boldsymbol{\mu}_3, \boldsymbol{\Lambda}_3^{-1}\right)} d\boldsymbol{\beta} = \frac{\mathcal{N}\left(\mathbf{0} \mid \boldsymbol{\mu}_1, \boldsymbol{\Lambda}_1^{-1}\right) \mathcal{N}\left(\mathbf{0} \mid \boldsymbol{\mu}_2, \boldsymbol{\Lambda}_2^{-1}\right)}{\mathcal{N}\left(\mathbf{0} \mid \boldsymbol{\mu}_{123}, \boldsymbol{\Lambda}_{123}^{-1}\right) \mathcal{N}\left(\mathbf{0} \mid \boldsymbol{\mu}_3, \boldsymbol{\Lambda}_3^{-1}\right)} \tag{23}$$

where

$$\boldsymbol{\Lambda}_{123} := \boldsymbol{\Lambda}_1 + \boldsymbol{\Lambda}_2 - \boldsymbol{\Lambda}_3 \tag{24}$$

$$\boldsymbol{\mu}_{123} := \boldsymbol{\Lambda}_{123}^{-1}\left(\boldsymbol{\Lambda}_1\boldsymbol{\mu}_1 + \boldsymbol{\Lambda}_2\boldsymbol{\mu}_2 - \boldsymbol{\Lambda}_3\boldsymbol{\mu}_3\right) \tag{25}$$

We start by writing out the Gaussian functions explicitly:

$$\int \mathcal{N}\left(\boldsymbol{\beta} \mid \boldsymbol{\mu}_1, \boldsymbol{\Lambda}_1^{-1}\right) \frac{\mathcal{N}\left(\boldsymbol{\beta} \mid \boldsymbol{\mu}_2, \boldsymbol{\Lambda}_2^{-1}\right)}{\mathcal{N}\left(\boldsymbol{\beta} \mid \boldsymbol{\mu}_3, \boldsymbol{\Lambda}_3^{-1}\right)} d\boldsymbol{\beta} = \frac{|\boldsymbol{\Lambda}_1|^{\frac{1}{2}} |\boldsymbol{\Lambda}_2|^{\frac{1}{2}}}{|\boldsymbol{\Lambda}_3|^{\frac{1}{2}} (2\pi)^{\frac{D}{2}}}$$
$$\times \int \exp\left(-\frac{1}{2}\left[\left(\boldsymbol{\beta} - \boldsymbol{\mu}_1\right)^{\mathsf{T}} \boldsymbol{\Lambda}_1 \left(\boldsymbol{\beta} - \boldsymbol{\mu}_1\right) + \left(\boldsymbol{\beta} - \boldsymbol{\mu}_2\right)^{\mathsf{T}} \boldsymbol{\Lambda}_2 \left(\boldsymbol{\beta} - \boldsymbol{\mu}_2\right) - \left(\boldsymbol{\beta} - \boldsymbol{\mu}_3\right)^{\mathsf{T}} \boldsymbol{\Lambda}_3 \left(\boldsymbol{\beta} - \boldsymbol{\mu}_3\right)\right]\right) d\boldsymbol{\beta} \tag{26}$$

To perform the integral, we need to write the terms within the exp function as a Gaussian. For that purpose, we need to sort them into quadratic, linear and constant terms in $\boldsymbol{\beta}$,

$$\begin{aligned}
&\left(\boldsymbol{\beta} - \boldsymbol{\mu}_1\right)^{\mathsf{T}} \boldsymbol{\Lambda}_1 \left(\boldsymbol{\beta} - \boldsymbol{\mu}_1\right) + \left(\boldsymbol{\beta} - \boldsymbol{\mu}_2\right)^{\mathsf{T}} \boldsymbol{\Lambda}_2 \left(\boldsymbol{\beta} - \boldsymbol{\mu}_2\right) - \left(\boldsymbol{\beta} - \boldsymbol{\mu}_3\right)^{\mathsf{T}} \boldsymbol{\Lambda}_3 \left(\boldsymbol{\beta} - \boldsymbol{\mu}_3\right) \\
&\quad = \boldsymbol{\beta}^{\mathsf{T}} \left(\boldsymbol{\Lambda}_1 + \boldsymbol{\Lambda}_2 - \boldsymbol{\Lambda}_3\right) \boldsymbol{\beta} - 2\left(\boldsymbol{\mu}_1^{\mathsf{T}}\boldsymbol{\Lambda}_1 + \boldsymbol{\mu}_2^{\mathsf{T}}\boldsymbol{\Lambda}_2 - \boldsymbol{\mu}_3^{\mathsf{T}}\boldsymbol{\Lambda}_3\right)\boldsymbol{\beta} + \boldsymbol{\mu}_1^{\mathsf{T}}\boldsymbol{\Lambda}_1\boldsymbol{\mu}_1 + \boldsymbol{\mu}_2^{\mathsf{T}}\boldsymbol{\Lambda}_2\boldsymbol{\mu}_2 - \boldsymbol{\mu}_3^{\mathsf{T}}\boldsymbol{\Lambda}_3\boldsymbol{\mu}_3 \\
&\quad = \boldsymbol{\beta}^{\mathsf{T}}\boldsymbol{\Lambda}_{123}\boldsymbol{\beta} - 2\boldsymbol{\mu}_{123}^{\mathsf{T}}\boldsymbol{\Lambda}_{123}\boldsymbol{\beta} + \boldsymbol{\mu}_1^{\mathsf{T}}\boldsymbol{\Lambda}_1\boldsymbol{\mu}_1 + \boldsymbol{\mu}_2^{\mathsf{T}}\boldsymbol{\Lambda}_2\boldsymbol{\mu}_2 - \boldsymbol{\mu}_3^{\mathsf{T}}\boldsymbol{\Lambda}_3\boldsymbol{\mu}_3 \\
&\quad = \left(\boldsymbol{\beta}^{\mathsf{T}} - \boldsymbol{\mu}_{123}^{\mathsf{T}}\right)\boldsymbol{\Lambda}_{123}\left(\boldsymbol{\beta} - \boldsymbol{\mu}_{123}\right) - \boldsymbol{\mu}_{123}^{\mathsf{T}}\boldsymbol{\Lambda}_{123}\boldsymbol{\mu}_{123} + \boldsymbol{\mu}_1^{\mathsf{T}}\boldsymbol{\Lambda}_1\boldsymbol{\mu}_1 + \boldsymbol{\mu}_2^{\mathsf{T}}\boldsymbol{\Lambda}_2\boldsymbol{\mu}_2 - \boldsymbol{\mu}_3^{\mathsf{T}}\boldsymbol{\Lambda}_3\boldsymbol{\mu}_3
\end{aligned}$$

We can insert this expression into Eq. (26) and perform the integration over the Gaussian function,

$$
\begin{aligned}
&\int \mathcal{N}\left(\boldsymbol{\beta} \mid \boldsymbol{\mu}_1, \boldsymbol{\Lambda}_1^{-1}\right) \frac{\mathcal{N}\left(\boldsymbol{\beta} \mid \boldsymbol{\mu}_2, \boldsymbol{\Lambda}_2^{-1}\right)}{\mathcal{N}\left(\boldsymbol{\beta} \mid \boldsymbol{\mu}_3, \boldsymbol{\Lambda}_3^{-1}\right)} d\boldsymbol{\beta} \\
&= \frac{|\boldsymbol{\Lambda}_1|^{\frac{1}{2}} |\boldsymbol{\Lambda}_2|^{\frac{1}{2}}}{|\boldsymbol{\Lambda}_3|^{\frac{1}{2}} (2\pi)^{\frac{D}{2}}} \times \int \exp\left(-\frac{1}{2}\left(\boldsymbol{\beta}^\mathsf{T} - \boldsymbol{\mu}_{123}^\mathsf{T}\right) \boldsymbol{\Lambda}_{123} \left(\boldsymbol{\beta} - \boldsymbol{\mu}_{123}\right)\right) d\boldsymbol{\beta} \\
&\qquad \times \exp\left(-\frac{1}{2}\left[\boldsymbol{\mu}_1^\mathsf{T}\boldsymbol{\Lambda}_1\boldsymbol{\mu}_1 + \boldsymbol{\mu}_2^\mathsf{T}\boldsymbol{\Lambda}_2\boldsymbol{\mu}_2 - \boldsymbol{\mu}_3^\mathsf{T}\boldsymbol{\Lambda}_3\boldsymbol{\mu}_3 - \boldsymbol{\mu}_{123}^\mathsf{T}\boldsymbol{\Lambda}_{123}\boldsymbol{\mu}_{123}\right]\right) \\
&= \left(\frac{|\boldsymbol{\Lambda}_1| \, |\boldsymbol{\Lambda}_2|}{|\boldsymbol{\Lambda}_{123}| \, |\boldsymbol{\Lambda}_3|}\right)^{\frac{1}{2}} \exp\left(-\frac{1}{2}\left[\boldsymbol{\mu}_1^\mathsf{T}\boldsymbol{\Lambda}_1\boldsymbol{\mu}_1 + \boldsymbol{\mu}_2^\mathsf{T}\boldsymbol{\Lambda}_2\boldsymbol{\mu}_2 - \boldsymbol{\mu}_3^\mathsf{T}\boldsymbol{\Lambda}_3\boldsymbol{\mu}_3 - \boldsymbol{\mu}_{123}^\mathsf{T}\boldsymbol{\Lambda}_{123}\boldsymbol{\mu}_{123}\right]\right) \\
&= \frac{\mathcal{N}\left(\mathbf{0} \mid \boldsymbol{\mu}_1, \boldsymbol{\Lambda}_1^{-1}\right) \mathcal{N}\left(\mathbf{0} \mid \boldsymbol{\mu}_2, \boldsymbol{\Lambda}_2^{-1}\right)}{\mathcal{N}\left(\mathbf{0} \mid \boldsymbol{\mu}_{123}, \boldsymbol{\Lambda}_{123}^{-1}\right) \mathcal{N}\left(\mathbf{0} \mid \boldsymbol{\mu}_3, \boldsymbol{\Lambda}_3^{-1}\right)}
\end{aligned} \tag{27}
$$

This proves the proposition in Eq. (23).
