# Supplementary material for: Bayesian multiple logistic regression for case-control GWAS
Source: PLoS Genet. 2018 Dec 31;14(12):e1007856. doi: 10.1371/journal.pgen.1007856 (PMC6329526; doi:10.1371/journal.pgen.1007856)
Supplement: S1 Fig — In each locus, we sampled the causal SNPs with a prior probability π and a constraint that at least one SNP must be picked. For our simulations, we choose π = 0.005. We show (a) the distribution of total number of causal SNPs used for each simulation, and (b) the distribution of causal SNPs in each locus averaged over all simulations. (PDF) [file pgen.1007856.s002.pdf]

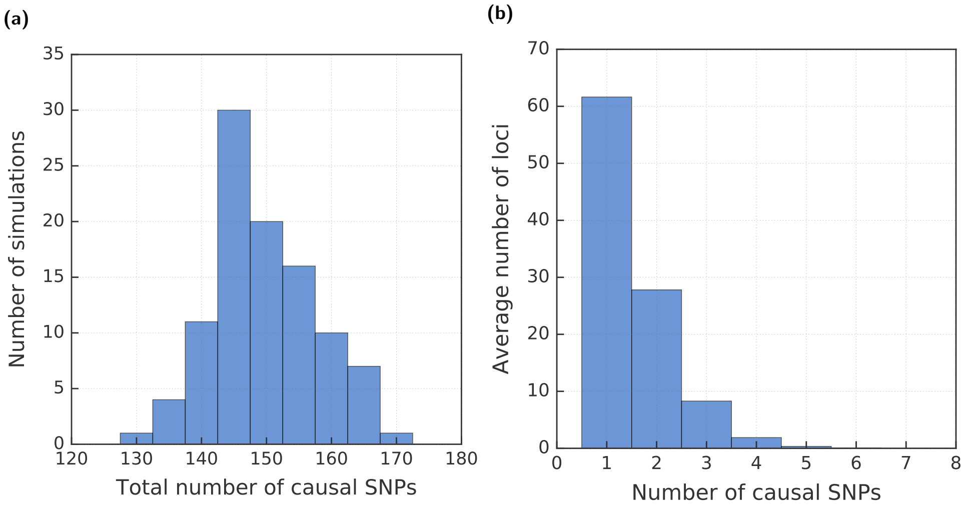

Figure S1. Distribution of the causal SNPs in our simulation. In each locus, we sampled the causal SNPs with a prior probability π and a constraint that at least one SNP must be picked. For our simulations, we choose $\pi = 0.005$. We show (a) the distribution of total number of causal SNPs used for each simulation, and (b) the distribution of causal SNPs in each locus averaged over all simulations.
