# Supplementary material for: Bayesian multiple logistic regression for case-control GWAS
Source: PLoS Genet. 2018 Dec 31;14(12):e1007856. doi: 10.1371/journal.pgen.1007856 (PMC6329526; doi:10.1371/journal.pgen.1007856)
Supplement: S2 Fig — We performed a simulation with randomly generated binary phenotype on 13082 samples across five populations, using 17218 SNPs distributed over 100 loci. We show (a) the log posterior probability of each locus being causal (log10 (Prcausal)), and (b) the posterior inclusion probability (PIP) for each SNP. (PDF) [file pgen.1007856.s003.pdf]

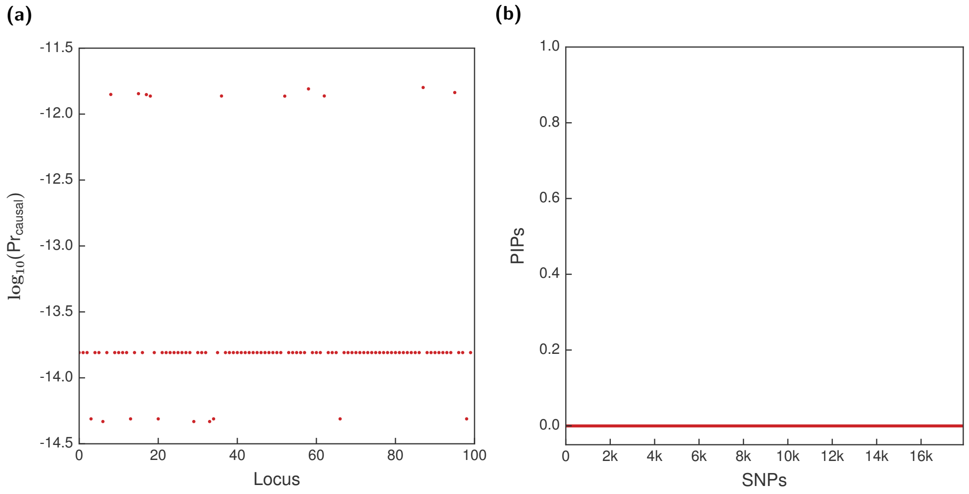

Figure S2. Performance of B-LORE on null data. We performed a simulation with randomly generated binary phenotype on 13082 samples across five populations, using 17218 SNPs distributed over 100 loci. We show (a) the log posterior probability of each locus being causal ($\log_{10}(\mathrm{Pr}_{\mathrm{causal}})$), and (b) the posterior inclusion probability (PIP) for each SNP.
