# Supplementary material for: Bayesian multiple logistic regression for case-control GWAS
Source: PLoS Genet. 2018 Dec 31;14(12):e1007856. doi: 10.1371/journal.pgen.1007856 (PMC6329526; doi:10.1371/journal.pgen.1007856)
Supplement: S3 Fig — We simulated phenotypes with varying case/control ratio—(a) 1625/6500, (b) 3250/6500, (c) 4875/6500 and (d) 6500/6500 respectively—using 100 loci of ∼200 SNPs, as described in the main text. All simulations used hg2=0.4. We compared the ranking of SNPs at each locus using recall (solid lines, left y-axis) and precision (dotted lines, right y-axis), which were averaged over 100 loci and 20 simulation replicates. All methods were run with a maximum of two causal SNPs per locus. Insets schematically compare logistic model with linear model (see Fig 1 for details). B-LORE shows increasingly more recall over other methods with increasing imbalance, because the logistic function becomes increasingly better than the linear function to model the data. (PDF) [file pgen.1007856.s004.pdf]

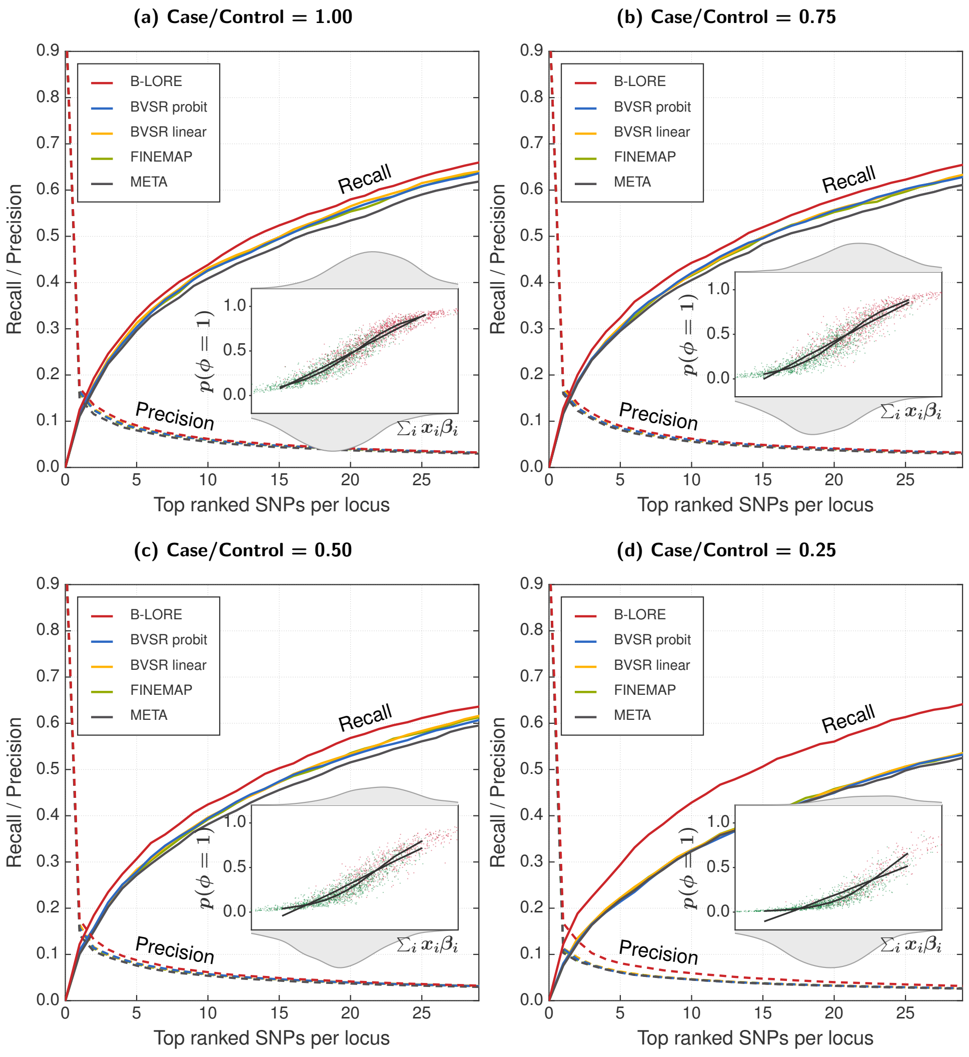

Figure S3. Imbalance in case-control GWAS with fixed number of controls. We simulated phenotypes with varying case/control ratio – (a) 6500/6500, (b) 4875/6500, (c) 3250/6500 and (d) 1625/6500 respectively – using 100 loci of ~200 SNPs, as described in the main text. All simulations used $h_g^2 = 0.4$. We compared the ranking of SNPs at each locus using recall (solid lines, left y-axis) and precision (dotted lines, right y-axis), which were averaged over 100 loci and 20 simulation replicates. All methods were run with a maximum of two causal SNPs per locus. Insets schematically compare logistic model with linear model. B-LORE shows increasingly more recall over other methods with increasing imbalance, because the logistic function becomes increasingly better than the linear function to model the data.
