# Supplementary material for: Bayesian multiple logistic regression for case-control GWAS
Source: PLoS Genet. 2018 Dec 31;14(12):e1007856. doi: 10.1371/journal.pgen.1007856 (PMC6329526; doi:10.1371/journal.pgen.1007856)
Supplement: S4 Fig — The SNPs were put into 10 bins of width 0.1 according to their posterior inclusion probabilities (PIPs). Each point on the plot represents a single bin, with the center of the PIP within that bin on the x–axis and the proportion of SNPs which were true positives in that bin on the y–axis. Vertical bars show ±2 standard errors of the proportions, assuming a binomial distribution. Panel (a) is the result of B-LORE using maximum of 2 causal SNPs and panel (b) using maximum of 3 causal SNPs. (PDF) [file pgen.1007856.s005.pdf]

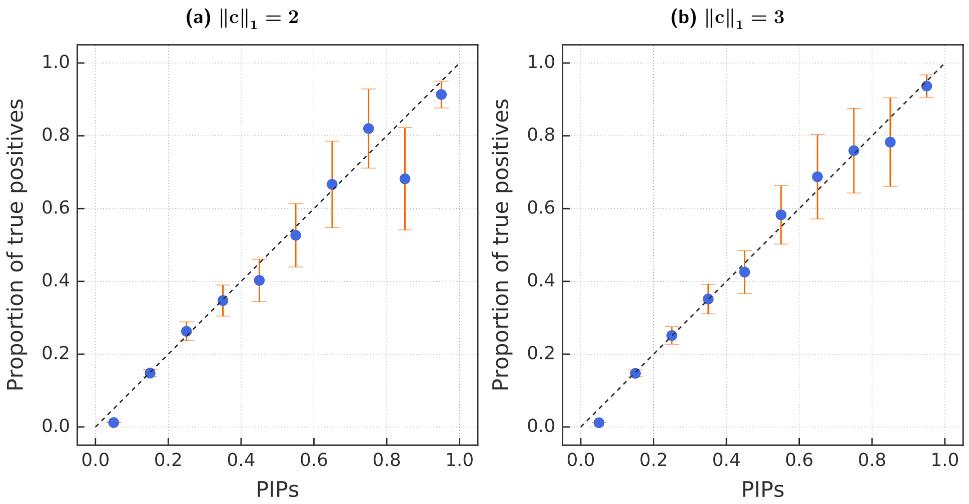

Figure S4. Calibration of the posterior inclusion probabilities from B-LORE. The SNPs were put into 10 bins of width 0.1 according to their posterior inclusion probabilities (PIPs). Each point on the plot represents a single bin, with the center of the PIP within that bin on the $x-$axis and the proportion of SNPs which were true positives in that bin on the $y-$axis. Vertical bars show ± 2 standard errors of the proportions, assuming a binomial distribution. Panel (a) is the result of B-LORE using maximum of 2 causal SNPs and panel (b) using maximum of 3 causal SNPs.
