# Supplementary material for: Bayesian multiple logistic regression for case-control GWAS
Source: PLoS Genet. 2018 Dec 31;14(12):e1007856. doi: 10.1371/journal.pgen.1007856 (PMC6329526; doi:10.1371/journal.pgen.1007856)
Supplement: S5 Fig — Panel (a) shows the processing time and panel (b) shows the maximum memory required for calculating B-LORE summary statistics with 50, 100, 150 and 200 loci. Each point corresponds to an average over 20 simulations, with the vertical bars representing ± standard errors. Different shapes and colors correspond to different sample sizes (N = 2000, 4000, 6000, 8000 and 10000) of GWA studies, as specified in the legend. The lines connect the results of the same sample size. Each locus contained 200 SNPs. All calculations were done on an Intel Xeon E5-2670 v2 processor with 8 cores. (PDF) [file pgen.1007856.s006.pdf]

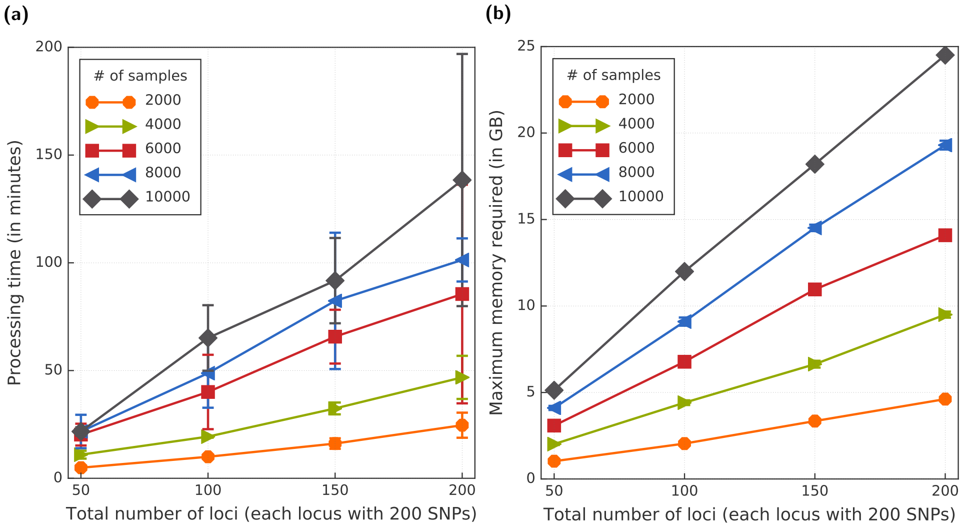

Figure S5. CPU time and memory requirement for calculating summary statistics with B-LORE. Panel (a) shows the processing time and panel (b) shows the maximum memory required for calculating B-LORE summary statistics with 50, 100, 150 and 200 loci. Each point corresponds to an average over 20 simulations, with the vertical bars representing ± standard errors. Different shapes and colors correspond to different sample sizes (N = 2000, 4000, 6000, 8000 and 10000) of GWA studies, as specified in the legend. The lines connect the results of the same sample size. Each locus contained 200 SNPs. All calculations were done on an Intel Xeon E5-2670 v2 processor with 8 cores.
