# Supplementary material for: Bayesian multiple logistic regression for case-control GWAS
Source: PLoS Genet. 2018 Dec 31;14(12):e1007856. doi: 10.1371/journal.pgen.1007856 (PMC6329526; doi:10.1371/journal.pgen.1007856)
Supplement: S6 Fig — We compared the computational requirements of B-LORE with other fine-mapping methods in terms of (a) processing time and (b) maximum memory required. Along the x–axis, we vary the number of maximum allowed causal SNPs. For each point on the plot, we used an average over 20 simulations. Each simulation was a meta-analysis of 5 GWA studies with 40000 SNPs (distributed over 200 loci). All calculations were done on an Intel Xeon E5-2670 v2 processor with 8 cores. FINEMAP and CAVIARBF were allowed to use all the cores in parallel, by analyzing 25 loci in each core. (PDF) [file pgen.1007856.s007.pdf]

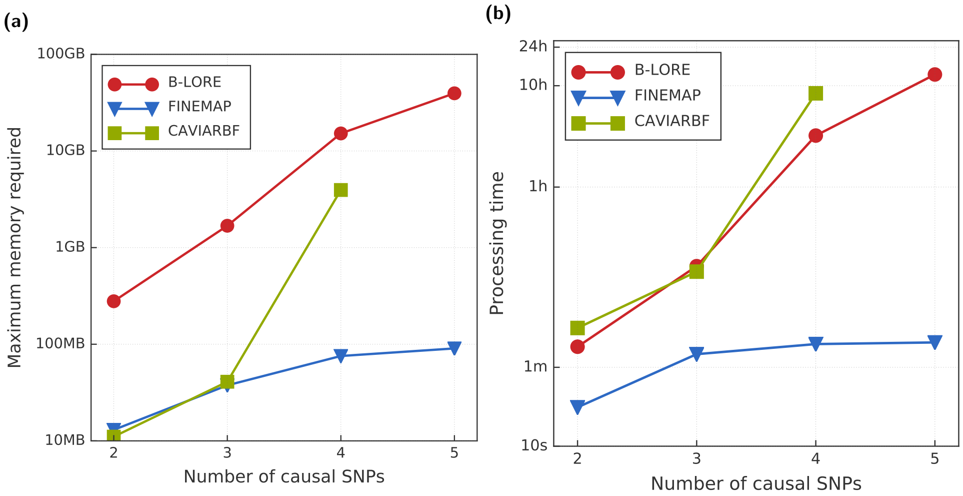

Figure S6. CPU time and memory requirement for meta-analysis with B-LORE. We compared the computational requirements of B-LORE with other finemapping methods in terms of (a) processing time and (b) maximum memory required. Along the $x-$axis, we vary the number of maximum allowed causal SNPs. For each point on the plot, we used an average over 20 simulations. Each simulation was a meta-analysis of 5 GWA studies with 40000 SNPs (distributed over 200 loci). All calculations were done on an Intel Xeon E5-2670 v2 processor with 8 cores. FINEMAP and CAVIARBF were allowed to use all the cores in parallel, by analyzing 25 loci in each core.
