# Supplementary material for: Bayesian multiple logistic regression for case-control GWAS
Source: PLoS Genet. 2018 Dec 31;14(12):e1007856. doi: 10.1371/journal.pgen.1007856 (PMC6329526; doi:10.1371/journal.pgen.1007856)
Supplement: S7 Fig — We simulated 13082 phenotypes using 100 loci of ∼200 SNPs, as described in the main text. All simulations used hg2=0.6. We then used only a subset (25, 50, 75 and 100) of these loci for further analysis, and the results are shown in panels (a), (b), (c) and (d) respectively. We compared the ranking of SNPs at each locus using recall (solid lines, left y-axis) and precision (dotted lines, right y-axis), which were averaged over the loci and the simulation replicates. All methods were run with a maximum of two causal SNPs per locus. (PDF) [file pgen.1007856.s008.pdf]

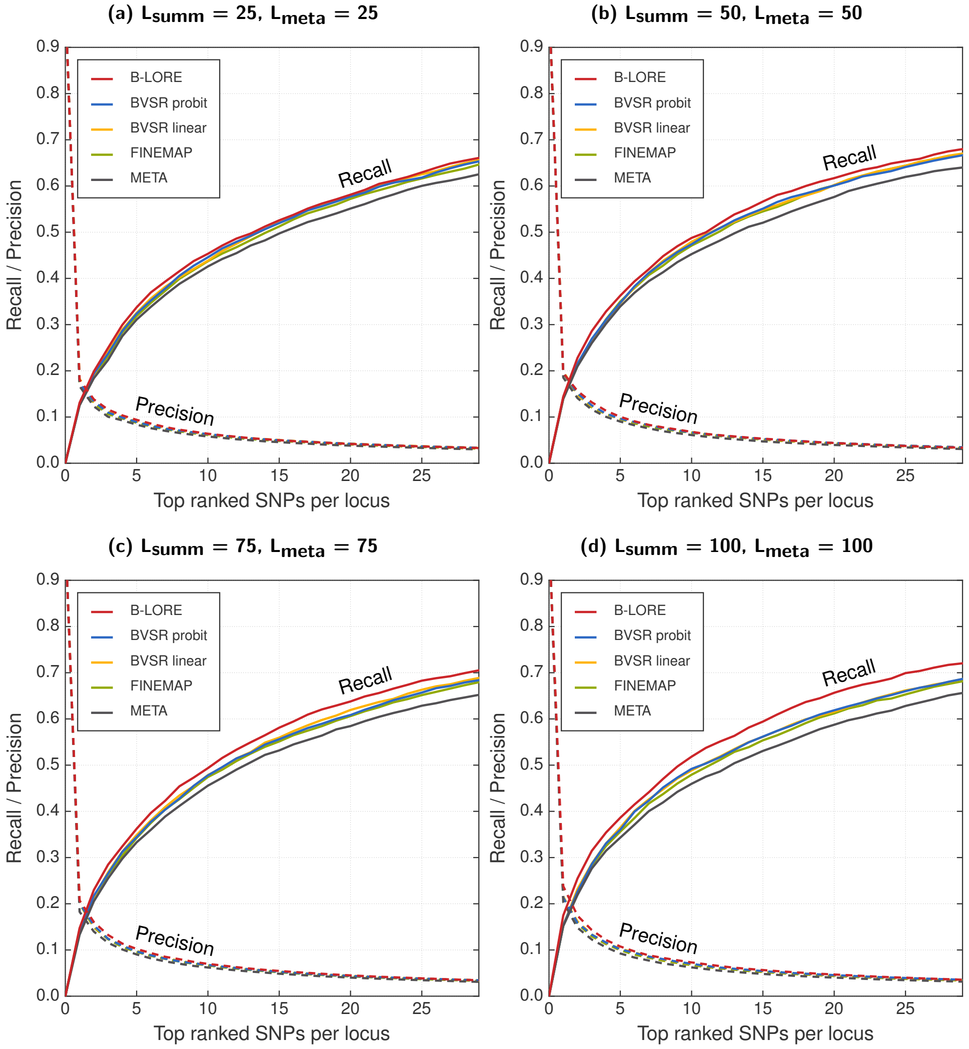


**Figure S7. Impact of number of loci on calculation of B-LORE summary statistics.** We simulated 13082 phenotypes using 100 loci of ∼200 SNPs, as described in the main text. All simulations used $h_g^2 = 0.6$. We then used only a subset (25, 50, 75 and 100) of these loci for further analysis, and the results are shown in panels (a), (b), (c) and (d) respectively. Here, $L_{summ}$ denotes the number of loci used for calculating summary statistics and $L_{meta}$ denotes the number of loci used for metaanalysis. We compared the ranking of SNPs at each locus using recall (solid lines, left $y$-axis) and precision (dotted lines, right $y$-axis), which were averaged over the loci and the simulation replicates. All methods were run with a maximum of two causal SNPs per locus.
