# Supplementary material for: Bayesian multiple logistic regression for case-control GWAS
Source: PLoS Genet. 2018 Dec 31;14(12):e1007856. doi: 10.1371/journal.pgen.1007856 (PMC6329526; doi:10.1371/journal.pgen.1007856)
Supplement: S8 Fig — We simulated 13082 phenotypes using 100 loci of ∼200 SNPs, as described in the main text. All simulations used hg2=0.6. We calculated B-LORE summary statistics from all the 100 loci, but performed meta-analysis using only a subset (25, 50, 75 and 100—shown in panels (a), (b), (c) and (d) respectively) of these loci. Other methods were run on the same subset of loci. We compared the ranking of SNPs at each locus using recall (solid lines, left y-axis) and precision (dotted lines, right y-axis), which were averaged over the loci and the simulation replicates. All methods were run with a maximum of two causal SNPs per locus. (PDF) [file pgen.1007856.s009.pdf]

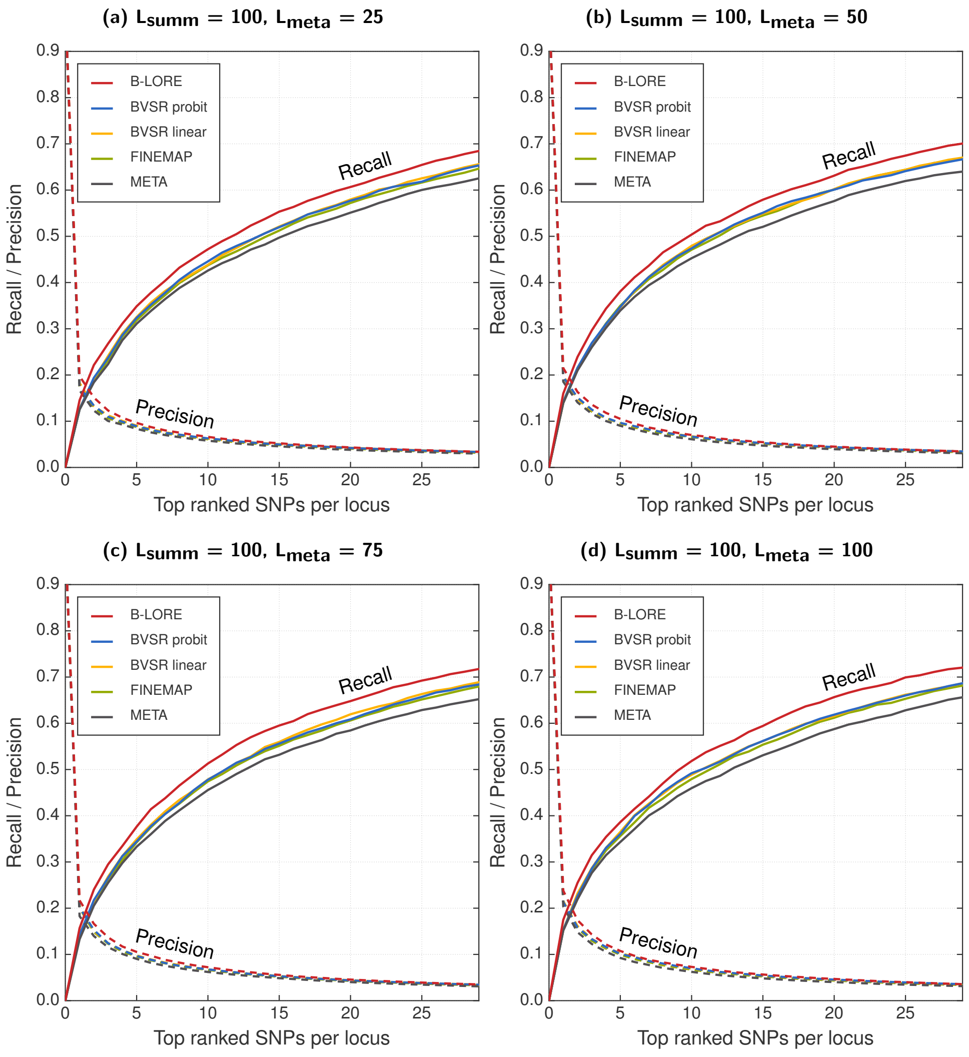

Figure S8. Impact of number of loci on optimization of hyperparameters for B-LORE metaanalysis. We simulated 13082 phenotypes using 100 loci of ~200 SNPs, as described in the main text. All simulations used $h_g^2 = 0.6$. We calculated B-LORE summary statistics from all the 100 loci, but performed metaanalysis using only a subset (25, 50, 75 and 100 – shown in panels (a), (b), (c) and (d) respectively) of these loci. Here, L_{summ} denotes the number of loci used for calculating summary statistics and L_{meta} denotes the number of loci used for metaanalysis. Other methods also used the same subset of loci. We compared the ranking of SNPs at each locus using recall (solid lines, left y-axis) and precision (dotted lines, right y-axis), which were averaged over the loci and the simulation replicates. All methods were run with a maximum of two causal SNPs per locus.
