# Supplementary material for: Bayesian multiple logistic regression for case-control GWAS
Source: PLoS Genet. 2018 Dec 31;14(12):e1007856. doi: 10.1371/journal.pgen.1007856 (PMC6329526; doi:10.1371/journal.pgen.1007856)
Supplement: S9 Fig — We show the performance of different methods using low disease prevalence and ascertained sampling with (a) case/control ratio of 1.0 and (b) case/control ratio of 0.25. We extracted the genotype (50 loci of ∼250 SNPs) of 100000 samples from the UK Biobank data. We simulated the phenotypes with a disease prevalence of 0.01. We selected all the cases (∼1000) and selected the required number of controls (given the case-control ratio) randomly from the remaining sample pool. Both simulations used hg2=0.4. We compared the ranking of SNPs at each locus using recall (solid lines, left y-axis) and precision (dotted lines, right y-axis), which were averaged over 50 loci and 20 simulation replicates. All methods were run with a maximum of two causal SNPs per locus. B-LORE shows more recall over other methods with addition of more controls because the logistic function becomes increasingly better at modeling the data. (PDF) [file pgen.1007856.s010.pdf]

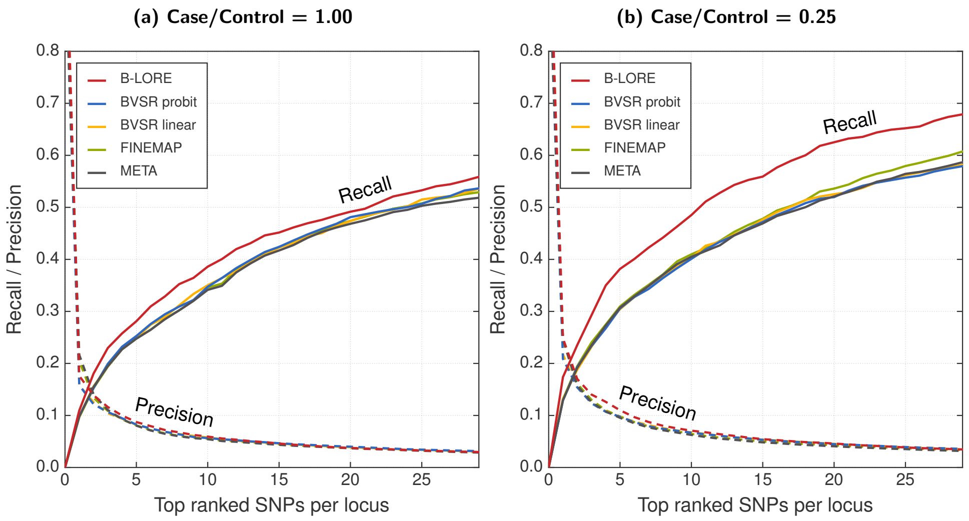

Figure S9. Low disease prevalence does not affect the performance of B-LORE. We show the performance of different methods using low disease prevalence and ascertained sampling with (a) case/control ratio of 1.0 and (b) case/control ratio of 0.25. We extracted the genotype (50 loci of ~250 SNPs) of 100000 samples from the UK Biobank data. We simulated the phenotypes with a disease prevalence of 0.01. We selected all the cases (~1000) and selected the required number of controls (given the case-control ratio) randomly from the remaining sample pool. Both simulations used $h_g^2 = 0.4$. We compared the ranking of SNPs at each locus using recall (solid lines, left y-axis) and precision (dotted lines, right y-axis), which were averaged over 50 loci and 20 simulation replicates. All methods were run with a maximum of two causal SNPs per locus. B-LORE shows more recall over other methods with addition of more controls because the logistic function becomes increasingly better at modeling the data.
