# Supplementary material for: Bayesian multiple logistic regression for case-control GWAS
Source: PLoS Genet. 2018 Dec 31;14(12):e1007856. doi: 10.1371/journal.pgen.1007856 (PMC6329526; doi:10.1371/journal.pgen.1007856)
Supplement: S10 Fig — We first used meta-analysis of genome-wide SNPTEST summary statistics on these 5 small GWAS to select the top 50 loci and then applied B-LORE on these loci assuming a maximum of five causal SNPs per locus. Each plot has 4 panels from top to bottom: (1) The top panel shows the posterior inclusion probability (PIP) of each SNP obtained from B-LORE. (2) The second panel shows the −log10(p) values obtained from SNPTEST / META. (3) The third panel shows the genes present in the region. (4) The bottom panel shows the LD map between the SNPs of the region. The top 4 SNPs obtained from B-LORE and SNPTEST / META are highlighted and annotated in the legend of each plot. Below each plot, we mention the probability (Prcausal) of the locus containing at least one causal SNP. (PDF) [file pgen.1007856.s011.pdf]

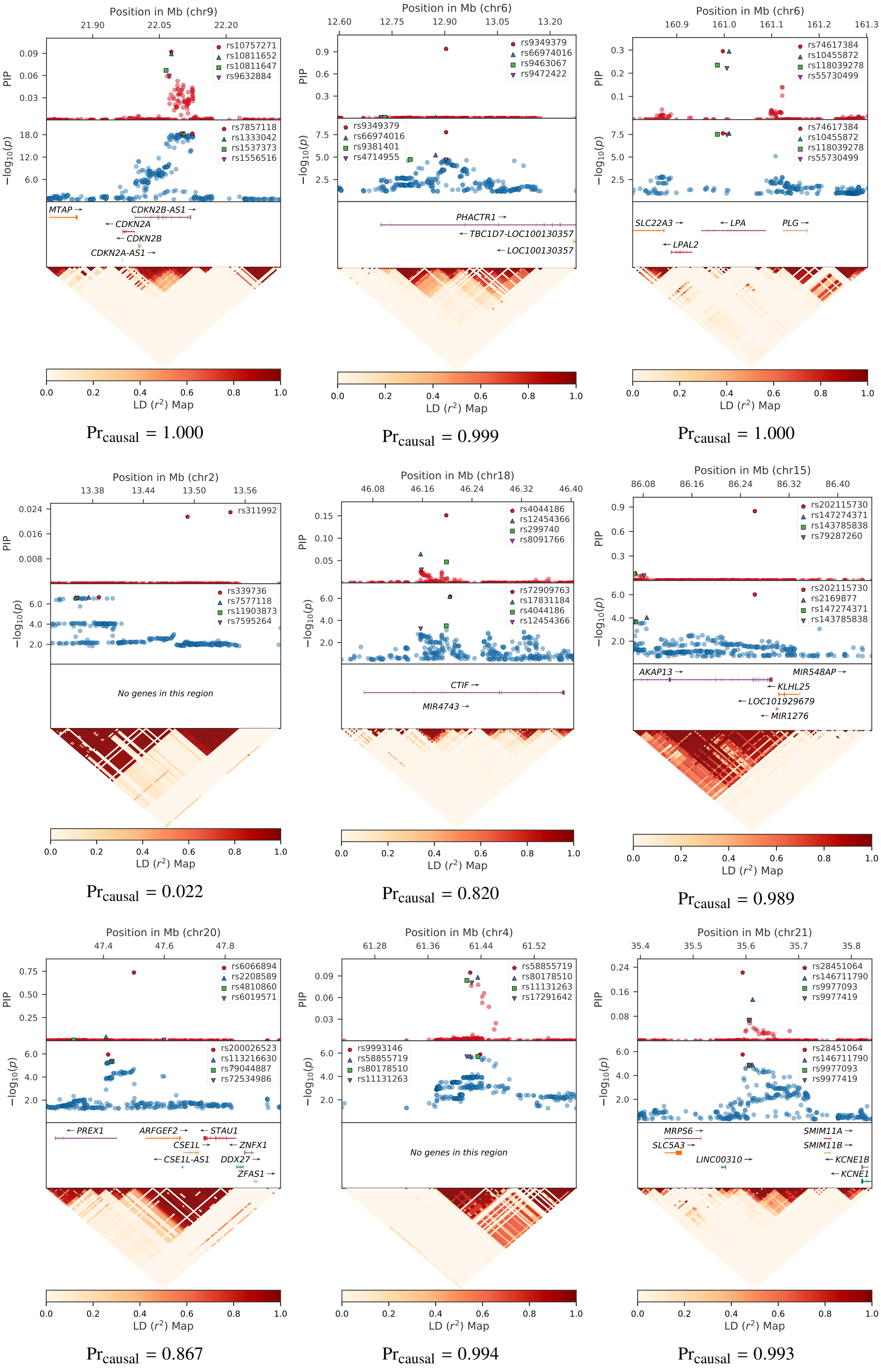

Position in Mb (chr9)
21.90 22.05 22.20
PIP
rs10757271
rs10811652
rs10811647
rs9632884
−log10(p)
rs7857118
rs1333042
rs1537373
rs1556516
MTAP →
CDKN2B-AS1 →
← CDKN2A
← CDKN2B
CDKN2A-AS1 →
LD (r2) Map
Prcausal = 1.000
Position in Mb (chr6)
12.60 12.75 12.90 13.05 13.20
rs9349379
rs66974016
rs9463067
rs9472422
rs9349379
rs66974016
rs9381401
rs4714955
PHACTR1 →
← TBC1D7-LOC100130357
← LOC100130357
Prcausal = 0.999
Position in Mb (chr6)
160.9 161.0 161.1 161.2 161.3
rs74617384
rs10455872
rs118039278
rs55730499
SLC22A3 →
← LPA
PLG →
← LPAL2
Prcausal = 1.000
Position in Mb (chr2)
13.38 13.44 13.50 13.56
rs311992
rs339736
rs7577118
rs11903873
rs7595264
No genes in this region
Prcausal = 0.022
Position in Mb (chr18)
46.08 46.16 46.24 46.32 46.40
rs4044186
rs12454366
rs299740
rs8091766
rs72909763
rs17831184
rs4044186
rs12454366
CTIF →
MIR4743 →
Prcausal = 0.820
Position in Mb (chr15)
86.08 86.16 86.24 86.32 86.40
rs202115730
rs147274371
rs143785838
rs79287260
rs202115730
rs2169877
rs147274371
rs143785838
AKAP13 →
MIR548AP →
← KLHL25
← LOC101929679
← MIR1276
Prcausal = 0.989
Position in Mb (chr20)
47.4 47.6 47.8
rs6066894
rs2208589
rs4810860
rs6019571
rs200026523
rs113216630
rs79044887
rs72534986
← PREX1
ARFGEF2 →
← STAU1
CSE1L →
← ZNFX1
← CSE1L-AS1
DDX27 →
ZFAS1 →
Prcausal = 0.867
Position in Mb (chr4)
61.28 61.36 61.44 61.52
rs58855719
rs80178510
rs11131263
rs17291642
rs9993146
rs58855719
rs80178510
rs11131263
No genes in this region
Prcausal = 0.994
Position in Mb (chr21)
35.4 35.5 35.6 35.7 35.8
rs28451064
rs146711790
rs9977093
rs9977419
rs28451064
rs146711790
rs9977093
rs9977419
MRPS6 →
SMIM11A →
SLC5A3 →
SMIM11B →
LINC00310 →
← KCNE1B
← KCNE1
Prcausal = 0.993

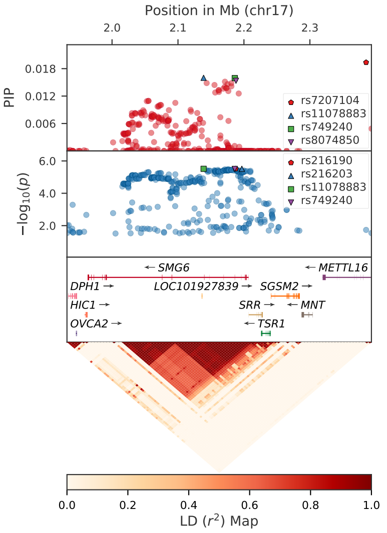

$\mathrm{Pr}_{\mathrm{causal}} = 0.999$

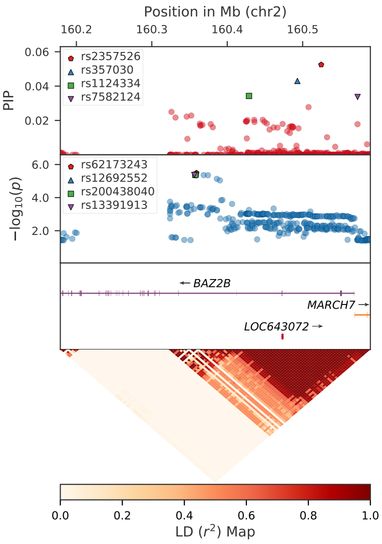

$\mathrm{Pr}_{\mathrm{causal}} = 0.905$

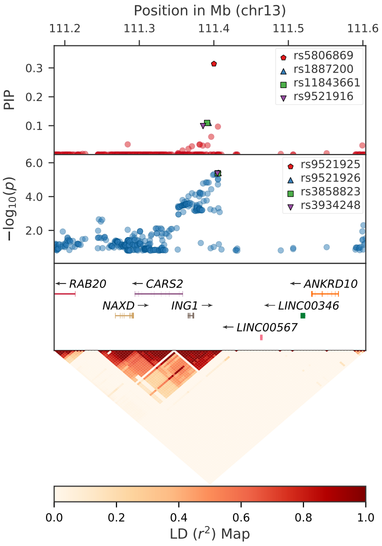

$\mathrm{Pr}_{\mathrm{causal}} = 0.994$

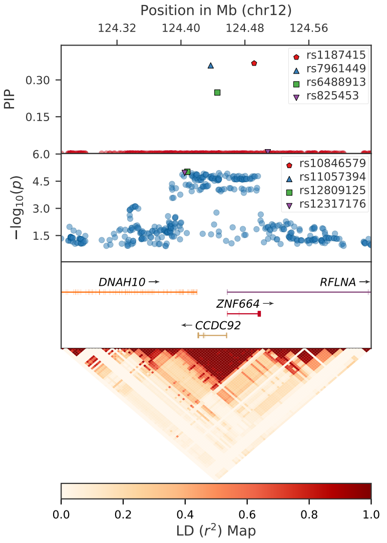

$\mathrm{Pr}_{\mathrm{causal}} = 0.976$

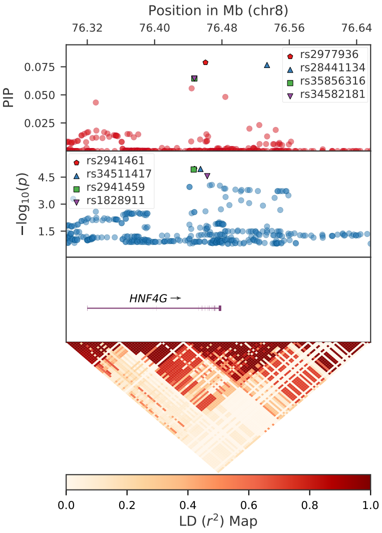

$\mathrm{Pr}_{\mathrm{causal}} = 0.699$

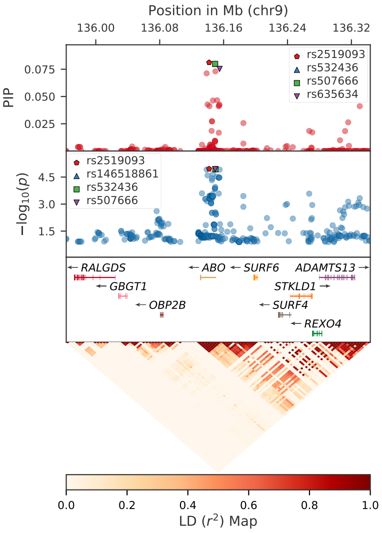

$\mathrm{Pr}_{\mathrm{causal}} = 0.994$

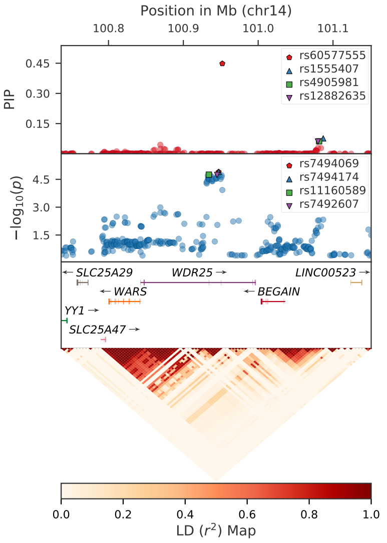

$\mathrm{Pr}_{\mathrm{causal}} = 0.832$

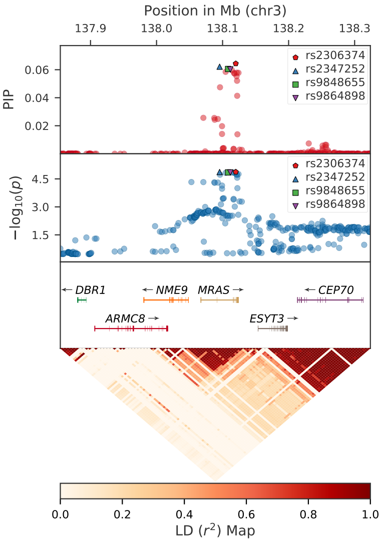

$\mathrm{Pr}_{\mathrm{causal}} = 0.973$

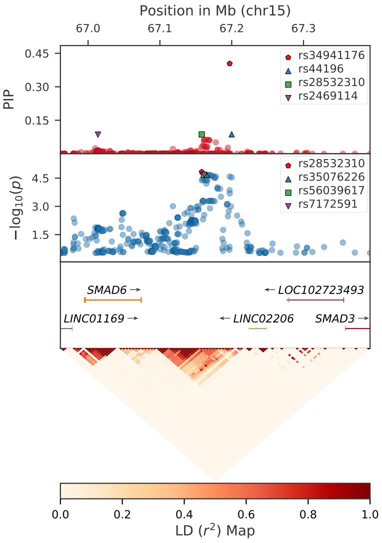

$\mathrm{Pr}_{\mathrm{causal}} = 0.999$

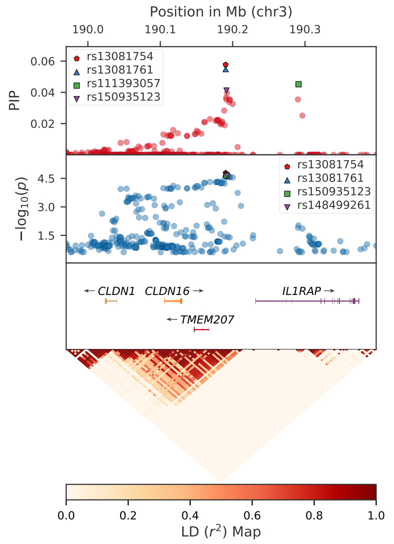

$\mathrm{Pr}_{\mathrm{causal}} = 0.988$

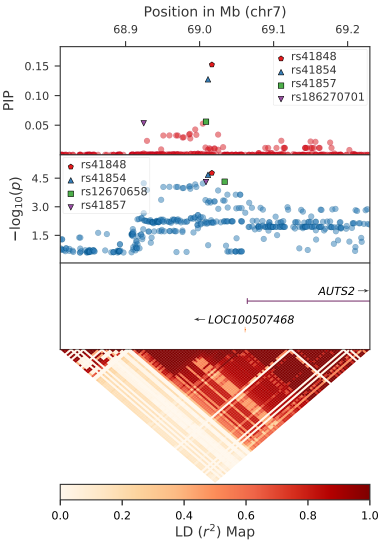

$\mathrm{Pr}_{\mathrm{causal}} = 0.981$

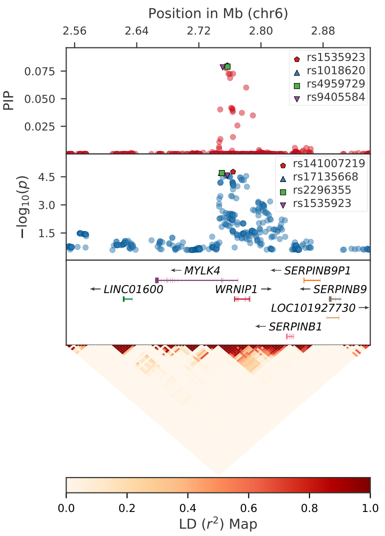

$\mathrm{Pr}_{\mathrm{causal}} = 0.989$

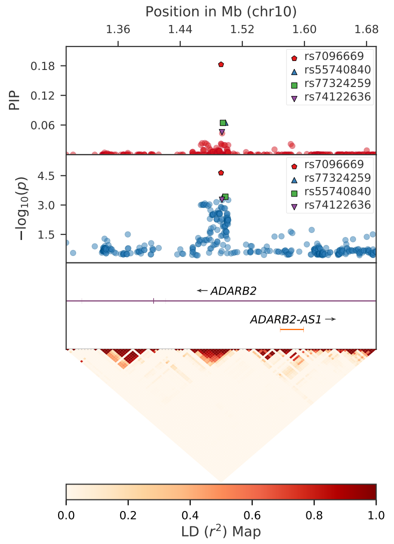

$\mathrm{Pr}_{\mathrm{causal}} = 0.863$

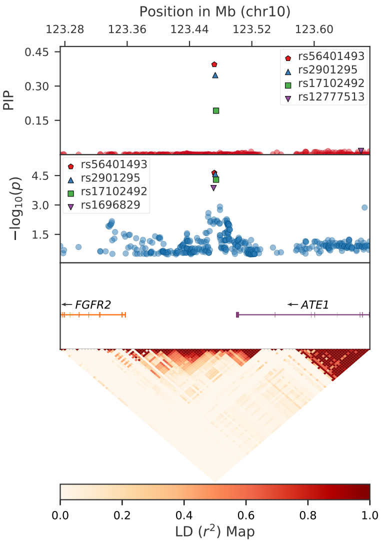

$\mathrm{Pr}_{\mathrm{causal}} = 0.953$

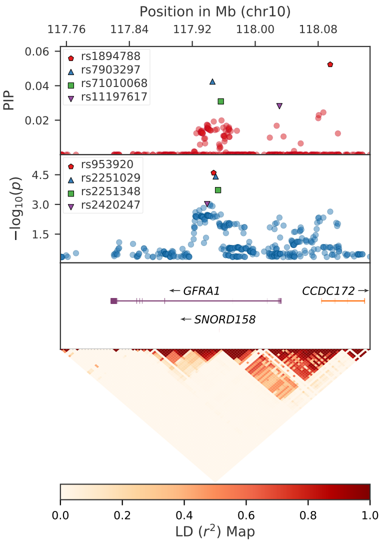

$\mathrm{Pr}_{\mathrm{causal}} = 0.677$

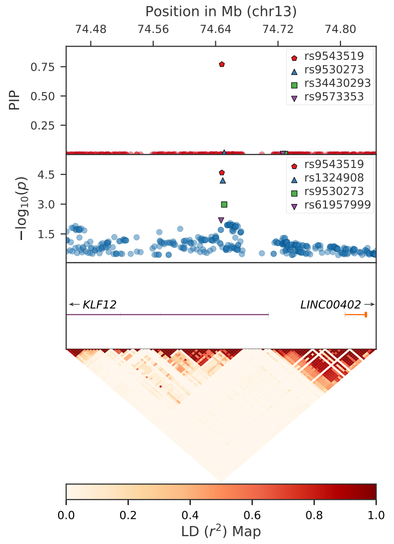

$\mathrm{Pr}_{\mathrm{causal}} = 0.798$

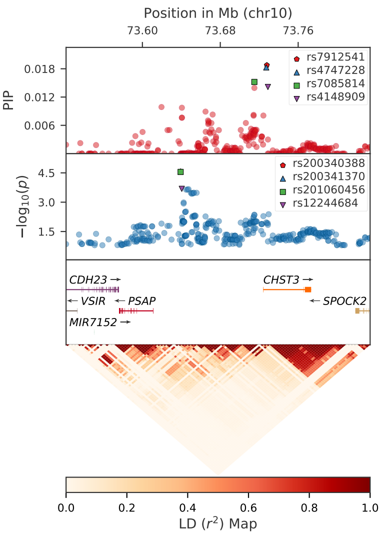

$\mathrm{Pr}_{\mathrm{causal}} = 0.506$

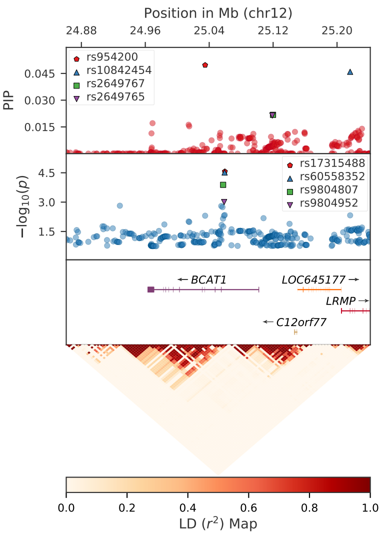

$\mathrm{Pr}_{\mathrm{causal}} = 0.692$

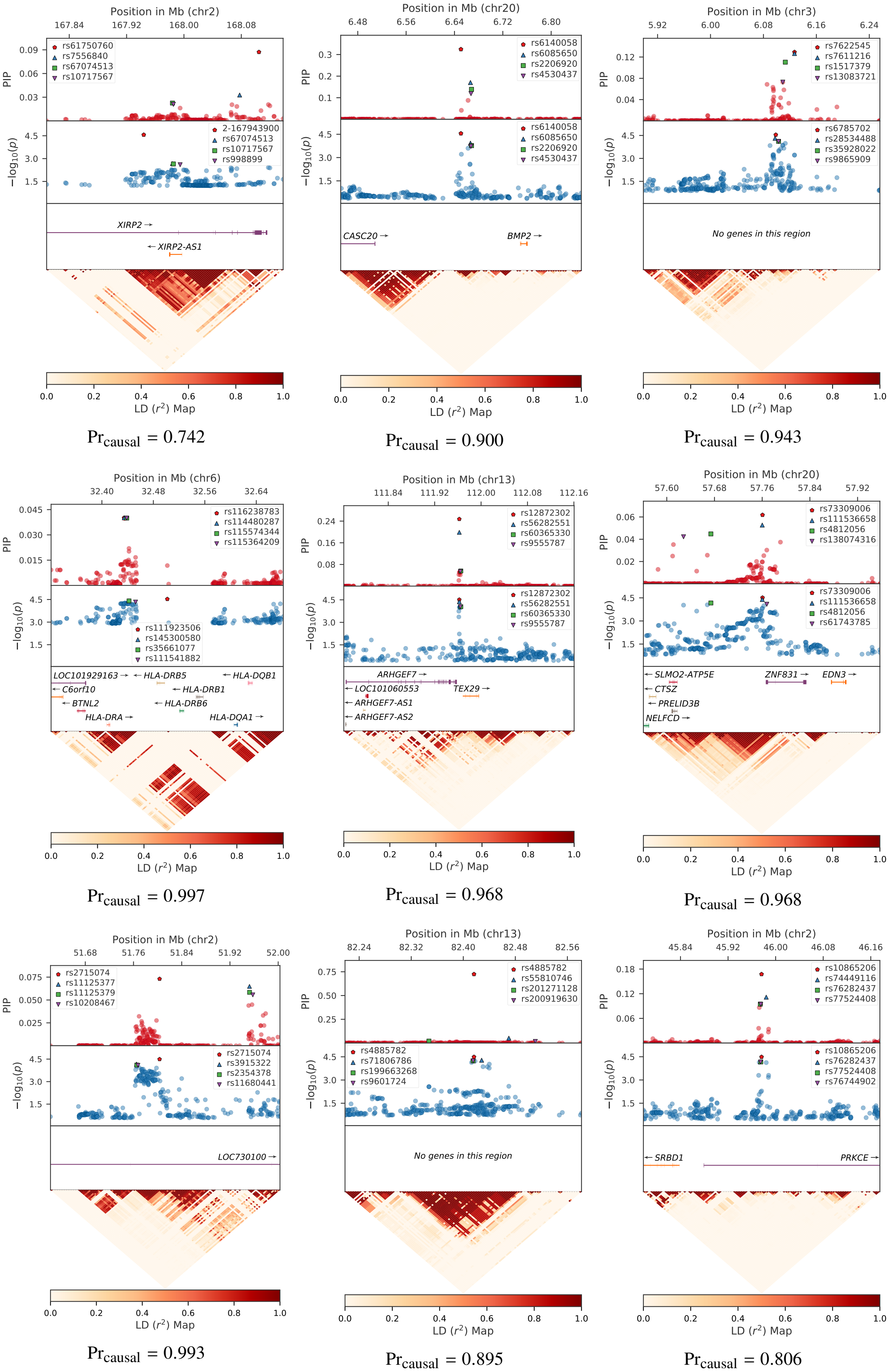

Position in Mb (chr2)
rs61750760
rs7556840
rs67074513
rs10717567
2-167943900
rs67074513
rs10717567
rs998899
XIRP2 →
← XIRP2-AS1
LD (r^2) Map
$\mathrm{Pr}_{\mathrm{causal}} = 0.742$
Position in Mb (chr20)
rs6140058
rs6085650
rs2206920
rs4530437
CASC20 →
BMP2 →
LD (r^2) Map
$\mathrm{Pr}_{\mathrm{causal}} = 0.900$
Position in Mb (chr3)
rs7622545
rs7611216
rs1517379
rs13083721
rs6785702
rs28534488
rs35928022
rs9865909
No genes in this region
LD (r^2) Map
$\mathrm{Pr}_{\mathrm{causal}} = 0.943$
Position in Mb (chr6)
rs116238783
rs114480287
rs115574344
rs115364209
rs111923506
rs145300580
rs35661077
rs111541882
LOC101929163 →
← HLA-DRB5
← HLA-DQB1
← C6orf10
← HLA-DRB1
← BTNL2
← HLA-DRB6
HLA-DRA →
HLA-DQA1 →
LD (r^2) Map
$\mathrm{Pr}_{\mathrm{causal}} = 0.997$
Position in Mb (chr13)
rs12872302
rs56282551
rs60365330
rs9555787
ARHGEF7 →
← LOC101060553
TEX29 →
← ARHGEF7-AS1
← ARHGEF7-AS2
LD (r^2) Map
$\mathrm{Pr}_{\mathrm{causal}} = 0.968$
Position in Mb (chr20)
rs73309006
rs111536658
rs4812056
rs138074316
rs73309006
rs111536658
rs4812056
rs61743785
← SLMO2-ATP5E
ZNF831 →
EDN3 →
← CTSZ
← PRELID3B
NELFCD →
LD (r^2) Map
$\mathrm{Pr}_{\mathrm{causal}} = 0.968$
Position in Mb (chr2)
rs2715074
rs11125377
rs11125379
rs10208467
rs2715074
rs3915322
rs2354378
rs11680441
LOC730100 →
LD (r^2) Map
$\mathrm{Pr}_{\mathrm{causal}} = 0.993$
Position in Mb (chr13)
rs4885782
rs55810746
rs201271128
rs200919630
rs4885782
rs71806786
rs199663268
rs9601724
No genes in this region
LD (r^2) Map
$\mathrm{Pr}_{\mathrm{causal}} = 0.895$
Position in Mb (chr2)
rs10865206
rs74449116
rs76282437
rs77524408
rs10865206
rs76282437
rs77524408
rs76744902
← SRBD1
PRKCE →
LD (r^2) Map
$\mathrm{Pr}_{\mathrm{causal}} = 0.806$

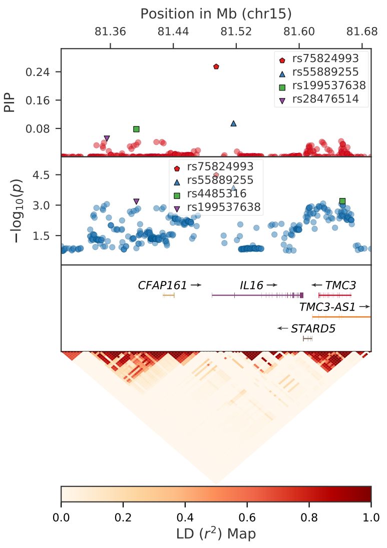

$\mathrm{Pr}_{\mathrm{causal}} = 0.994$

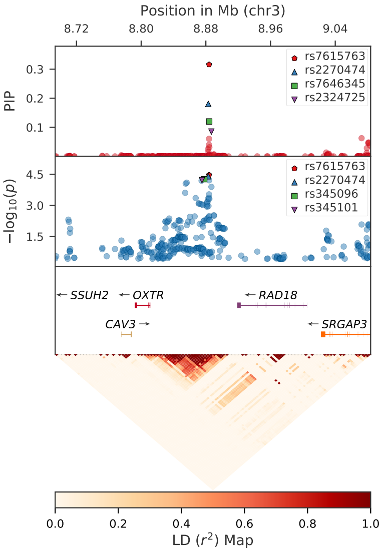

$\mathrm{Pr}_{\mathrm{causal}} = 0.938$

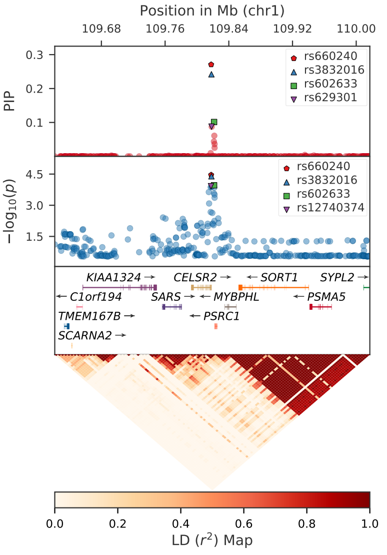

$\mathrm{Pr}_{\mathrm{causal}} = 0.965$

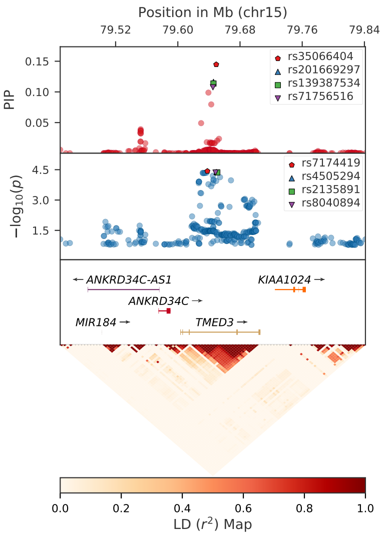

$\mathrm{Pr}_{\mathrm{causal}} = 0.957$

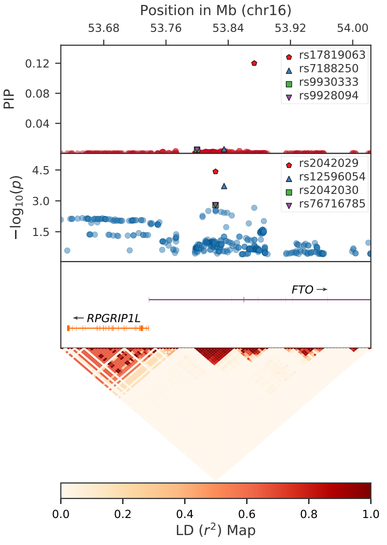

$\mathrm{Pr}_{\mathrm{causal}} = 0.223$

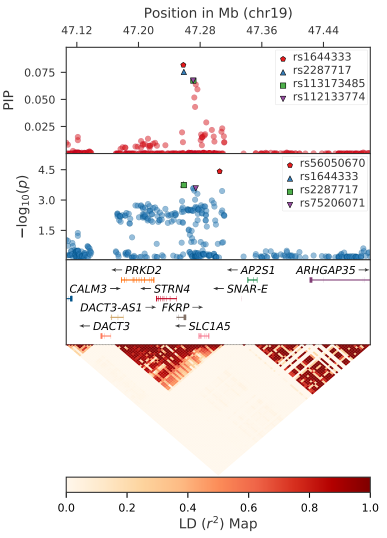

$\mathrm{Pr}_{\mathrm{causal}} = 0.887$

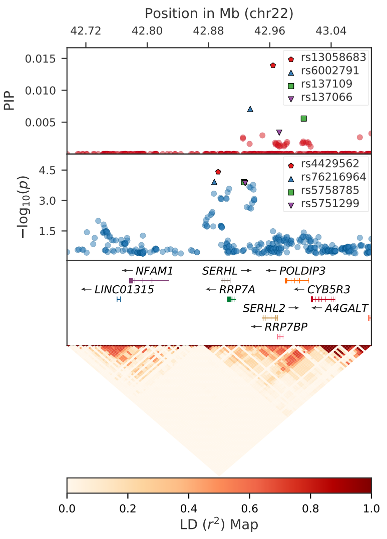

$\mathrm{Pr}_{\mathrm{causal}} = 0.065$

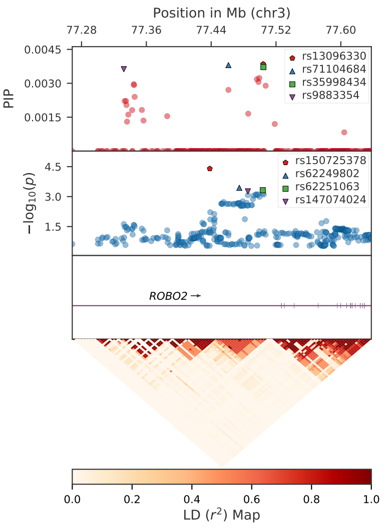

$\mathrm{Pr}_{\mathrm{causal}} = 0.058$

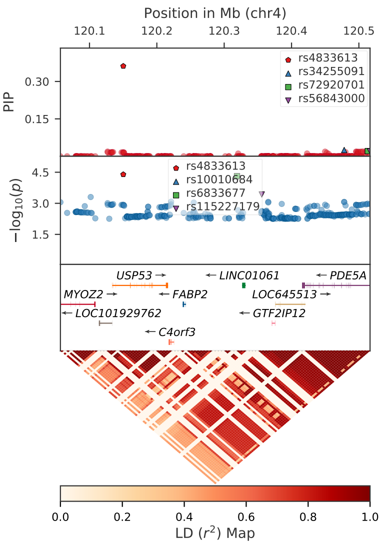

$\mathrm{Pr}_{\mathrm{causal}} = 0.883$

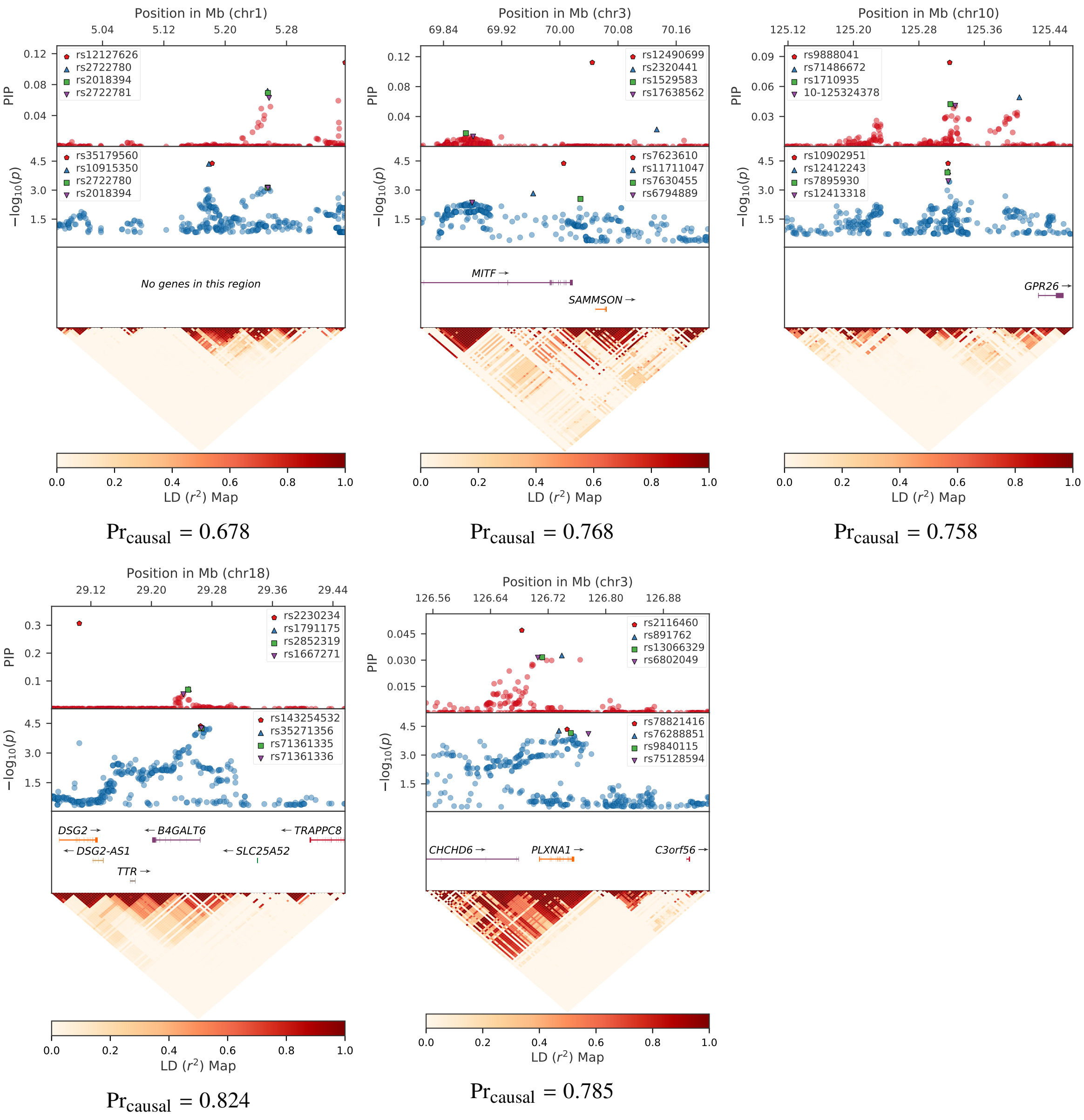

Figure S10. Finemapping of 50 genetic loci using B-LORE meta-analysis from 5 small GWAS (GerMIFS I-V). We first used meta-analysis of genome-wide SNPTEST summary statistics on these 5 small GWAS to select the top 50 loci and then applied B-LORE on these loci assuming a maximum of five causal SNPs per locus. Each plot has 4 panels from top to bottom: (1) The top panel shows the posterior inclusion probability (PIP) of each SNP obtained from B-LORE. (2) The second panel shows the $-\log_{10}(p)$ values obtained from SNPTEST / META. (3) The third panel shows the genes present in the region. (4) The bottom panel shows the LD map between the SNPs of the region. The top 4 SNPs obtained from B-LORE and SNPTEST / META are highlighted and annotated in the legend of each plot. Below each plot, we mention the probability ($\mathrm{Pr}_{\mathrm{causal}}$) of the locus containing at least one causal SNP.
