# Supplementary material for: Bayesian multiple logistic regression for case-control GWAS
Source: PLoS Genet. 2018 Dec 31;14(12):e1007856. doi: 10.1371/journal.pgen.1007856 (PMC6329526; doi:10.1371/journal.pgen.1007856)
Supplement: S1 Table — Table of top 50 genetic loci used in the meta-analysis of CAD with the GerMIFS I–V GWA studies. Here, we summarize the location, literature classification and significance score for association of these loci with CAD. The columns are: ‘Chr’ is the chromosome number on which the locus is located, ‘Start’ is the starting base pair position of the locus on the chromosome, ‘End’ is the end base pair position of the locus on the chromosome, Prcausal is the probability of the locus harboring a causal SNP as detected by B-LORE, −log10(p) is obtained from simple regression using SNPTEST/META, ‘Relevant genes’ are the list of genes which were found to be associated with CAD in prior studies (if no association is found then all genes are listed and if the locus is in a gene desert then no genes are listed), ‘Prior evidence’ gives a brief description of the prior study which showed CAD association. (PDF) [file pgen.1007856.s012.pdf]

Table S1. Association of genetic loci with CAD. Table of top 50 genetic loci used in the metaanalysis of CAD with the GerMIFS I-V GWA studies. Here, we summarize the location, literature classification and significance score for association of these loci with CAD. The columns are: 'Chr' is the chromosome number on which the locus is located, 'Start' is the starting base pair position of the locus on the chromosome, 'End' is the end base pair position of the locus on the chromosome, $\mathrm{Pr}_{\mathrm{causal}}$ is the probability of the locus harboring a causal SNP as detected by B-LORE, $-\log_{10}(p)$ is obtained from simple regression using SNPTEST/META, 'Relevant genes' are the list of genes which were found to be associated with CAD in prior studies (if no association is found then all genes are listed and if the locus is in a gene desert then no genes are listed), 'Prior evidence' gives a brief description of the prior study which showed CAD association.

Chr	Start	End	$-\log_{10}(p)$	$\mathrm{Pr}_{\mathrm{causal}}$	Relevant genes	Prior evidence
9	21796220	22322430	18.300	1.000	(9p21)	CARDIoGRAMplusC4D [1]
6	160807023	161302311	7.621	1.000	SLC22A3-LPAL2-LPA, PLG	CARDIoGRAMplusC4D [1]
6	12595148	13274123	7.770	0.999	PHACTR1	CARDIoGRAMplusC4D [1]
17	1931655	2393319	5.515	0.999	SMG6	CARDIoGRAMplusC4D [1]
15	66961485	67392656	4.830	0.999	SMAD6, SMAD3	CARDIoGRAMplusC4D within 0.4Mb [1]
6	32321004	32682043	4.530	0.997	C6orf10-BTNL2, HLA region	CAD GWAS in Han Chinese [2]
4	61230437	61583325	5.876	0.994	–	–
13	111185709	111603888	5.403	0.994	COL4A1	CARDIoGRAMplusC4D within 0.4Mb [1]
9	135963011	136343647	4.955	0.994	ABO	CARDIoGRAMplusC4D [1]
15	81297420	81691526	4.485	0.994	IL-16, C15orf16	CAD GWAS in Han Chinese [3,4]
21	35394714	35838907	5.753	0.993	KCNE2	CARDIoGRAMplusC4D [1]
2	51622258	52002985	4.494	0.993	– (nearest gene NRXN1)	CAD GWAS in OHGS1 + WTCCC2 [5]
15	86063681	86455957	6.014	0.989	AKAP13	Blood pressure [6]
6	2549217	2940800	4.766	0.989	MYLK4	Downregulated in heart failure patients [7]
3	189969941	190398385	4.783	0.988	IL-1RAP	Mediator of IL-33 which is associated with myocardial infarction [8]
7	68811704	69230066	4.772	0.981	AUTS2	–

Continued on next page

Table S1 – *Continued from previous page*

Chr	Start	End	$-\log_{10}(p)$	$\mathrm{Pr}_{\mathrm{causal}}$	Relevant genes	Prior evidence
12	124250106	124638005	5.022	0.976	ZNF664, CCDC92, DNAH10	Multiple risk factors (namely, high-density lipoprotein cholesterol level, triglycerides levels and waist-to-hip ratio) [9–11]
3	137854258	138323836	4.873	0.973	MRAS	CARDIoGRAMplusC4D [1]
13	111763359	112160416	4.512	0.969	ARHGEF1	Blood pressure [12]
20	57560777	57957389	4.511	0.968	GNAS-EDN3	Blood pressure [13]
1	109621436	110017105	4.460	0.965	SORT1	CARDIoGRAMplusC4D [1]
15	79448704	79841301	4.427	0.957	ADAMTS7	CARDIoGRAMplusC4D within 0.4Mb [1]
10	123274062	123671522	4.638	0.953	FGFR2, ATE1	–
3	5898557	6255997	4.543	0.943	–	–
3	8693649	9083779	4.470	0.938	SSUH2, CAV3, OXTR, RAD18	–
2	160178699	160589777	5.488	0.905	BAZ2B	Sudden cardiac arrest [14]
20	6451752	6849922	4.552	0.900	CASC20	Obesity [10]
13	82218231	82581703	4.491	0.895	PTMAP5 pseudogene	–
19	47106264	47500762	4.414	0.887	Many genes in the region	
4	120056815	120516597	4.393	0.883	USP53, MYOZ2, C4orf3, FABP2	–
20	47211801	47982128	5.948	0.867	PREX1, ARFGEF2	–
10	1293100	1692433	4.642	0.863	ADARB2	Blood pressure [15]
14	100736719	101151030	4.901	0.832	SLC25A29, SLC25A47, WARS, WDR25	–
18	29068068	29455957	4.364	0.824	DSG2	Arrhythmogenic right ventricular cardiomyopathy [16]
18	46029300	46404281	6.165	0.820	CTIF	–
2	45777620	46175313	4.487	0.806	SRBD1, PRKCE	–
13	74448453	74845794	4.585	0.798	KLF12	Sudden cardiac arrest [17]
3	126550576	126943740	4.341	0.785	CHCHD6, PLXNA1	–
3	69808638	70205194	4.373	0.768	MITF	–

Continued on next page

Table S1 – *Continued from previous page*

Chr	Start	End	$-\log_{10}(p)$	$\text{Pr}_{\text{causal}}$	Relevant genes	Prior evidence
10	125115722	125468274	4.371	0.758	–	–
2	167808448	168138372	4.562	0.742	XIRP2	–
8	76294909	76656609	4.960	0.699	HNF4G	Obesity [18]
12	24861033	25241643	4.565	0.692	BCAT1, LRMP, CASC1	–
1	4980475	5356571	4.383	0.678	–	–
10	117752333	118145972	4.595	0.677	GFRA1, CCDC172, PNLIPRP3	–
10	73521578	73833908	4.570	0.506	CDH23, PSAP, CHST3	–
16	53624847	54023335	4.420	0.223	FTO	Obesity [19]
22	42695345	43092556	4.408	0.065	NFAM1, SERHL, RRP7A, RRP7B, POLDIP3, CYB5R3	CyB5R3 regulates nitric oxide induced soluble guanylate cyclase activation, which is linked to cardiovascular diseases (CVD) [20]
3	77268577	77637652	4.399	0.058	ROBO2	–
2	13330532	13602018	6.660	0.022	–	–

References

1. CARDIoGRAMplusC4D. A comprehensive 1000 Genomes-based genome-wide association meta-analysis of coronary artery disease. Nature Genetics. 2015;47(10):1121–1130. doi:10.1038/ng.3396.

2. Lu X, Wang L, Chen S, He L, Yang X, Shi Y, et al. Genome-wide association study in Han Chinese identifies four new susceptibility loci for coronary artery disease. Nature Genetics. 2012;44(8):890–894. doi:10.1038/ng.2337.

3. Huang H, Zeng Z, Zhang L, Liu R, Li X, Qiang O, et al. The association of interleukin-16 gene polymorphisms with susceptibility of coronary artery disease. Clinical Biochemistry. 2013;46(3):241–244. doi:10.1016/j.clinbiochem.2012.11.009.

4. Grönberg C, Bengtsson E, Fredrikson GN, Nitulescu M, Asciutto G, Persson A, et al. Human carotid plaques with high levels of interleukin-16 are associated with reduced risk for cardiovascular events. Stroke. 2015;46(10):2748–2754. doi:10.1161/STROKEAHA.115.009910.

5. Stewart AF, Dandona S, Fan M, Almontashiri N, Chen L, Davies RW, et al. Identification of a rare variant near Neurexin 1 associated with coronary artery disease. Circulation. 2015;122(Suppl 21):17262.

6. Hong KW, Lim JE, Oh B. A regulatory SNP in AKAP13 is associated with blood pressure in Koreans. J Hum Genet. 2011;56(3):205–210. doi:10.1038/jhg.2010.167.

7. Herrer I, Rosello-Lleti E, Rivera M, Molina-Navarro MM, Tarazon E, Ortega A, et al. RNA-sequencing analysis reveals new alterations in cardiomyocyte cytoskeletal genes in patients with heart failure. Lab Invest. 2014;94(6):645–653. doi:10.1038/labinvest.2014.54.

8. Liew FY, Pitman NI, McInnes IB. Disease-associated functions of IL-33: the new kid in the IL-1 family. Nature Review Immunology. 2010;10(2):103–110. doi:10.1038/nri2692.

9. Teslovich TM, Musunuru K, Smith AV, Edmondson AC, Stylianou IM, Koseki M, et al. Biological, clinical and population relevance of 95 loci for blood lipids. Nature. 2010;466(7307):707–713. doi:10.1038/nature09270.

10. Shungin D, Winkler TW, Croteau-Chonka DC, Ferreira T, Locke AE, Magi R, et al. New genetic loci link adipose and insulin biology to body fat distribution. Nature. 2015;518(7538):187–196. doi:10.1038/nature14132.

11. Dastani Z, Marie-France H, Nicholas T, B PJR, Xin Y, A SR, et al. Novel loci for adiponectin levels and their influence on type 2 diabetes and metabolic traits: A multi-ethnic meta-analysis of 45,891 individuals. PLOS Genetics. 2012;8(3):1–23. doi:10.1371/journal.pgen.1002607.

12. Guilluy C, Bregeon J, Toumaniantz G, Rolli-Derkinderen M, Retailleau K, Loufrani L, et al. The Rho exchange factor Arhgef1 mediates the effects of angiotensin II on vascular tone and blood pressure. Nat Med. 2010;16(2):183–190. doi:10.1038/nm.2079.

13. for Blood Pressure Genome-Wide Association Studies TIC. Genetic variants in novel pathways influence blood pressure and cardiovascular disease risk. Nature. 2011;478(7367):103–109. doi:10.1038/nature10405.

14. Arking DE, Juhani JM, Philippe G, Adriana HV, Mark E, T BM, et al. Identification of a sudden cardiac death susceptibility locus at 2q24.2 through genome-wide association in European ancestry individuals. PLOS Genetics. 2011;7(6):1–9. doi:10.1371/journal.pgen.1002158.

15. Sung YJ, de las Fuentes L, Schwander KL, Simino J, Rao DC. Gene-smoking interactions identify several novel blood pressure loci in the Framingham heart study. American Journal of Hypertension. 2015;28(3):343. doi:10.1093/ajh/hpu149.

16. Rasmussen TB, Palmfeldt J, Nissen PH, Magnoni R, Dalager S, Jensen UB, et al. Mutated Desmoglein-2 Proteins are Incorporated into Desmosomes and Exhibit Dominant-Negative Effects in Arrhythmogenic Right Ventricular Cardiomyopathy. Human Mutation. 2013;34(5):697–705. doi:10.1002/humu.22289.

17. Aouizerat BE, Vittinghoff E, Musone SL, Pawlikowska L, Kwok PY, Olgin JE, et al. GWAS for discovery and replication of genetic loci associated with sudden cardiac arrest in patients with coronary artery disease. BMC Cardiovascular Disorders. 2011;11(1):29. doi:10.1186/1471-2261-11-29.

18. Berndt SI, Gustafsson S, Magi R, Ganna A, Wheeler E, Feitosa MF, et al. Genome-wide meta-analysis identifies 11 new loci for anthropometric traits and provides insights into genetic architecture. Nature Genetics. 2013;45(5):501–512. doi:10.1038/ng.2606.

19. Liu C, Sihua M, Chunqin P. The FTO gene rs9939609 polymorphism predicts risk of cardiovascular disease: A systematic review and meta-analysis. PLOS ONE. 2013;8(8):1–7. doi:10.1371/journal.pone.0071901.

20. Rahaman MM, Nguyen AT, Miller MP, Hahn SA, Sparacino-Watkins C, Jobbagy S, et al. Cytochrome b5 reductase 3 modulates soluble guanylate cyclase redox state and cGMP signaling. Circulation Research. 2017;121(2):137–148. doi:10.1161/CIRCRESAHA.117.310705.
